# Addition of Alanyl-Glutamine to Dialysis Fluid Restores Peritoneal Cellular Stress Responses – A First-In-Man Trial

**DOI:** 10.1371/journal.pone.0165045

**Published:** 2016-10-21

**Authors:** Klaus Kratochwill, Michael Boehm, Rebecca Herzog, Katharina Gruber, Anton Michael Lichtenauer, Lilian Kuster, Dagmar Csaicsich, Andreas Gleiss, Seth L. Alper, Christoph Aufricht, Andreas Vychytil

**Affiliations:** 1 Division of Pediatric Nephrology and Gastroenterology, Department of Pediatrics and Adolescent Medicine, Medical University of Vienna, Vienna, Austria; 2 Christian Doppler Laboratory for Molecular Stress Research in Peritoneal Dialysis, Department of Pediatrics and Adolescent Medicine, Medical University of Vienna, Vienna, Austria; 3 Zytoprotec GmbH, Vienna, Austria; 4 Center for Medical Statistics, Informatics, and Intelligent Systems, Medical University of Vienna, Vienna, Austria; 5 Division of Nephrology and Center for Vascular Biology Research, Beth Israel Deaconess Medical Center, Harvard Medical School, Boston, Massachusetts, United States of America; 6 Division of Nephrology and Dialysis, Medical University of Vienna, Vienna, Austria; Medizinische Universitat Graz, AUSTRIA

## Abstract

**Background:**

Peritonitis and ultrafiltration failure remain serious complications of chronic peritoneal dialysis (PD). Dysfunctional cellular stress responses aggravate peritoneal injury associated with PD fluid exposure, potentially due to peritoneal glutamine depletion. In this randomized cross-over phase I/II trial we investigated cytoprotective effects of alanyl-glutamine (AlaGln) addition to glucose-based PDF.

**Methods:**

In a prospective randomized cross-over design, 20 stable PD outpatients underwent paired peritoneal equilibration tests 4 weeks apart, using conventional acidic, single chamber 3.86% glucose PD fluid, with and without 8 mM supplemental AlaGln. Heat-shock protein 72 expression was assessed in peritoneal effluent cells as surrogate parameter of cellular stress responses, complemented by metabolomics and functional immunocompetence assays.

**Results:**

AlaGln restored peritoneal glutamine levels and increased the primary outcome heat-shock protein expression (effect 1.51-fold, CI 1.07–2.14; p = 0.022), without changes in peritoneal ultrafiltration, small solute transport, or biomarkers reflecting cell mass and inflammation. Further effects were glutamine-like metabolomic changes and increased *ex-vivo* LPS-stimulated cytokine release from healthy donor peripheral blood monocytes. In patients with a history of peritonitis (5 of 20), AlaGln supplementation decreased dialysate interleukin-8 levels. Supplemented PD fluid also attenuated inflammation and enhanced stimulated cytokine release in a mouse model of PD-associated peritonitis.

**Conclusion:**

We conclude that AlaGln-supplemented, glucose-based PD fluid can restore peritoneal cellular stress responses with attenuation of sterile inflammation, and may improve peritoneal host-defense in the setting of PD.

## Introduction

Global numbers of prevalent patients in need of renal replacement therapy are expected to grow exponentially over the next years [1–3]. Although peritoneal dialysis (PD) might provide a means to address this challenge, the therapy requires repeated exposure of the peritoneum to glucose-based, hyperosmolar PD fluid (PDF). Bio-incompatible PDF injures peritoneal mesothelial cells, which constitute both the physical barrier and the exchange membrane for the dialysis process. Bio-incompatible PDF also injures both free-floating and sessile peritoneal leukocytes which constitute the first defense against peritoneal infection [4, 5]. The repeated metabolic and biomechanical insults arising from serial PDF exposures lead to smoldering inflammation and reduced host defense in the peritoneal cavity [6–10]. The interplay of PDF cytotoxicity and intermittent bacterial infections is believed to contribute to clinical complications of PD therapy, such as membrane failure and peritonitis [11]. Recent meta-analyses revealed no significant influence of newer varieties of biocompatible PDF on peritonitis rate or peritoneal membrane function [12, 13].

Our previous research demonstrated that exposure to PDF in experimental *in-vitro* and *in-vivo* models of PD results not only in cytotoxic injury but also in counteracting cytoprotective stress responses (CSR) in peritoneal cells [14–16]. The CSR comprise a molecular machinery that is remarkably conserved from simple bacteria to higher organisms, with heat shock proteins (HSP) as their prototypical effector proteins [17, 18]. The CSR mechanisms are evolutionary designed to detect deviations from the normal physiological equilibrium and stabilize protein integrity or facilitate organized degradation. The HSP, which can make up for as much as 5% of the total cellular protein content under stressful conditions, have been shown to cooperate in a plethora of biological processes, including pro- and anti-inflammatory mechanisms, regulation of programmed cell death and redox homeostasis [19]. Exposure of cells to unphysiological PDF, however, is likely to result in inadequate responses. Acute exposure to PDF elicits highly variable CSR [14, 20, 21] which at first view correlate with strength of cytotoxic stimulus and, therefore, with bio-incompatibility of instilled PDF [22]. Enhancing CSR resulted in improved PDF tolerance and resistance of mesothelial cells in *in-vitro* models, and in improved peritoneal membrane integrity in *in-vivo* models of experimental PD [15, 23, 24]. However, the more closely experimental models of PD mimic the clinical situation (*e*.*g*. longer duration of PDF exposure), the more they are characterized by inadequate CSR, with dampening of potentially protective biochemical pathways and induction of potentially deleterious injury induced sterile inflammation [25, 26].

In the intensive care setting, both CSR and patient outcome were shown to deteriorate in parallel with systemic glutamine (Gln) depletion and to improve with Gln supplementation [27–29]. A recent Cochrane meta-analysis of 53 randomized controlled trials concluded that Gln supplementation reduced infection rate and length of hospital stay in critically ill patients [30]. The relevant cellular processes underlying dysfunctional CSR and impaired host defense in these patients and in patients undergoing PD are likely closely linked by a common pathomechanism, such as injury induced chronic inflammation [11]. We recently demonstrated that Gln depletion during PDF exposure aggravated mesothelial cell vulnerability to injury and suppressed CSR [25]. PDF supplementation with Gln or with its stable dipeptide, alanyl-glutamine (AlaGln), restored CSR and enhanced both heat shock protein (HSP) expression and cytoprotection in *in-vitro* and *in-vivo* models of PD [25, 31]. As AlaGln is already used clinically for parenteral nutrition, this approach is particularly attractive for translation from bench-to-bedside in PD.

Therefore, the aim of this first-in-man study was to assess whether established and recently reported effects of AlaGln can be translated from experimental *in-vitro* and *in-vivo* PD models into the clinical setting of PD. In particular, we tested whether AlaGln addition to standard glucose-based PDF restores or maintains CSR in peritoneal cells during a single 4-hour dwell.

## Methods

The study protocol was approved by the local ethics committee of the Medical University of Vienna (EK 867/2010 and EK 1167/2013), registered in www.clinicaltrials.gov (NCT01353638), and carried out in accord with the Declaration of Helsinki. This randomized, open-label, two-period cross-over study conducted at the Department of Nephrology, Medical University of Vienna Austria, recruited PD patients between May 2011 and March 2012. All patients provided written informed consent. PD patients aged ≥ 19 years were considered eligible by virtue of clinical stability during at least two months on continuous ambulatory PD (CAPD) or continuous cyclic PD (CCPD), without severe concomitant disease. Exclusion criteria included hypersensitivity to the study medication, malignancy requiring chemotherapy or radiation, pregnancy, limited efficacy of PD due to anatomical reasons, clinically significant inflammation and immunosuppressive therapy.

20 patients completed the study as planned (per protocol (PP) population) from among the first 21 enrolled patients (ITT population). Sample size calculation was based on the percent change in the primary outcome parameter “total HSP expression” in cells from PD effluents after treatment with AlaGln compared to a control group. A sample size of 28 patients in a cross-over study design was estimated to have 80% power to detect a difference in means of 30 percentage points, using a 0.05 two-sided significance level (nQuery Advisor version 6). This calculation included an expected 10% drop-out rate and an assumed standard deviation of within-subject period differences of 50 percentage points. Predicted HSP expression levels were estimated from previous results in the *ex-vivo* setting [25] and from the literature [32]. For this reason, an interim analysis was planned to assess actual standard deviation of HSP expression after clinical evaluation for the first 20 patients, in order to re-calculate and to adjust final sample size. The planned interim analysis allowed assessment of the primary outcome parameter in only 4 of the 20 patients, and led to premature termination of patient recruitment (see below “Analysis of HSP expression”).

Demographics, baseline data and underlying renal diseases are shown in Table 1. Five patients had episodes of peritonitis > 2 months before enrollment. Routine PD was performed with multi-chamber glucose-based PD fluids (Physioneal®, Gambrosol Trio® or Balance®) for short dwells. For long dwells, patients received (with one exception) icodextrin-based PD fluids (Extraneal®). Individual patient PD regimens were kept unchanged throughout the study period. The exchange immediately prior to the study PETs was of at least 4 hours' duration and was performed identically in all patients, using the same PD fluid concentration (3.86% glucose) to minimize inter-individual variability.

**Table 1:** Baseline characteristics of the study patients.

Variable			
Total number per protocol population (PPP)(n)^a^		20	
Sex			
	Male (n)	13	(65%)
Age (y)		58.0	(47.0–68.0)
Body mass index (kg/m^2^)		25.7	(23.2–28.2)
Ethnicity (n)			
	Caucasian /white	19	(95%)
	Asian	1	(5%)
Underlying Renal Disease			
	Diabetic nephropathy	3	(15%)
	Chronic interstitial nephritis	3	(15%)
	Obstructive nephropathy	2	(10%)
	Polycystic kidney disease	2	(10%)
	Renal vasculopathy^**b**^	2	(10%)
	Other causes	3	(15%)
	Unknown causes	5	(25%)
Duration of PD (years)		2.38	(0.80–3.46)
Patients with residual renal urine output (n)		14	(70%)
Residual renal urine output^c^ (ml)		1200	(0–1613)
Residual renal clearance (ml/min/1.73 m^2^)^c^^,^^d^		4.79	(0.00–6.98)
Automated PD (APD)		10	(50%)
Total weekly Kt/V		2.13	(1.82–2.32)
History of peritonitis (> 2 months previous)		5	(25%)
Transport type^e^			
	Low (L)	1	5%
	Low average (LA)	2	10%
	High average (HA)	11	55%
	High (H)	6	30%

Data are presented as frequency and percentage, as appropriate, and otherwise as median and interquartile range for the overall study population.

^a^ The ITT population (n = 21) differs from the PP population (n = 20) by a 75-year-old Caucasian female patient with diabetic kidney disease who was treated for > 5 years on PD before inclusion in the trial. None of the demographic factors in the PP population differed significantly between the sequences in the cross-over trial.

^b^ Including hypertensive nephropathy.

^c^ Median residual renal urine output and clearance were computed from all patients. Six patients had no residual renal output.

^d^ Residual renal clearance was calculated as mean of renal creatinine and renal urea clearance using 24 h urine samples.

^e^ Transport type was determined from the PET with standard 3.86% PDF.

**Fig 1:**
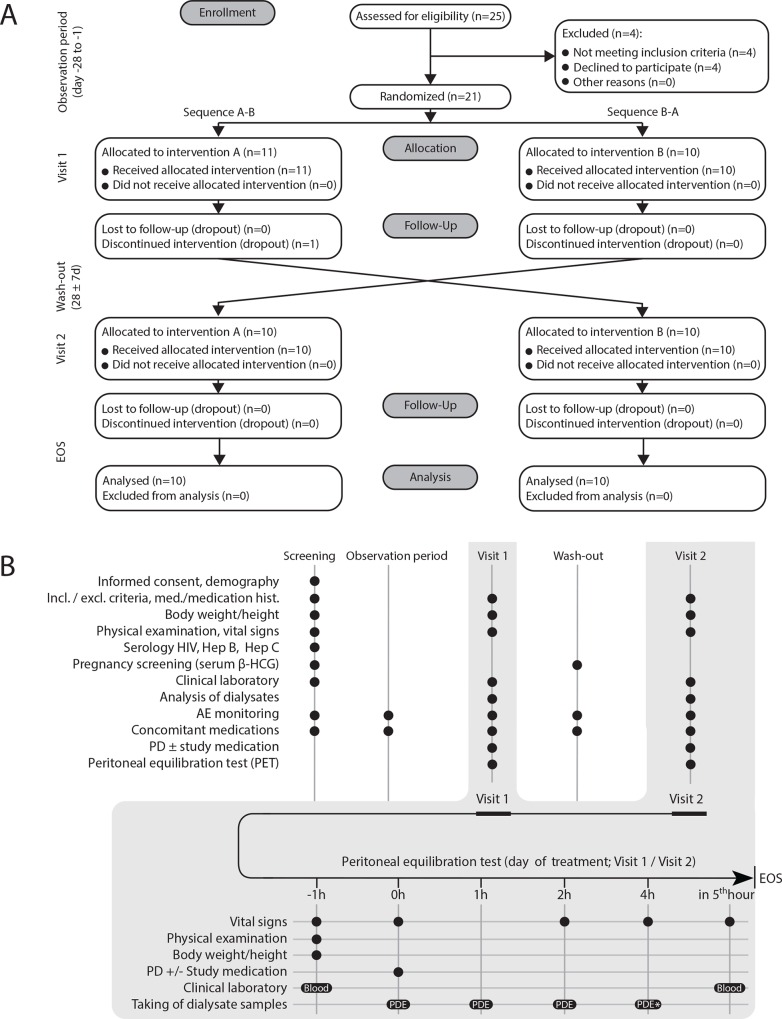
Study design. ESRD patients aged ≥ 19 years were included if stable for at least two months on continuous ambulatory PD (CAPD) or continuous cyclic PD (CCPD) and without severe concomitant disease. Exclusion criteria included hypersensitivity to study medication, malignancy requiring chemotherapy or radiation, pregnancy, limited PD efficacy secondary to anatomical considerations, clinically significant inflammation or current immunosuppressive therapy. After a positive screening visit and an observation period of four weeks, subjects were randomly allocated on an alternating basis to either sequence “A-B” (Sequence 1) or “B-A” (Sequence 2), stratified by sex, age (< or ≥ 60 years), time on PD (< or ≥ 1 year) and peritonitis history (yes or no). Randomization occurred immediately before the start of treatment period 1. Treatment visits 1 and 2 consisted of one single PD exchange performed as a 4-hour-peritoneal equilibration test (PET) using either standard PDF (Dianeal® PD4 with 3.86% glucose, Baxter) or standard PDF supplemented with 8 mM alanyl-glutamine dipeptide (AlaGln; Dipeptiven, Fresenius Kabi). The two treatments occurred in a random order and were separated by a wash-out period of 28–35 days. At times 0 (immediately after completion of PDF infusion), 1 h, 2 h, and 4 h, peritoneal samples were withdrawn. At 4 h, the remaining effluent was drained and collected. Blood was drawn 60 min before and 60 min following the PET. Four patients were excluded for reasons of sub-ileus and abdominal surgery (n = 1), catheter-related infection (n = 1), screening failure (n = 1) and deviation from protocol (no last bag before PET) (n = 1).

Following an observation period (for details, see Fig 1), patients were randomized immediately before start of treatment to either sequence “A-B” or “B-A”. Random allocation of treatments with AlaGln (= “A”) or without AlaGln (= “B”) in a ratio 1:1 was performed using Randomizer® (www.randomizer.at) and stratified by sex, age (< or ≥ 60 years), time on PD (< or ≥ 1 year) and peritonitis history (yes or no). The demographic factors of both sequences were statistically indistinguishable. The two treatments were separated by a wash-out period (28–35 days) to exclude carry-over effects [33]. None of the patients had relevant inflammatory episodes or abdominal surgery in the washout period. For AlaGln treatment, 17.4 ml Dipeptiven® (Fresenius-Kabi, 200 mg AlaGln/ml) was added to 2.0 L of PDF (Dianeal® PD4 with 3.86% glucose, Baxter) immediately before instillation, resulting in a final AlaGln concentration of 8 mM AlaGln (= 0.174%). The increase in total osmolality of the modified PDF was less than 1%, and PDF pH was unaltered in the range of 5.2–5.5.

Single PD exchanges (with and without AlaGln) were performed as 4 h PETs. Supine patients were infused with 2.0 L PDF. Samples were withdrawn immediately after completion of infusion (time 0 h) and at 1, 2 and 4 h after infusion. After completion of the 4 h dwell, peritoneal effluent was drained from the sitting patient and samples were taken. The difference between drainage and instillation volumes was recorded as ultrafiltration (correcting for sample volume removed during PET, bag over-fill with fresh PDF, and empty bag weight). The 3.86% PET allows calculation of peritoneal transport rates with accuracy comparable to the 2.27% PET, while providing more information on peritoneal membrane function such as sodium sieving [34, 35].

Biochemical measurements and transfer kinetics between peritoneal and systemic circulation were analyzed in serum samples (prior to each PET and 1 h thereafter) and dialysate specimens. Peritoneal cells were counted in the 4 h PET effluent, and cytospin slides (20,000 cells per slide) were stained to perform differential cell counts.

## Pharmacokinetics for glutamine, alanine and AlaGln

The EZ:Faast amino acid analysis (CE-IVD marked) procedure consists of a solid phase extraction step followed by derivatization of the extracted amino acids to form chloroformates. After a liquid/liquid extraction, the organic layer is taken to dryness under nitrogen. Aliquots of 50 μl of serum or dialysate were assessed using homoarginine, methionine-d3, and homophenylalanine as internal standards. Samples were reconstituted in 100 μl of 10 mM ammonium formate in water: 10 mM ammonium formate in methanol solution (1:2; v/v). Amino acids were separated using a Waters Quattromicro Liquid Chromatography system (Waters Corporation, Milford, MA, USA). Sample aliquots of 5 μl were injected onto a Phenomenex EZ:Faast AAA-MS column (250 mm × 2.0 mm) and eluted with a mixture of 1 mM ammonium formate and 10 mM ammonium formate in methanol by gradient elution. An amino acid standard mixture was used to create 5-point calibration curves and run at the beginning and end of the serum analysis. A QC sample consisted of the three internal standards. The mass spectrometer (Waters Corporation, Milford, MA, USA) was equipped with an electrospray source used in positive ionization mode (source temperature 120°C with a cone gas flow of 50 l/hr; desolvation temperature 350°C with a desolvation gas flow of 700 l/hr; capillary voltage 1000V). LC-MS Data Processing was performed using QuanLynx software version 4.1 (Waters Corporation) by comparing the peak area with the calibration curves of the internal standards. Responses for each of the amino acids were calculated relative to the internal standards.

## Analysis of HSP expression

Hsp72 expression in effluent peritoneal cell lysate was the study's primary endpoint. Peritoneal cells were isolated from PD effluents by gentle centrifugation (200 x g, 20 minutes, RT). The cell pellets were resuspended in M199 medium without FCS and the cell number of the suspension was evaluated by an automated cell counter (Cellometer, Nexcelcom, Lawrence, MA, USA). 4 cytospin preparations of each effluent sample were prepared, fixed, and stained for assessment of cell subpopulations. Differential cell counts of peritoneal effluents stained with eosin and azure (Hemacolor, Merck Millipore) were performed by an independent technician in a blinded manner.

Cellular HSP expression was assessed by standard Western blot analysis. Cells harvested from fresh peritoneal effluents at 240 min of the PET were lysed in 100 μl lysis buffer (30 mM Tris, pH 8.5, 7 M urea, 2 M thiourea, 4% 3-[(3-Cholamidopropyl) dimethylammonio]-1-propanesulfonate (CHAPS), 1 mM EDTA, 1 tablet of Complete Protease Inhibitor (Roche, Basel; Switzerland) and 1 tablet of phosphatase inhibitor (PhosSTOP, Roche) per 100 ml) per 1x10^6^ cells for 10 min at 25°C. The resulting lysates were centrifuged for 30 min (14,000 × g, 4°C) and stored at -80°C until further processing. Total protein concentration was determined using the 2D-Quant kit (GE Healthcare, Uppsala, Sweden) according to the manufacturer’s manual. Equal amounts of protein lysates were separated by SDS-PAGE, on a Bio-Rad Criterion cell using Criterion precast 12.5% Tris-HCl gels (Bio-Rad). Immediately after the run, proteins were tank-blotted onto PVDF membranes. Transfer buffer contained 200 mM glycine, 25 mM Tris base, 0.1% SDS, 20% methanol. The post-transfer membranes were blocked with 5% dry milk in TBST and then incubated with the primary murine monoclonal antibody against Hsp72 (SPA-810, 1:2000, Stressgen/Assay Designs, Ann Arbor, MI, USA) or murine monoclonal anti-beta-Tubulin (# 691261, 1:2000, MP Biochemicals, Solon, OH, USA) for 16 hours at 4°C. After incubation with secondary, peroxidase-coupled antibody (Polyclonal Rabbit Anti-Mouse Ig’s/HRP P0260, 1:2000, Dako Cytomation, Carpinteria, CA, USA) bands were detected with an enhanced chemiluminescence detection system (ChemiDoc MP, Bio-Rad). 1D band densitometry (Bio-Rad ImageLab software) yielded background-subtracted values of optical density in the linear range for the observed expression levels.

The planned interims analysis revealed HSP expression to be quantifiable by 1D immunoblot under both PET conditions in only 4 of the 20 patients, secondary to peritoneal cell pellet lysate protein yields far lower than predicted in literature [32]. This outcome led to premature termination of patient recruitment and to the decision to analyze remaining portions of effluent cell protein samples by combining robust and highly sensitive saturation-labeling two-dimensional difference gel electrophoresis (2D-DIGE) with the specificity of 2-D Western blot, using saturation labeling (Saturn-2D Labeling Kit, NH DyeAGNOSTICS, Halle, Germany).

## Saturation labeling 2D-DIGE analysis

For saturation labeling 2D-DIGE, the Saturn-2D Labeling Kit (NH DyeAGNOSTICS, Halle, Germany) was used. Labeling, reduction and blocking reactions were carried out for 1 h in the dark at 37°C. Five μg of each protein sample was reduced with TCEP and labeled with S200 dye. Excess dye was removed by column chromatography. The internal pooled standard (IPS) containing pooled, equal amounts of all samples was similarly reduced and labeled in batch with S300 dye. Each S200-labeled sample was mixed with an equal amount of S300-labeled IPS to yield 10 μg total protein and subsequently separated in two dimensions by isoelectric focusing and horizontal large-format SDS-PAGE.

Following labeling, the mix was brought to a final volume of 450 μL with rehydration buffer (5 M urea, 0.5% CHAPS, 0.5% Pharmalyte and 12 μL/mL of DeStreak reagent (GE Healthcare)) and subsequently applied to one immobilized pH gradient (IPG) gel strip (ReadyStrip pH 3–10, non-linear, 24 cm, Bio-Rad). The loaded IPG strips were covered with silicone oil, actively rehydrated (50 V, 12 h, 20°C) and focused (Protean IEF, Bio-Rad) by increasing the voltage to 8000 V (total 84 kVh, current limit 30 μA/strip). Focused strips were stored at -80°C until further use. Before second dimension, each strip was incubated twice 15 min in 2 ml equilibration buffer (6 M urea, 2%(w/v) SDS, 25%(w/v) glycerol, and 3.3%(v/v) 50 mM Tris/HCl pH 8.8, stained with bromphenol blue) first supplemented with 20 mg dithiothreitol, then 96 mg iodoacetamide. The second dimension was carried out using precast horizontal non-fluorescent 12.5% polyacrylamide gels (255x200x0.65 mm, Serva, Heidelberg, Germany) on an HPE-FlatTop Tower (Serva) following the manufacturer’s manual. Gels were scanned immediately using a Typhoon Trio laser scanner (GE Healthcare) and an excitation wavelength of 555 nm and emission wavelength of 576 nm for the S200 dye (S200/Cy3 channel) and an excitation wavelength of 649 nm and emission wavelength of 664 nm for the S300 dye (S300/Cy5 channel). The photomultiplier voltage was chosen so that the most abundant protein spot was close to saturation. Sensitivity level was set to ‘normal’.

Aliquots of the IPS were analyzed by 2D immunoblot as previously described [36]. The total protein pattern of the IPS was imaged on the membrane by fluorescence detection, and was used to guide alignment of immunoblot signals and 2D-DIGE quantification with the aid of the image warping capabilities of the Delta2D 4.3 software (Decodon, Greifswald, Germany). Fluorescence values normalized to the internal standard were exported for all Hsp72 spots and subjected to statistical analysis.

## Intracellular glutamine concentration

Intracellular amino acid concentrations of PBMC after exposure to effluent mixed 1:1 with fresh PDF for 4 h were measured using 5x10^6^ cells. Following incubation cells were gently centrifuged (250 x g, 5 minutes, RT) and washed with PBS three times. To extract cells were incubated with PBS and ice-cold methanol 1:1 on dry-ice for 30 minutes. After thawing on ice for 10 minutes cells were centrifuged with (14 000. x g, 10 minutes, RT) and supernatants were collected. The extraction step was repeated once with 50% acetonitrile and supernatants were combined. Measurements of the concentrations of the 20 canonical amino acids were performed using the EZ:Faast amino acid analysis procedure as described above.

## Metabolomics analysis

Non-targeted metabolite profiling of peritoneal effluents from 8 randomly selected patients was performed with high-resolution mass spectrometry (HR/AM) by Q Exactive™ Plus Hybrid Quadrupole-Orbitrap™ in combination with a multi-dimensional Transcend UHPLC system with Allegro quaternary pumps (Thermo Fisher, San Jose, CA) [37].

Raw data were converted into an intensity matrix of masses determined with high precision by automatic chromatographic alignment of retention time, peak picking, and framing. Highly accurate measurement of apparent molecular weight allowed confinement of the set of putative identifications for *in-silico* fragmentation. In total, 200 masses were detected in PD effluents, using Sieve software 1.3 (Thermo Fisher). The Human Metabolome Database (HMDB) and the Kyoto Encyclopedia of Genes and Genomes (KEGG) Database were queried, resulting in 418 Chemspider IDs. 29 masses showed a significantly (p<0.05) different abundance at time point 4 h or were only detected either with or without AlaGln or differed in correlation coefficients between time points 0 and 4 h for > 0.3, and were detected in at least 5 of 8 patients or showed a qualitative difference, i.e. they were only detected either with or without AlaGln addition. Those 29 masses linking to 41 putative entities were forwarded to *in-silico* fragmentation (Mass Frontier). Resulting fragments were matched with those obtained in MS^2^ identification of chemical entities, as detailed in S1 Table.

## Cytokine levels and *ex-vivo* stimulated cytokine release

PDF effluent interleukin 6 (IL-6), interleukin 8 (IL-8), and tumor necrosis factor alpha (TNF-α) were measured by Immulite® system (Siemens, Vienna, Austria) or by Bio-Plex bead array (Bio-Rad, Hercules, CA). Lipopolysaccharide (LPS)-stimulated cytokine release was carried out as described [38, 39] with minor modifications. Human peripheral blood mononuclear cells (PBMCs) were exposed at 37°C either to 4 h PET effluents mixed 1:1 with fresh PDF or to pure effluents for 4 h. LPS (from *E*. *coli* 055:B5, Sigma-Aldrich, St. Louis, MO) was added at 0, 10 and 100 ng/ml to every PD effluent sample and incubated 37°C for 4 or 24 h. Supernatant TNF-α and IL-6 was analyzed by ELISA (eBioscience, San Diego, CA).

## Mouse model of PD-associated peritonitis

Mice (C57BL/6) were treated at the Medical University of Vienna according to a protocol based on published models [40–42] and approved by the ethics committee of the Medical University of Vienna and the Federal Ministry of Science, Research and Economy (GZ 66.009/00/0107-II/3b/2011). Mice underwent twice daily intraperitoneal injections of PDF (identical to that of the clinical trial) for 9 days, with addition to PDF on days 2 and 4 of 10^7^ cfu *Staphylococcus epidermidis*. PDF solutions contained (N = 10) or lacked (N = 10) 8 mM AlaGln. Body weight and pain status were assessed daily. Control mice (N = 4) received no injections. After 9 days, all mice were subjected to a 1 h PD dwell, then anesthetized and sacrificed. Biochemical measurements and cell counts were performed in blood and effluents, IL-6 and TNF-α levels were determined in effluent supernatants by ELISA (eBioscience). For functional measurements, aliquots of PDF effluent (400 μl) were *ex-vivo* stimulated with 10 ng/ml LPS for 4 h at 37°C, and supernatants were analyzed for IL-6 and TNF-α as above.

## Statistical analysis

Continuous variables are described by means and standard deviations (SD), or by median and range (minimum, maximum). Categorical variables are described with number of observations (N) and percentages (%). Carry-over effects were compared between the two sequence groups using independent samples t-tests applied to the sums across periods. No statistically significant difference was observed for any primary or secondary outcome (for HSP the ratio of carry-over effects amounts to 0.95 with 95% confidence interval 0.23 to 3.89, p = 0.940). For each outcome, a mixed model adjusted treatment effects for a potential period effect. Mean treatment effects estimated from models are presented with confidence intervals and *P*-values. Total Hsp72 expression was *a priori* determined as primary outcome. Therefore, the corresponding p-value is not adjusted for multiple testing. P-values for the remaining (secondary) outcomes are presented unadjusted, but significance after adjustment by the method of Bonferroni-Holm is indicated to allow a more conservative judgement for future studies. Potential differential subgroup effects were tested by according interaction terms. Two-sided p-values ≤0.05 were considered as statistically significant. Expected and observed numbers of enriched biological processes were compared using binomial tests. The animal experiment was analyzed using the Mann–Whitney U test. Computations (other than for metabolomics analysis) were performed with SAS 9.3 (SAS, Cary, NC) and graphed using R (http://www.r-project.org/).

## Adverse event monitoring

42 adverse events (AEs) were experienced during the study in 14 subjects (66.7% of the 21 patients in the ITT population). The study design included an observation time following the first treatment (4 weeks) markedly longer than the observation time after the second treatment (5 hours). Thus the majority of data reflect AEs observed after the first treatment (11 patients with AlaGln, 10 patients without AlaGln). One patient with AlaGln (with a severe AE due to overdosing with phenprocoumon during the washout-period) was admitted to the hospital and subsequently excluded from the trial after having experienced a total of six AE. None of the AEs (see S2 Table) was classified as related to the administration of AlaGln.

One AE (2.4%) was classified as severe (hyperanticoagulation due to overdosing with phenprocoumon). This event occurred three weeks after treatment with AlaGln, and was classified as unrelated to AlaGln.

14 AEs (33.3%) were classified as moderate. Seven events in four patients were related to increased blood pressure, with one subject experiencing four events. The remaining AEs included sporadic episodes of weakness, dizziness, hyperkalemia, anemia, loss of residual renal function and contamination of the PD catheter. Eight events occurred with and six without AlaGln. These AE were classified as not related to AlaGln.

27 AEs (64.3%) were classified as mild. These events included increased blood pressure (5), dizziness (4), pain at neck, shoulder or hip (3), cramps in the leg (2), procedural pain, i.e. too low temperature of infusion bag (2), and single episodes of gingivitis, gastrointestinal pain, vomiting, diarrhea, heartburn, common cold, influenza, water retention, hyperkalemia, menstrual pain and hypotension. Seventeen events occurred with and ten without AlaGln. Diarrhea persisted throughout both treatments. These AE were classified as not related to AlaGln.

## Results

### Patient clinical and laboratory data

21 patients were analyzed as an intention to treat (ITT) population (Table 1). No adverse events related to AlaGln administration were noted (see below). Laboratory parameters of blood and peritoneal dialysate from control PD and AlaGln groups were indistinguishable (Table 2).

**Table 2:** Clinical routine and safety parameters in blood and dialysate.

Endpoint^a^		Standard PDF	PDF with 8 mM AlaGln	Effect estimate	*P*-value
**Blood and serum parameters**					
	Erythrocytes (T/l)	3.7 (3.5–4.0)	3.6 (3.4–3.8)	0.1 (-0.1–0.3)	0.22
	Hemoglobin (g/dl)	11.1 (10.5–11.7)	10.7 (10.1–11.3)	0.3 (-0.3–1)	0.25
	Leucocytes (G/l)	7.4 (6.5–8.4)	7 (6–7.9)	0.5 (-0.2–1.1)	0.13
	Bicarbonate (mmol/l)	24.7 (23.6–25.9)	25.1 (23.9–26.2)	-0.3 (-1.3–0.6)	0.49
	pH (-)	7.34 (7.32–7.36)	7.35 (7.33–7.37)	-0.01 (-0.03–0.01)	0.32
	Alanine (mmol/l)	0.2 (0.2–0.3)	0.3 (0.3–0.3)	-0.1 (-0.1–0)	0.11
	Glutamine (mmol/l)	0.6 (0.5–0.6)	0.6 (0.5–0.7)	-0.1 (-0.2–0)	0.11
	Alanine aminotransferase (ALT)(U/L)	17.2 (14.4–20.0)	17.9 (15.1–20.6)	-0.7 (-3.3–1.9)	0.59
	Albumin (g/l)	35.9 (33.7–38.0)	35.3 (33.2–37.5)	0.5 (-0.6–1.7)	0.32
	Alkaline phosphatase (U/L) ^b^	119.3 (97.0–146.6)	117.2 (95.3–144.1)	1.0 (1.0–1.1)	0.49
	Aspartate aminotransferase (AST)(U/L)	16.6 (13.5–19.7)	17.7 (14.6–20.8)	-1.1 (-3.5–1.3)	0.35
	Bilirubin total (mg/dl)	0.4 (0.4–0.5)	0.4 (0.4–0.5)	0 (0–0)	0.21
	C-reactive protein (CRP)(mg/dl)	0.6 (0.4–0.9)	0.8 (0.5–1.1)	-0.2 (-0.4–0)	0.10
	Total calcium (mmol/l)	2.2 (2.1–2.3)	2.1 (2–2.3)	0 (0–0.1)	0.30
	Chloride (mmol/l)	98.3 (96.0–100.6)	98.2 (95.9–100.5)	0.1 (-0.8–1)	0.86
	Creatine Phosphokinase (U/L) ^b^	122.0 (85.9–173.4)	119.1 (83.8–169.2)	1 (0.9–1.2)	0.77
	Creatinine (mg/dl)	8.5 (7.0–10.1)	8.7 (7.1–10.2)	-0.1 (-0.5–0.2)	0.44
	Gammaglutamyltransferase (GGT)(U/L) ^b^	29.2 (19.6–43.4)	29.8 (20–44.3)	1 (0.9–1)	0.49
	Glucose (mg/dl)	111.8 (90.8–132.7)	113.3 (92.4–134.3)	-1.6 (-6.3–3.2)	0.49
	Lactic dehydrogenase (LDH)(U/L)	224.8 (197.3–252.3)	218.7 (191.2–246.2)	6.1 (-6.3–18.5)	0.31
	Phosphorus (mmol/l)	1.8 (1.5–2.1)	1.7 (1.4–2)	0.1 (-0.1–0.3)	0.20
	Potassium (mmol/l)	4.4 (4.2–4.6)	4.3 (4.1–4.6)	0.1 (-0.1–0.3)	0.40
	Sodium (mmol/l)	138.1 (136.9–139.3)	137.8 (136.6–139)	0.3 (-0.3–1)	0.31
	Total protein (g/l)	68.4 (65.2–71.6)	67.9 (64.7–71.1)	0.5 (-1–2.1)	0.48
	Urea nitrogen (BUN)(mg/dl)	52.9 (45.5–60.4)	51.8 (44.4–59.3)	1.1 (-4–6.2)	0.66
	Uric acid (mg/dl)	5.7 (5.2–6.1)	5.7 (5.3–6.2)	0 (-0.3–0.3)	0.85
**Dialysate parameters**					
	Ultrafiltration (ml)	-518.1 (-640.9 –-395.2)	-597.8 (-720.6 –-475)	79.7 (-32.6–192.1)	0.15
	Albumin loss (g)	0.88 (0.71–1.05)	0.93 (0.77–1.1)	-0.05 (-0.2–0.09)	0.46
	Total protein loss (g)	1.37 (1.10–1.64)	1.5 (1.24–1.77)	-0.13 (-0.37–0.11)	0.26
	Sodium dip (mmol/l) ^b^	4.0 (2.7–5.8)	4.2 (2.9–6.1)	0.9 (0.6–1.4)	0.77
	D_4 h_/D_0 h_ glucose	0.3 (0.2–0.3) ^c^	0.3 (0.2–0.3)	0 (0–0)	0.92
	D/P creatinine (4 h)	0.8 (0.7–0.8)	0.8 (0.7–0.8)	0 (0–0)	0.91
	D/P urea nitrogen (4 h)	0.96 (0.95–0.98)	0.96 (0.94–0.98)	0 (-0.02–0.02)	0.92
	D/P albumin (4 h)	0.01 (0.01–0.01)	0.01 (0.01–0.01)	0 (0–0)	0.55
	D/P total protein (4 h)	0.008 (0.006–0.010)	0.009 (0.007–0.011)	-0.001 (-0.002–0.001)	0.38

^a^ Data from clinical routine and safety parameters in blood and dialysate are presented as least squares means (95% confidence intervals) and mean difference (95% confidence intervals, P-value) estimated from the mixed models. P-values are given without adjustment for multiple testing, since none of the comparisons showed significant effects before adjustment.

^b^ Calculations performed on the log-scale; LS-means and confidence interval limits are back-transformed to the original scale; effect estimates give ratios (instead of differences).

^c^ Data for D_4h_/D_0_ glucose (N = 19) reflect a missing glucose concentration at time 0 h for the first patient. To evaluate the peritoneal permeability for small solutes, dialysate-to-plasma ratios (D/P) of creatinine, urea nitrogen, albumin and total protein were calculated from the 4 h effluent values (as the representative result) and the mean of the two plasma values (according to Twardowski *et al*. [43]). Creatinine measurements were corrected for the presence of glucose. The permeability for glucose was estimated as the ratio between the dialysate glucose concentrations at 4 h to that of 0 h dwell (D4/D0). As indirect measure of free water transport, the difference between dialysate sodium at 1 h and at baseline (sodium dip) was calculated

### Peritoneal solute transport and UF

AlaGln-containing PDF did not influence peritoneal transport of creatinine (D/P 4 h), BUN (D/P 4 h), glucose (D_4h_/D_0_) or proteins (D/P 4 h). Net UF during the peritoneal equilibration test (PET) and peritoneal fluid [Na^+^] difference between baseline and 1 h (sodium dip) were similarly unchanged by AlaGln (p>0.05; Table 2).

### Pharmacokinetics of AlaGln dipeptide and peritoneal Gln in PD-effluent

**Fig 2:**
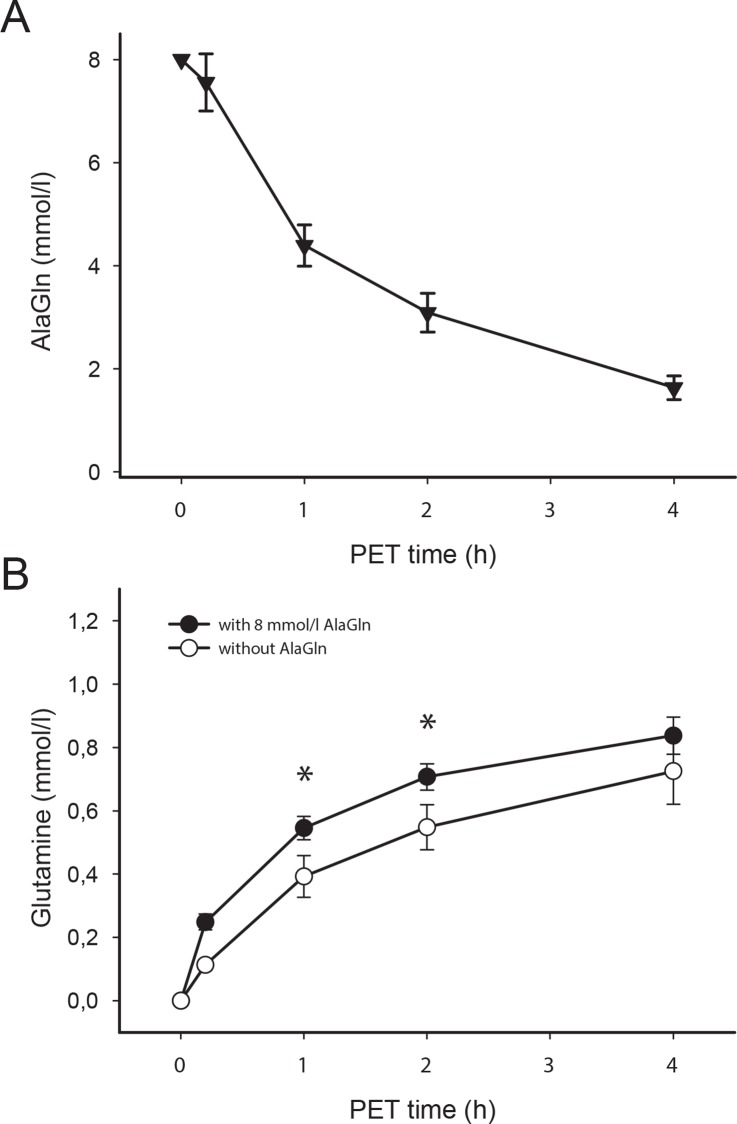
Temporal profile of dialysate glutamine and AlaGln dipeptide with standard PDF and with PDF containing 8 mM AlaGln. To assess transfer kinetics, AlaGln dipeptide and glutamine were analyzed in dialysate specimens sampled at baseline (immediately after completion of infusion), 1 h, 2 h and 4 h after initiation of the PET. As shown in panel A, peritoneal fluid AlaGln dipeptide concentration declined from 7.5 mmol/l immediately after infusion to < 2 mmol/l within four hours. The first-order elimination half-life of AlaGln dipeptide from the dialysate was 1.07 h. The value at time 0 h was set to 8 mM (as added to standard PDF). Panel B shows that glutamine concentrations in standard PDF remained below normal (serum) levels after 2 hours, but were significantly higher after 1 h and 2 h in the presence of added AlaGln. Data are shown as means and standard errors. Asterisk indicates p<0.05 *vs*. standard PDF (without AlaGln).

PET effluent [AlaGln] decreased progressively with parallel increase in peritoneal [Gln] over 2 h of the 4 h PET (Fig 2). Serum [Ala] and [Gln] remained unchanged (Table 2) and serum [AlaGln] was undetectable. Dialysate [Gln] increased faster in the presence than the absence of AlaGln (0.71 ± 0.19 *vs*. 0.55 ± 0.32 mmol/l at 2 h, p<0.05)

### HSP expression

**Fig 3:**
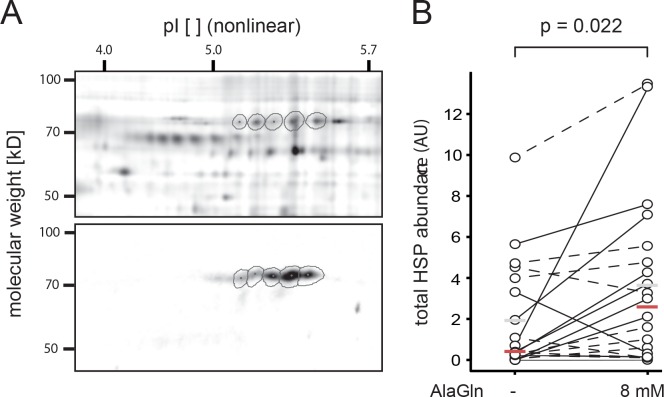
AlaGln treatment increased HSP expression in peritoneal effluent cells. Peritoneal cellular HSP expression in the study samples was detected by combining saturation labeling two-dimensional difference gel electrophoresis (2D-DIGE) with 2-D Western blotting. In panel A the upper segment shows the fluorescently labeled protein pattern whereas the lower segment shows the signals from immune-blotting, superimposed by the gel warping capabilities of Delta 2D software. The first dimension separation according to the isoelectric point (pI) was carried out on a nonlinear gradient. Panel B shows the quantification of total abundance of Hsp72 spot volumes, normalized by the internal standard. Total abundance of Hsp72 increased from 2.12 (CI 1.46–3.09) without AlaGln to 3.20 (CI 2.20–4.66) with AlaGln (effect size 1.51 (CI 1.07–2.14), p = 0.022) (N = 20 in each group, Counts: up = 14; down = 6). Grey bars indicate the mean; red bars indicate the median in each group.

Effluent cell HSP expression, the primary endpoint of this study, was higher following patient treatment with AlaGln. Total Hsp72 abundance (normalized spot volumes) increased from 2.12 without AlaGln (CI 1.46–3.09) to 3.20 with AlaGln (CI 2.20–4.66); effect size 1.51 (CI 1.07–2.14), p = 0.022). Fig 3 panel A shows 2D-DIGE detection of cellular Hsp72. Panel B summarizes Hsp72 changes for all patients.

### Peritoneal fluid metabolome

From 200 discrete masses identified by mass spectrometry in PD effluents under both conditions, 17 masses linking to 34 putative chemical entities changed significantly in abundance during the 4 h dwell (see S1 Table). Chemical entity identification on the basis of fragmentation and literature plausibility was possible for 13 masses corresponding to 10 metabolites (Table 3). AlaGln treatment was associated with increased metabolites of leucine/isoleucine, Gln and arginine, and increased metabolites related to unsaturated fatty acids and glycolipids, but to decreased metabolites of phenylalanine and tyrosine, homocysteic acid and nucleic acids.

**Table 3:** Metabolomic analysis of peritoneal effluents following treatment with standard PDF *vs*. PDF with 8 mM AlaGln.

Monoisotopic Mass (Da)	Chemspider ID	KEGG Compound ID	HMDB ID	Chemical Entity	Regulation^a^	Pathway Assignment^b^
131.09464	5880	C00123	HMDB00687	L-(+)-Leucine	up	amino acid metabolism
	6067	C00407	HMDB00172	L-(+)-Isoleucine	up	amino acid metabolism
**146.06914**	**5746**	**C00064**	**HMDB00641**	**L-Glutamine**	**up**	**amino acid metabolism**
164.04735	972	C00166	HMDB00205	Phenylpyruvic acid	down	amino acid metabolism
	553146	C01772,C12621,C00811	HMDB02641	o/m/p-Coumaric acid	down	amino acid metabolism
**164.08373**	**6745**	**C20327**	**HMDB00329**	**2-Phenylbutanoic acid**	**up**	**amino acid metabolism**
**181.07390**	**5833**	**C00082**	**HMDB00158**	**Tyrosine**	**up**	**amino acid metabolism**
**183.02014**	**154529**	**C16511**	**HMDB02205**	**(2S)-2-amino-4-sulfobutanoic acid**	**down**	**homocysteic acid**
**217.10600**	**571213**	**C15532**	**HMDB00856**	**N-Acetyl-L-Citrulline**	**up**	**amino acid metabolism**
**244.06953**	**5807**	**C00299**	**HMDB00296**	**Uridine**	**up**	**nucleic acid metabolism**
251.10184	13135	C00559	HMDB00101	2'-Deoxyadenosine	down	nucleic acid metabolism
	388325	C05198	HMDB01983	5'-deoxyadenosine	down	nucleic acid metabolism
**252.20892**	**13628094**		**HMDB00480**	**7,10-Hexadecadienoic acid**	**up**	**fatty acid and lipid metabolism**
**267.09674**	**163230**	**C00330**	**HMDB00085**	**Deoxyguanosine**	**down**	**nucleic acid metabolism**
**330.25589**	**4942831**	**C16513**	**HMDB01976, HMDB60113**	**Docosapentaenoic acid**	**up**	**fatty acid and lipid metabolism**
**358.30832**	**10381543**	**C01885**	**HMDB11131**	**1-Monoacylglycerol**	**up**	**fatty acid and lipid metabolism**

Metabolites with differential abundance in PD effluents after PET in the presence and absence of AlaGln. Monoisotopic masses, Chemspider IDs, KEGG compound IDs, HMDB IDs as queried and the (putative) chemical entities. Those 10 metabolites identified as unique chemical entities are in **bold**.

^a^ Up- and down-regulation were defined either by significant difference at t = 4 h or in the correlation with time or as qualitative difference with *vs*. without AlaGln (see Methods for details).

^b^ Pathway assignment used the HMDB and KEGG databases and searches by PubMed and GoogleScholar.

### Peritoneal cell count

PET effluent cell numbers and differentials varied widely among patients, from <1 to >100 peritoneal cells/μl (Table 4). CD45-positive leukocytes were 10.5 cells/μl (CI 8.9–12.2) without AlaGln *vs*. 9.4 cells/μl (CI 8.1–10.8) in its presence. Differential cell counts were also unchanged by AlaGln.

**Table 4:** Secondary outcome parameters in dialysate effluent at 4 hours.

Endpoint^a^		Standard PDF	PDF with 8 mM AlaGln	Effect estimate	*P*-value
AlaGln (mmol/l)^b^		n/a	1.6 ± 1.0 (0.3–3.7)	–	–
Glutamine (mmol/l)		0.7 (CI 0.5–0.9)	0.8 (CI 0.7–1.0)	-0.1 (CI -0.4–0.1)	0.36
CA-125 (U/ml)		27.2 (CI 20.8–33.5)	27.0 (CI 20.7–33.4)	0.1 (CI -2.9–3.1)	0.92
IL-6 (pg/ml)		139.3 (CI 83.8–194.7)^c^	117.7 (CI 62.9–172.6)	21.5 (CI -16.9–59.9)	0.25
IL-8 (pg/ml)		3.8 (CI 2.9–4.8)	4.1 (CI 3.1–5.0)	-0.3 (CI -1.5–1.0)	0.65
TNF-α (pg/ml)		2.9 (CI 2.3–3.6)	2.8 (CI 2.2–3.5)	0.1 (CI -0.7–0.8)	0.85
Cell count (cells/μl)		13.6 (CI 4.5–22.6)	11.6 (CI 2.6–20.6)	2.0 (CI -9.9–13.8)	0.73
Cytokeratin-positive (cells/μl)		3.1 (1.4–4.7)	2.2 (0.8–3.5)	0.1 (-0.2–0.4)	0.60
CD45-positive (cells/μl)		10.5 (8.9–12.2)	9.4 (8.1–10.8)	-0.1 (-0.4–0.2)	0.60
	Macrophages/ Monocytes (%)	87.7 (81.1–94.3)	88.1 (81.4–94.7)	-0.3 (-9.6–8.9)	0.94
	Lymphocytes (%)	7.3 (1.4–13.1)	4.0 (-1.8–9.9)	3.2 (-5.1–11.5)	0.42
	Neutrophils (%)	3.4 (0.9–5.9)	6.3 (3.8–8.7)	-2.7 (-5.6–0.3)	0.60
	Eosinophils (%) ^d^	1.6 (0.1–3.1)	1.7 (0.2–3.3)	-0.2 (-2.3–2.0)	0.88

^a^ Results from 4 h PET effluent presented as least squares means (95% confidence intervals) and mean difference (95% confidence intervals, *P*-value) estimated from the mixed models. P-values for secondary outcomes are given without adjustment for testing multiple outcomes, since none of the comparisons showed significant effects before adjustment.

^b^ Data for AlaGln are represented as means ± standard deviations with the minimum and maximum values (range) in parentheses.

^c^ This IL-6 value was calculated after excluding one extremely high value in the standard PDF group.

^d^ Basophil count was zero in all samples and was therefore omitted from the table.

### Peritoneal biomarkers of cell mass, inflammation and immunocompetence

**Fig 4:**
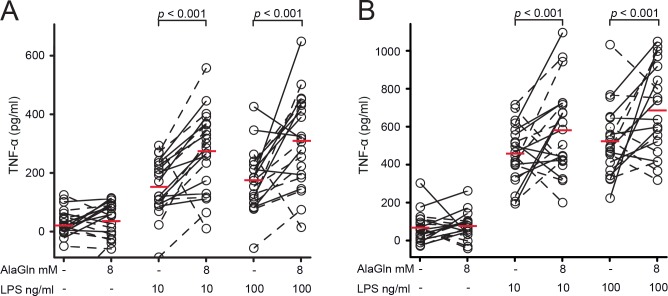
Lipopolysaccharide (LPS)-stimulated release of tumor necrosis factor alpha (TNF-α) by heterologous normal human peripheral blood mononuclear cells (PBMC) following a 4 h *ex-vivo* exposure to PD effluents obtained from the PET of patients treated with standard PDF or AlaGln-supplemented PDF. In panel A exposure to effluent mixed 1:1 with fresh PDF (representing the situation of the “early dwell”) is shown. In panel B exposure to pure effluent (representing the situation of the “late dwell”) is shown. Each data point represents the mean value of TNF-α release by PBMC from 4 healthy donors exposed to each PET effluent (n = 20 in each group). The left part of the figures represents the control with exposure to patient effluents without stimulation by LPS (0 ng/ml). Differences between the presence and absence of AlaGln were statistically significant for PBMC in the presence of 10 or 100 ng/ml LPS in case of the early and the late dwell (p<0.001). (Panel A counts: 10 ng/ml: down = 6, up = 14; 100 ng/ml: down = 7, up = 13; Panel B counts: 10 ng/ml: down = 1, up = 19; 100 ng/ml: down = 4, up = 16). Red bars indicate the mean in each group.

**Fig 5:**
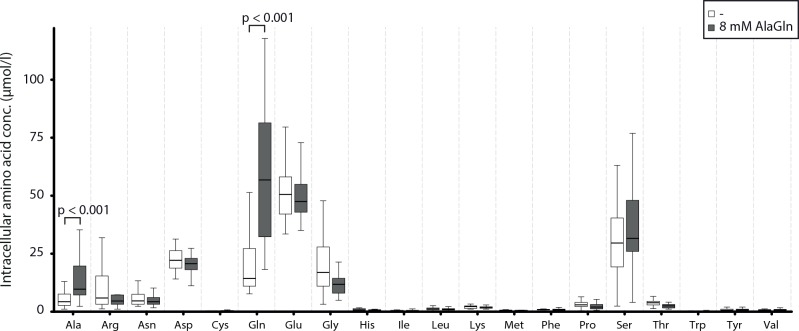
Intracellular glutamine of peripheral blood mononuclear cells (PBMC) following exposure to PD effluents obtained from the PET of patients treated with standard PDF or AlaGln-supplemented PDF. Intracellular amino acid concentrations of PBMC after exposure to effluent mixed 1:1 with fresh PDF for 4 h (*i*.*e*. at the start time of LPS stimulation (see Fig 4, panel A) are shown. Of the 20 canonical amino acids, only alanine and glutamine show significantly higher intracellular concentrations when PBMC are exposed to effluents from patients treated with AlaGln-supplemented PDF (p<0.001, n = 80 in each box (= 4 biological PBMC experiments exposed to 20 PD effluents).

PDF levels of cancer antigen 125 (CA-125), IL-6, IL-8 and TNF-α were indistinguishable between treatments with and without AlaGln, (Table 4); p>0.05). LPS exposure (10 or 100 ng/ml) increased PBMC release of TNF-α by 121.0 pg/ml (CI 67.8–174.1; p<0.001) or by 134.0 pg/ml (CI 81.2–186.8; p<0.001), respectively when incubated with effluent mixed with unused PDF (Fig 4, panel A) and by 135.0 pg/ml (CI 49.7–220.4; p = 0.002) or by 151.3 pg/ml (CI 65.3–237.2; p<0.001), respectively when incubated with pure effluent (Fig 4, panel B). The increment attributable to AlaGln treatment was 89.4 pg/ml (CI 58.6–120.3, p<0.001, 1:1 mix) and 94.8 pg/ml (CI 46.0–143.6, p<0.001, pure). LPS exposure (10 or 100 ng/ml) increased PBMC release of IL-6 by 541.5 pg/ml (CI 210.1–872.9; p = 0.0014) or by 557.2 pg/ml (CI 222.2–892.1; p = 0.0012), respectively when incubated with pure effluent (S1 Fig). The increment attributable to AlaGln treatment was 368.1 pg/ml (CI 175.3–561.0 p<0.001). Intracellular levels of glutamine and alanine increased significantly (Fig 5).

**Fig 6:**
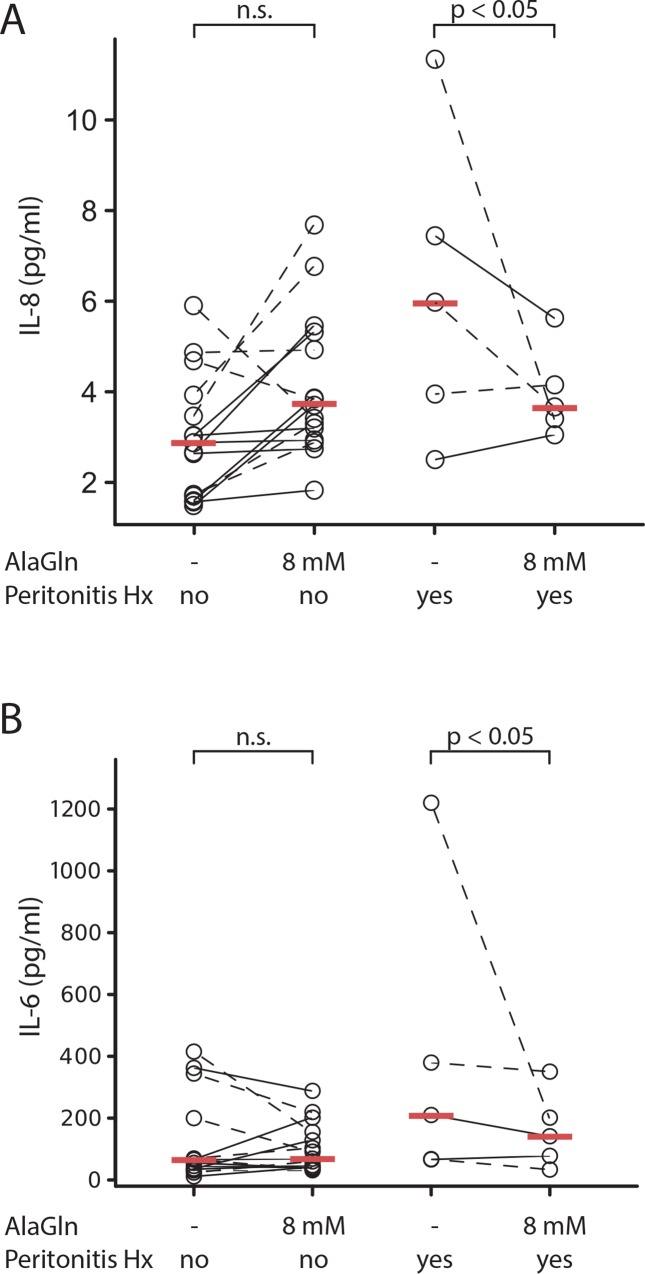
Levels of interleukin 8 (IL-8) and interleukin 6 (IL-6) in PET effluents from patients treated with PDF in the absence or presence of 8mM AlaGln. Each data point represents the IL-8 (A) or IL-6 (B) concentration in PD effluent of a single study patient treated with standard PDF lacking or containing added AlaGln. Among patients with history of peritonitis (Peritonitis Hx), IL-8 levels after exposure to PDF containing AlaGln were significantly lower than after exposure to standard PDF (p<0.05). History of peritonitis was not associated with differences in IL-6 levels (p>0.05). Statistical mixed model analysis for IL-6 was performed after excluding the single extremely high value in the standard PDF group. Red bars indicate the median in each group.

Sub-group analyses of biomarkers of inflammation (unplanned in the original protocol) compared patients with and without previous history of peritonitis. AlaGln treatment decreased IL-8 levels by 2.2 pg/ml (CI 0.1 to 4.3, p<0.05) in the peritonitis-positive subgroup, in contrast to a non-significant increase of 1.1 pg/ml (CI -0.1 to 2.3) in patients without peritonitis (Fig 6). Three of five patients with a history of peritonitis and with above-average levels of effluent IL-8 (indicating ongoing peritoneal inflammation) exhibited a decline following 4 h of AlaGln exposure, whereas the 2 other patients did not. In contrast, peritonitis history was statistically unassociated with IL-6 levels (p>0.05).

### Inflammation and immuno-competence in a mouse model of PD-related peritonitis

**Fig 7:**
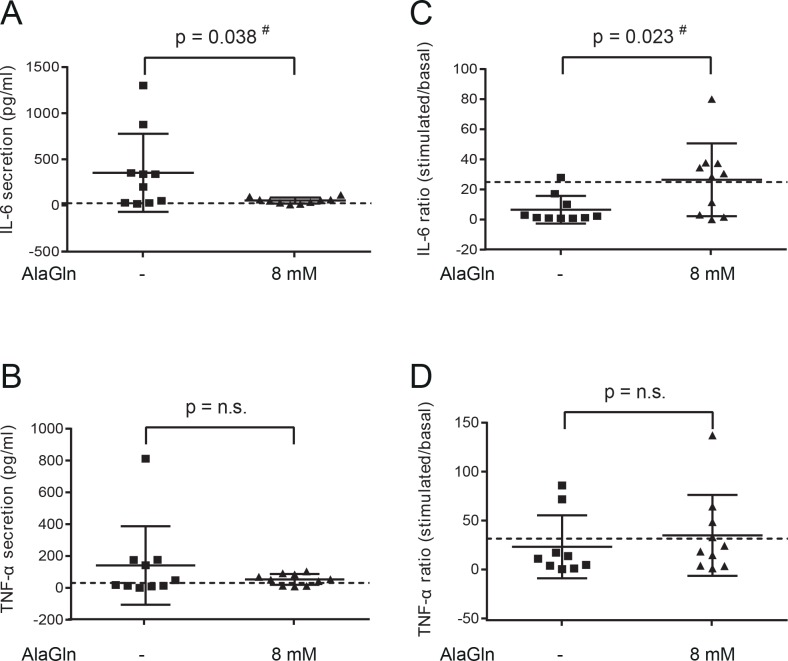
Inflammation and immuno-competence in the mouse model of PD- associated peritonitis. Mice were subjected for 9 days to twice-daily intraperitoneal PDF injection, in combination with 10^7^ cfu *Staphylococcus epidermidis* on days 2 and 4. PDF was applied with (N = 10) or without (N = 10) added 8 mM AlaGln. Control mice (N = 4) received no treatment. As shown in Panel A and B, basal levels of IL-6 and TNF-α in peritoneal effluents were lower in the group treated with AlaGln than in the group treated without it, although not statistically significant, when p-values were Bonferroni-corrected for testing two outcomes. Values of controls are shown as dashed line. *Ex-vivo* LPS stimulation (10 ng/ml; 4 h) of peritoneal cells resulted in increased IL-6 and TNF-α release in control animals (ratio of stimulated/unstimulated is shown as dashed line C and D). *Ex-vivo* stimulated release of IL-6 and TNF-α was depressed in the group without AlaGln and restored in the group with AlaGln (raw p = 0.023 *vs*. without AlaGln for IL-6; Bonferroni-corrected p = 0.046). Data are shown as individual points (each representing an animal), means and standard errors. # P-values in the figure are given unadjusted for multiple testing.

AlaGln group peritoneal IL-6 levels were 53.3 pg/ml *vs*. 353.0 pg/ml without AlaGln (raw p = 0.038). AlaGln group TNF-α levels were 53.4 pg/ml *vs*. 141.1 pg/ml without AlaGln (p = n.s.) (see S2 Fig) and Table 5. *Ex-vivo* LPS stimulation of control mouse peritoneal cells increased IL-6 release 25-fold and TNF-α release 40-fold. *Ex-vivo* LPS-stimulated release of IL-6 and TNF-α were lower without AlaGln (respective increases of 3 and 10 fold) and restored in the AlaGln-treated group (30 and 25 fold increases, respectively; raw p = 0.023; Bonferroni-corrected p = 0.046 for IL-6), suggesting improved cellular immuno-competence (Fig 7).

**Table 5:** Effluent and blood parameters of the mouse model of PD-related peritonitis.

Parameter		Control	Standard PDF	PDF with 8 mM AlaGln	*P*-value^a^
***Effluent***					
	WBC/μl (x10^4^)	1.58 (1.5–1.9)	1.28 (0.1–11.3)	0.83 (0.3–2.2)	0.95
	Macrophages/Monocytes (%)	98 (97.3–98.8)	57 (0–81.5)^#^	62 (37.5–78.8)^#^	0.78
	Neutrophils (%)	2 (1.3–2.8)	4 (0–28.5)	24.5 (6–39.5)	0.24
	Eosinophils (%)	0 (0–0)	0 (0–0)	0 (0–0)	-
***Blood***					
	Mononuclear cells (%)	81.5 (65.8–88.3)	70 (53.3–91.3)^#^	73 (65–89.3)^#^	0.87
	Segmented neutrophils (%)	15 (10.3–29.5)	25 (7–39.5)	22.5 (9.3–27.5)	0.87
	Banded neutrophils (%)	0.5 (0–1.8)	0.5 (0–2.5)^#^	0 (0–1.3)	0.10
	Eosinophils (%)	3 (0.5–4)	2.5 (1.8–4.3)	4 (2.5–6)	0.99

Data are presented as median an interquartile range in parentheses. As the data where not normally distributed, groups were compared using the Mann-Whitney U test.

^a^ P-values are given for comparisons of the Standard PDF group versus the PDF with added 8 mM AlaGln group.

# p<0.05 versus control group. Basophil count was zero in all samples and was therefore omitted from the table.

## Discussion

This first-in-man study demonstrates that AlaGln addition to standard glucose-based PDF restores or maintains several important CSR indices in peritoneal cells during a single 4 hour dwell in PD patients undergoing a peritoneal equilibration test. These data support and extend our previous studies in experimental models of PD which have demonstrated that supplementation of glucose-based PDF with pharmacological doses of AlaGln protected mesothelial cells *in-vitro* and preserved peritoneal integrity *in-vivo* [25, 31]. The dipeptide AlaGln has long been a component of enteral and parenteral nutrition formulations used in critically ill patients [44]. The results from this current trial suggest that these cytoprotective effects of AlaGln may be translated into the clinical setting of PD.

This randomized, controlled cross-over trial was performed in a representative PD population, each of whom underwent 2 single 4 hour administrations of standard PDF with and without added AlaGln. Peritoneal Gln levels in patients without AlaGln treatment demonstrated extracellular levels previously associated with increased cell vulnerability and inadequate CSR, and remained below normal serum levels for most of the standard PDF dwell period, with trans-peritoneal Gln transport rates comparable to that of creatinine [45, 46]. These findings, along with previous reports of impaired metabolic and immuno-competence status [25, 47–49], suggest that standard PD may impose on peritoneal cells a clinically relevant degree of Gln starvation. The present study showed that PDF supplementation with AlaGln rapidly restored peritoneal Gln concentrations to levels that may better counteract some aspects of PD pathology [45, 46].

Peritoneal cell expression of Hsp72, one of the best described of cytoprotective human stress proteins, was the primary outcome parameter in this trial. AlaGln addition significantly increased peritoneal cell Hsp72 expression, confirming our previous findings of Gln effects on mesothelial cell CSR in *in-vitro* and *in-vivo* PD models [25, 31, 50]. In conditions of low plasma [Gln] such as critical illness and sepsis, Gln-starved macrophages express inadequate Hsp72 levels with reduced Hsp72 mRNA half-life [48]. We recently demonstrated increased post-translational *O*–GlcNAc protein modification in cultured mesothelial cells upon AlaGln addition to PDF, associated with elevated Hsp72 expression and improved resistance against PDF toxicity [50]. Pharmacological levels of AlaGln, as used in this study, increase HSP expression in various cell types via post-translational modification of its key regulator HSF-1, and thereby improve outcome in experimental and clinical settings [44, 51].

In the current study, analysis of metabolomics data was used to search for additional AlaGln effects associated with or beyond those of CSR. In the untargeted metabolomics approach [37] increased peritoneal levels of the arginine precursor *N*-acetyl-L-citrulline agree with recently reported effects of Gln administration on macrophage arginine production [28]. Restoration of systemic arginine levels correlated with improved clinical outcome [52]. Effects on tyrosine and homocysteic acid levels similar to those found with peritoneal AlaGln administration were reported with stabilization of immune-competent cells and reduction of reactive oxygen species [53]. The increased abundance of unsaturated fatty acids and glycerolipids might reflect scavenging of reactive substances in the PDF. Such antioxidative effects of AlaGln might also explain reduced deoxyadenosine and deoxyguanosine levels, the latter a risk marker for PD technical failure [54]. Taken together, these metabolomics findings demonstrate clear supporting evidence for Gln associated reprogramming consistent with restored cytoprotective CSR in the dialyzed peritoneal cavity.

Functionally, exposure to AlaGln-supplemented PDF significantly enhanced *ex-vivo* release of TNF-α and IL-6 upon LPS-stimulation, a biomarker which has previously been adapted to discriminate between effects of different PDF on peritoneal host defenses [9, 10, 38, 39]. Our trial confirmed marked suppression of LPS-stimulated TNF-α and IL-6 release by a standard PDF and demonstrated comparable effects of addition of AlaGln to PDF as were previously described by improving biocompatibility of PDF [9, 10, 38, 39]. Using various systemic biomarkers, smoldering inflammation [55] and Gln deficiency were shown to suppress immune competence during sepsis or following major trauma or surgery [44, 56, 57]. In a meta-analysis, AlaGln addition to parenteral nutrition (at amounts similar to those added here to PDF) reduced infection rate and shortened hospital stay in critically ill patients [30]. In these patients, parenteral AlaGln restored *ex-vivo*-stimulated cytokine release from whole blood [58]. AlaGln addition to PDF may, therefore, also be translated to PD patients to overcome reduced peritoneal host defense.

AlaGln treatment did not change PDF effluent cell counts. In the acute rat PD model, Gln addition to PDF increased HSP expression and decreased mesothelial cell detachment [31]. Acute PDF exposure in that model, however, was performed in a virgin peritoneal cavity (resulting in severe cell detachment) whereas the single PDF exposure in the current trial was performed in stable patients after several months' experience of PD. Prior repeated PDF exposure in our study subjects may have produced a steady-state of peritoneal stress and repair processes minimally perturbed by the single dwell studied here. Low cell numbers and variability in PDF cell counts precluded conclusions about protective effect of AlaGln on mesothelial cell detachment. Interspecies differences between rat and human might play an additional role.

Other surrogate biomarkers reflecting peritoneal membrane and immune status, such as CA-125, TNF-α, IL-6 and IL-8, also showed no effects of AlaGln in this unselected, standard-risk PD population, likely reflecting the limitation of a single PDF treatment. Indeed, several weeks' treatment with biocompatible PDF is required before PD CA-125 levels differ significantly from those of standard PDF [59]. An ongoing randomized clinical cross over trial comparing 8 weeks' treatment with standard versus AlaGln-supplemented PDF (https://www.clinicaltrialsregister.eu/ctr-search/trial/2013-000400-42/AT) currently investigates the ability of AlaGln for peritoneal immunomodulation and membrane protection, and so further tests the hypothesis raised in this study in a larger PD population following more extended treatment.

Comparable to reported prevalence in similar PD populations, 25% of patients in this trial had prior history of peritonitis [5, 60]. Chronic post-infectious peritoneal inflammation may define a subgroup susceptible to peritoneal scarring and PD technical failure [61–63]. The biomarker data demonstrate significant AlaGln effects on peritoneal IL-8 levels in patients with a history of peritonitis and with above-average levels of effluent IL-8 (indicating ongoing peritoneal inflammation), likely reflecting attenuated sterile peritoneal inflammation [64, 65]. Although AlaGln addition to PDF may thus represent a promising approach to interrupt the vicious cycle of injury-induced peritoneal inflammation in these high-risk patients, this suggestion is merely hypothesis- generating at this point, rather than proving a general anti-inflammatory action [64, 65]. Like all human PD trials our study suffers from the limitation of mere inter-group comparison while lacking controls. In order to test the hypothesis of immuno-modulatory effects of AlaGln we performed a sub-acute mouse PD model of combined peritoneal stress by infectious and cytotoxic stimuli. Complementing the clinical trial data, the mouse model showed that AlaGln addition to PDF attenuated peritoneal inflammation markers close to control levels and restored *ex-vivo*-stimulated cytokine release. Bioinformatic analysis of the PDF-stressed peritoneal cell proteome has previously identified simultaneous activation of cellular inflammation and repair by bacterial components and cytotoxic stimuli [14, 21, 26]. Intracellular cross-talk may sensitize cells to subsequent insults or, alternatively, sensing of cellular injury may induce sterile inflammation [66, 67]. AlaGln may exert immune-modulatory effects through several potential anti-inflammatory pathways, akin to the specific inhibition of TLR4 expression by Gln [68]. A recent study in chronic uremic rat and mouse PD exposure models confirmed that AlaGln related immuno-modulation may reduce peritoneal damage [69]. Inflammatory injury may be attenuated by Gln treatment-associated upregulation and stabilization of key signal regulators and/or effectors, potentially mediated by *O*-GlcNAcylation [70]. Future work using high-resolution LC-MS-based characterization of individual post-translational protein modifications will further elucidate this potential mode of AlaGln action in peritoneal cells of PD patients.

In conclusion, this study demonstrates that a single dwell of AlaGln-supplemented glucose-based PDF increased peritoneal cell HSP expression and enhanced stimulated *ex-vivo* cytokine release. AlaGln attenuated peritoneal effluent inflammatory markers in patients with prior peritonitis (and in a mouse model following exposure to infected PDF). These data emphasize the potential of this novel therapeutic approach to improve peritoneal health by counteracting PD-related pathomechanisms by restoring adequate cellular stress responses. The study provides the basis for an ongoing phase II trial testing the clinical role of peritoneal AlaGln as a beneficial pharmacologic intervention in routine clinical PD.

## Supporting Information

S1 CONSORT Checklist (PDF)

S1 Fig Lipopolysaccharide (LPS)-stimulated release of interleukin 6 (IL-6) by heterologous normal human peripheral blood mononuclear cells (PBMC) following a 4 h ex-vivo exposure to PD effluents obtained from the PET of patients treated with standard PDF or AlaGln-supplemented PDF. Exposure to pure effluent is shown. Each data point represents the mean value of IL-6 release by PBMC from 4 healthy donors exposed to each PET effluent (n = 20 in each group). The left part of the figure represents the control with exposure to patient effluents without stimulation by LPS (0 ng/ml). Differences between the presence and absence of AlaGln were statistically significant for PBMC in the presence of 10 or 100 ng/ml LPS (p<0.001). Red bars indicate the median, grey bars the mean in each group.
(PDF)

S2 Fig Mouse cytokine *vs*. cell count scatter. Peritoneal effluent cytokine levels were related to peritoneal leucocytes in mice treated for 9 days with PD, in combination with 10^7^ cfu *Staphylococcus epidermidis* on days 2 and 4. Treatment with PDF with AlaGln (N = 10) resulted in lower basal IL-6 levels (panel A) for given leucocyte counts than treatment with PDF without AlaGln (N = 10). This effect was not observed for TNF-α (panel B). Control mice (N = 4) demonstrated less scattered values with levels were comparable to those animals that received treatment with PDF with AlaGln.
(PDF)

S1 Study Protocol (PDF)

S1 Table Putative chemical entities changed significantly in abundance following treatment with standard PDF *vs*. PDF with 8 mM AlaGln. Monoisotopic masses, calculated and observed, their Chemspider IDs, HMDB IDs and KEGG IDs as queried from the databases and the (putative) chemical entities. ^a^ Up-and down-regulation was defined either as significant difference at time point 4 h, or in the correlation with time, or as qualitative difference with *vs*. without AlaGln (see Methods for details).
(PDF)

S2 Table Summary of adverse events. AE = Adverse Event; Calculations are based on the total number of AEs (N = 42). One mild AE (mild diarrhea) could not be assigned to treatment A or B since it persisted during both study phases.
(PDF)
