# Supplementary material for: Addition of Alanyl-Glutamine to Dialysis Fluid Restores Peritoneal Cellular Stress Responses – A First-In-Man Trial
Source: PLoS One. 2016 Oct 21;11(10):e0165045. doi: 10.1371/journal.pone.0165045 (PMC5074513; doi:10.1371/journal.pone.0165045)
Supplement: S1 Fig — Exposure to pure effluent is shown. Each data point represents the mean value of IL-6 release by PBMC from 4 healthy donors exposed to each PET effluent (n = 20 in each group). The left part of the figure represents the control with exposure to patient effluents without stimulation by LPS (0 ng/ml). Differences between the presence and absence of AlaGln were statistically significant for PBMC in the presence of 10 or 100 ng/ml LPS (p<0.001). Red bars indicate the median, grey bars the mean in each group. (PDF) [file pone.0165045.s002.pdf]

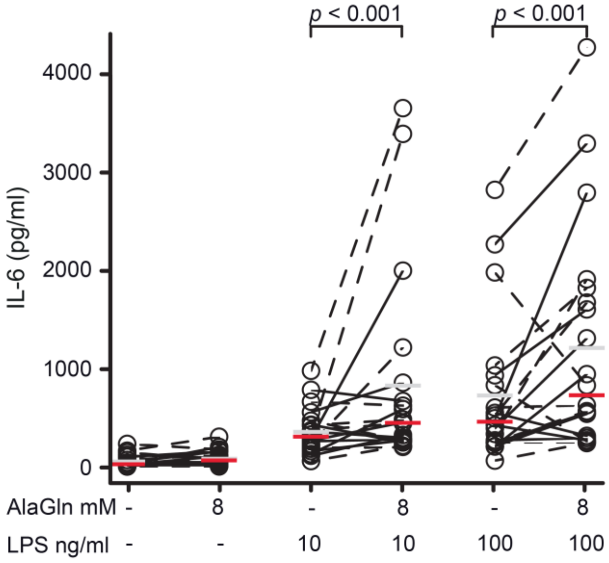


**S1 Fig. Lipopolysaccharide (LPS)-stimulated release of interleukin 6 (IL-6) by heterologous normal human peripheral blood mononuclear cells (PBMC) following a 4 h ex-vivo exposure to PD effluents obtained from the PET of patients treated with standard PDF or AlaGln-supplemented PDF.** Exposure to pure effluent is shown. Each data point represents the mean value of IL-6 release by PBMC from 4 healthy donors exposed to each PET effluent (n=20 in each group). The left part of the figure represents the control with exposure to patient effluents without stimulation by LPS (0 ng/ml). Differences between the presence and absence of AlaGln were statistically significant for PBMC in the presence of 10 or 100 ng/ml LPS ($p<0.001$). Red bars indicate the median, grey bars the mean in each group.
