# Supplementary material for: Addition of Alanyl-Glutamine to Dialysis Fluid Restores Peritoneal Cellular Stress Responses – A First-In-Man Trial
Source: PLoS One. 2016 Oct 21;11(10):e0165045. doi: 10.1371/journal.pone.0165045 (PMC5074513; doi:10.1371/journal.pone.0165045)
Supplement: S2 Fig — Peritoneal effluent cytokine levels were related to peritoneal leucocytes in mice treated for 9 days with PD, in combination with 107 cfu Staphylococcus epidermidis on days 2 and 4. Treatment with PDF with AlaGln (N = 10) resulted in lower basal IL-6 levels (panel A) for given leucocyte counts than treatment with PDF without AlaGln (N = 10). This effect was not observed for TNF-α (panel B). Control mice (N = 4) demonstrated less scattered values with levels were comparable to those animals that received treatment with PDF with AlaGln. (PDF) [file pone.0165045.s003.pdf]

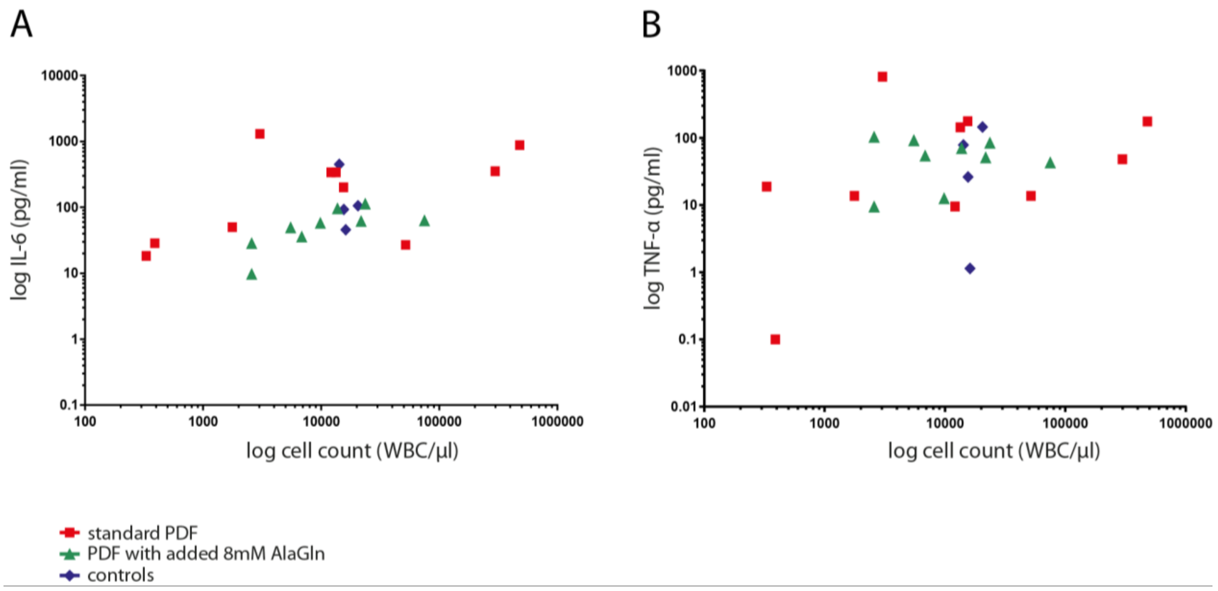

S2 Fig. Mouse cytokine *vs.* cell count scatter. Peritoneal effluent cytokine levels were related to peritoneal leucocytes in mice treated for 9 days with PD, in combination with 10^7 cfu *Staphylococcus epidermidis* on days 2 and 4. Treatment with PDF with AlaGln (N=10) resulted in lower basal IL-6 levels (panel A) for given leucocyte counts than treatment with PDF without AlaGln (N=10). This effect was not observed for TNF-α (panel B). Control mice (N=4) demonstrated less scattered values with levels comparable to those of animals that received treatment with PDF with AlaGln.
