# Supplementary material for: Addition of Alanyl-Glutamine to Dialysis Fluid Restores Peritoneal Cellular Stress Responses – A First-In-Man Trial
Source: PLoS One. 2016 Oct 21;11(10):e0165045. doi: 10.1371/journal.pone.0165045 (PMC5074513; doi:10.1371/journal.pone.0165045)
Supplement: S1 Study Protocol — (PDF) [file pone.0165045.s004.pdf]

ETHIK-KOMMISSION
DER MEDIZINISCHEN UNIVERSITÄT WIEN
Borschkegasse 8b/6 - A-1090 Wien, Austria
☎ 0043 1 404 00 – 2147, 2244 & 🖹 0043 1 404 00 – 1690
E-Mail: ethik-kom@meduniwien.ac.at
ethikkommission.meduniwien.ac.at

**AUSZUG AUS DEM PROTOKOLL DER AMENDMENTS U.DIV.MELDUNGEN
DER ETHIK-KOMMISSION
VOM JUNI 2011**

006/06/2011) EK Nr. 867/2010 Eudract-Nr:. 2010-022804-29

Univ.Prof.Dr. Christoph Aufricht(1), Univ.Prof.Dr. Andreas Vychytil(2) ,
Sponsor/CRO: Koordinierungszentrum für Klinische Studien
Univ.Klin.f. Kinder- u. Jugendheilkunde(1), Univ.Klin.f. Innere Medizin III, Klin.Abt.f. Nephrologie u. Dialyse(2)
An Open Label, Randomized, Two-Period Crossover Study to Evaluate the Safety and Efficacy of the Addition of Alanyl-Glutamine-Dipeptide to Dialysis Solutions in Peritoneal Dialysis (PD)

Nachmeldung eines CRO (Koordinierungszentrum für Klinische Studien, MUW)

Protokoll Vers. 1.3 vom 30.05.2011
CRF Vers. 1.5 vom 31.05.2011
Letter of Authorization vom 06.06.2011
Signature pages

(Meldung 01.06. , 06.06.2011)

Die Kommission nimmt diese Meldung ohne Einspruch zur Kenntnis.

Univ.Prof.Dr. Ernst Singer
Vorsitzender der Kommission

07. Juni 2011

MEDIZINISCHE UNIVERSITÄT WIEN

Clinical Study Protocol

An Open Label, Randomized, Two-Period Crossover Study to Evaluate the Safety and Efficacy of the Addition of Alanyl-Glutamine-Dipeptide to Dialysis Solution in Peritoneal Dialysis (PD)

(Ala-Gln in PD)

Version: 1.3 vom 30.5.2011

Confidentiality Statement

The information contained in this document, especially unpublished data, is the property of the sponsor of this study, Department of Pediatrics and Adolescent Medicine, Clinical Division of Nephrology and Gastroenterology, Medical University of Vienna. It is therefore provided to you in confidence as an investigator, potential investigator, or consultant, for review by you, your staff, and an Independent Ethics Committee or Institutional Review Board. It is understood that this information will not be disclosed to others without written authorization from the Department of Pediatrics and Adolescent Medicine, Clinical Division of Nephrology and Gastroenterology, Medical University of Vienna, except to the extent necessary to obtain informed consent from those persons to whom the study drug may be administered.

**Test drug (IMP)**	Dipeptiven [N(2)-L-Alanyl-L-Glutamine]<br>Produced by Fresenius Kabi Austria GmbH, Graz
**Protocol authors**	Ao. Univ. Prof. Dr. Andreas Vychytil,<br>Ao. Univ. Prof. Dr. Christoph Aufricht, Mag. (FH) Astrid Scherr, Dr. Magdalena Pilz, Mag. Ortrun Neuper
**Academic investiga-tor**	Ao. Univ. Prof. Dr. Andreas Vychytil,<br>Department of Internal Medicine III, Clinical Division of<br>Nephrology and Dialysis
**Document type**	Clinical study protocol
**Study phase**	Phase I-II
**Document status**	Protocol version 1.3
**Date**	30. May.2011
**Number of pages**	52

SPONSOR, INVESTIGATOR, MONITOR AND SIGNATURES

**Clinical (Prinicipal) investigator (AMG §§ 2a, 35,36)**	Ao.Univ.Prof.Dr. Andreas Vychytil,<br><br>Department of Internal Medicine III, Clinical Division of Nephrology and Dialysis , Medical University of Vienna, Austria
**Sponsor**<br><br>**(AMG §§ 2a, 35,36)**	Medical University of Vienna<br><br>Department of Pediatrics and Adolescent Medicine, Clinical Division of Pediatric Nephrology and Gastroenterology<br>all Medical University of Vienna, Austria
**Monitor**<br><br>**(AMG §§ 2a, 35,36)**	Will be organized via the "Coordinating Centre for Clinical Trials, Medical University of Vienna"
**Clinical Trial Centres**	Single center: Department of Internal Medicine III, Clinical Division of Nephrology and Dialysis, Medical University Vienna, Austria
**Statistician**	Dr. Andreas Gleiss, Center for Medical Statistic, Informatics and Intelligent Systems, Section for Clinical Biometrics

Ao. Univ. Prof. Dr. Andreas Vychytil<br>(Investigator)	________________<br>Signature	_________<br>Date
Dr. Andreas Gleiss<br>(Statistician)	________________<br>Signature	__________<br>Date
Ao. Univ. Prof. Dr. Christoph Aufricht<br>(Representative of sponsor)	________________<br>Signature	__________<br>Date

LIST OF ABBREVATIONS

AE	adverse event
CRF	case report form
EOS	end of study examination
GCP	good clinical practice
HSP	heat shock protein
HSR	heat shock response
No.	number
PD	Peritoneal dialysis
PDF	Peritoneal dialysis fluid
SAE	serious adverse event
SUSAR	suspected unexpected adverse reaction
PET	Peritoneal Equilibration Test

Laboratory abbreviations:
Na^+, sodium; K^+, potassium; Ca^{2+}, Calcium; Cl^-, Chloride, AST, Aspartat-Aminotransferase or GOT; ALT, Alanin-Aminotransferase or GPT; CRP, C-reactive protein; GGT, gamma-Glutamyltransferase; LDH, lactate dehydrogenase; AP, alkaline phosphatase; CK creatine kinase; CRP, C-reactive protein); Hb, hemoglobin; HKT, hematocrit; RBC, red blood cell count; MCH, mean corpuscular hemoglobin; WBC, white blood cell count, TNFα tumor necrosis factor α,
IL-6 Interleucine 6

1 TABLE OF CONTENTS

SPONSOR, INVESTIGATOR, MONITOR AND SIGNATURES....3
1 TABLE OF CONTENTS....5
2 PROTOCOL SYNOPSIS....8
3 STUDY SCHEDULE (Table 1)....11
4 INTRODUCTion....14
4.1 Background information....14
4.2 Study rationale....16
5 INVESTIGATIONAL PLAN....17
5.1 Objective....17
5.2 Study population....17
5.2.1 Inclusion criteria....17
5.2.2 Exclusion criteria....17
5.2.3 Females of childbearing age....18
5.3 Overall study design and plan....18
5.4 Study treatment....19
5.4.1 Dose selection and treatment duration....19
5.5 Randomization and numbering....20
5.6 Benefit and risk assessment....21
6 STUDY PROCEDURES....22
6.1 Screening....22
6.2 Screening investigation....22
6.3 Run-in period....22
6.4 Treatment period 1 and treatment period 2....23
6.5 End-of-study....24
6.6 Study drug discontinuation....24
6.6.1 Study drug premature permanent discontinuation....24
6.7 Concomitant medication....25
6.8 Treatment after end of study....25
6.9 General rules for trial procedures....25
6.10 Withdrawal and replacement of subjects....25
6.10.1 Criteria for withdrawal....25

6.10.2 Follow-up of patients withdrawn from the study 26
6.10.3 Replacement policy 26
6.11 Premature termination of the study 26
7 METHODS OF EVALUATION 27
7.1 Physical examination 27
7.2 Weight and height 27
7.3 Vital signs 27
7.4 Electrocardiography 27
7.5 Peritoneal equilibration test (PET) 27
7.6 Hematology and clinical chemistry 28
7.7 Total expression of heat shock proteins 28
8 SAFETY DEFINITIONS AND REPORTING REQUIREMENTS 29
8.1 Averse events (AEs) 29
8.1.1 Definition of adverse events 29
8.2 Serious adverse events (SAEs) 29
8.2.1 Hospitalization – Prolongation of existing hospitalization 30
8.2.2 SAEs related to study-mandated procedures 30
8.2.3 Suspected unexpected serious adverse reactions (SUSARs) 31
8.3 Severity of adverse events 31
8.4 Relationship to study drug 31
8.5 Reporting procedures 32
8.5.1 Reporting procedures for SAEs 32
8.5.2 Reporting procedures for SUSARs 33
9 STATISTICAL METHODOLOGY AND ANALYSIS 33
9.1 Sample size considerations 33
9.2 Statistical Analyses 34
10 ETHICAL AND LEGAL ASPECTS 35
10.1 Informed consent of subjects 35
10.2 Acknowledgement / approval of the study 35
10.2.1 Protocol amendments 35
10.3 Finance and insurance 36
10.4 Confidentiality 36
10.5 Ethics and Good Clinical Practice (GCP) 36

11 DOCUMENTATION AND DATA MANAGEMENT 36
11.1 Documentation of study results 36
11.1.1 Case report form (CRF) 37
11.2 Safekeeping 37
11.3 Monitoring 37
11.4 Relevant Protocol Deviations 38
11.5 Publication of study results 38
11.6 Quality Control and Quality Assurance 38
11 REFERENCEs 39
12 APPENDICES 40
12.1 Appendix IMP 40
12.1.1 Dipeptiven 40
12.1.2 Dianeal PD4 Glucose 45
12.1.3 IMP Handling Instruction Ala-Gln in PD 47
12.1.4 Application Information Dipeptiven 49
12.1.5 Verification of Stability 52

2 PROTOCOL SYNOPSIS

**TITLE**	An Open Label, Randomized, Two-Period Crossover Study to Evaluate the Safety and Efficacy of the Addition of Alanyl-Glutamine-Dipeptide to Dialysis Solution in Peritoneal Dialysis (PD)
**ACRONYM**	Ala-Gln in PD
**OBJECTIVES**	**Primary Objective**<br>To evaluate treatment safety and efficacy of Alanyl-Glutamine-Dipeptide as addition to dialysis solution in PD.
**DESIGN / PHASE**	Prospective, single-center, open label, randomized, two period cross over, phase I-II study.
**CENTER(S) / COUNTRY(IES)**	Single center: Department of Internal Medicine III, Clinical Division of Nephrology and Dialysis, Medical University Vienna, Austria
**PATIENTS / GROUPS**	24 patients should complete the study, a maximum of 28 patients will be enrolled, subjects who discontinued from the study before the start of study drug therapy will be replaced.<br>Patients will be randomized into 2 groups in a cross-over design<br>Randomization ratio 1:1
**INCLUSION CRITERIA**	• Signed informed consent prior to any study-mandated procedure.<br>• Male and female patients aged ≥ 19<br>• Chronic renal failure; 2 months stable on PD<br>• no peritonitis within the previous 2 months<br>• Without severe concomitant disease.<br>• Negative pregnancy test in female patients of childbearing potential and adequate contraception in female patients of childbearing age.
**EXCLUSION CRITERIA**	• Known hypersensitivity to study medication.<br>• Treatment with another investigational drug within 1 month prior to start of study medication.<br>• Malignancy requiring chemotherapy or radiation<br>• Pregnancy or nursing,<br>• Presumed non-compliance.<br>• Limited efficacy of peritoneal dialysis due to anatomical anomalies or severe intraabdominal adhesions.<br>• Clinical significant inflammatory parameters<br>• Less than 50 kg body weight<br>• Immunosuppressive therapy
**STUDY DESIGN**	The study is a randomized, open-label, two-period crossover study in patients with chronic renal failure who are dependent on renal replacement therapy. 28 subjects who meet the inclusion/exclusion criteria will be randomized into one of two treatment sequences.<br>• Screening<br>• Run in-period (up to 4 weeks)<br>• Treatment period I consists of one single peritoneal dialysis exchange with standard PD solution <u>with</u> Alanyl-Glutamine–Dipeptide followed, after a wash out period (28 days + max

	7 days), by treatment period 2 consisting a single peritoneal dialysis exchange with standard PD solution without Alanyl-Glutamine-Dipeptide **OR** Treatment period I consists of a single peritoneal dialysis exchange with standard PD solution without Alanyl-Glutamine–Dipeptide followed, after a wash out period (28 days +max 7 days), by treatment period 2 consisting a single peritoneal dialysis exchange with standard PD solution with Alanyl-Glutamine-Dipeptide. Standard PDF in both arms will be Dianeal® PD4 Glukose 3,86% (produced by Baxter GmbH, Germany)<br>• EOS
**INVESTIGATIONAL DRUG (IMP)**	**IMP: Alanyl-Glutamine-Dipeptide (Dipeptiven)**<br><br>Dose: 17,4 ml Dipeptiven (=3,48g N(2)-L Alanyl-L-Glutamin) will be solute/dissolved at a final concentration of (0,174 % = 8 mmol / l) in 2 liters of standard PDF (Dianeal®PD4at PH:5,5; Glucose-Concentration 3,86 %)
**COMPARATIVE DRUG**	None
**EFFICACY ENDPOINTS**	**Primary**<br>• Total expression of heat shock proteins.<br>**Secondary**<br>• Clinical PET-test to measure specific transport kinetics in peritoneal cells (creatinine, urea, sodium, potassium, phosphor, glucose, protein,<br>• Cell number in peritoneal effluent<br>• cytokines (IL-6, TNFα)<br>• Cell function (phagocytosis and cytokine production)<br>• Morphology of peritoneal cells from effluent (cell culture)<br>• Biomarker CA125, IL-8
**TOLERABILITY / SAFETY ENDPOINTS**	Presence/absence/severity of side effects - No. and severity of AEs (including SAEs and SUSARs)
**STATISTICAL METHODOLOGY**	**Primary Endpoint:**<br>Total expression of heat shock proteins in peritoneal cells from dialysate samples<br>**Null and alternative hypotheses:**<br>**H_0**: The use of alanyl-glutamine-dipeptide in PDF does not change the total expression of heat shock proteins<br>**H_1**: The use of alanyl-glutamine-dipeptide in PDF changes the total expression of heat shock proteins<br>**Type-I and -II errors:**<br>Type-I error: 5%<br>Power: 80%<br>Type-II error: 1-power

	**Statistical methodology (primary outcome):**<br>Outcome variables will be described by their mean ± standard deviation at the respective time points in the treatment groups. Variables with skewed distributions will be transformed appropriately. To compare the percent change in the main outcome variable between the treatment groups, analysis of variance (ANOVA) models for cross-over designs will be used. Additionally, ANOVA models will be used to describe the differences in the secondary outcome variables between the treatment groups. Two-sided p-values smaller than 0.05 will be considered as indicating statistical significance.<br><br>**Sample size calculation:**<br>Sample size calculation is based on the percent change in the main outcome variable "total heat shock protein expression" in mesothelial cells after treatment with alanyl-glutamine-dipeptide as add-on to PDF compared to a control group. A sample size of 28 patients in a cross-over study design will have 80% power to detect a difference in means of 30 percentage points, using a 0.05 two-sided significance level (nQuery Advisor version 6). This calculation includes an expected 10% rate of drop-outs and is based on an assumed standard deviation of within-subject period differences of 50 percentage points. Since this standard deviation, however, is unknown a half-sampling will be performed: after the primary outcome results of a total of 20 patients are available, the standard deviation will be estimated and based on this the final sample size will be re-calculated.<br><br>**Other endpoints:**<br>Descriptive statistics

3 STUDY SCHEDULE (TABLE 1)

	**Schedule of Events**						
	**Screening**	**Run in Phase**	**Treatment 1**	**wash out**	**Treatment 2**	**EOS**	**Early Termination EOS**
**Assessment**	Day −35 to −21	Day −20 to -1	Day 0	28 days ± 7 days	Day after end of wash-out (+ max 7 days)	Immediately following treatment 2 (5th hour)	As soon as possible after patients withdraw
Informed consent	X						
Demography	X						
Inclusion\exclusion criteria	X		X		X		
Medical and medication history	X		X		X		
Body weight	X		X		X		X
Body height	X		X		X		X
Physical exam	X		X		X		X
Vital signs	X[a]		X[a]		X[a]	X	X
Serology for HIV, hepatitis B and hepatitis C	X						
Pregnancy screening (measurement of serum β-HCG)	X[b]			X[b;f]			
Clinical laboratory	X[c]		X[c]		X[c]	X	X
PET			X[g]		X[g]	X	
Analysis of Dialysates			X[d]		X[d]		
AE monitoring	X[e]	X[e]	X[e]	X	X		X
Concomitant medications	X	X	X	X	X		X
Randomisation			X				
PD +/- study medication			X		X		

HIV = human immunodeficiency virus;

a Vital signs include resting blood pressure, temperature, and pulse rate

b Female subjects of childbearing potential

c Clinical laboratory tests include haematology, clinical chemistry, blood gas analysis, serum amino acid levels,(Alanine, Glutamine, Alanyl-Glutamine-Dipeptide)

d Ultrafiltration, Creatinine, Glucose, Albumine, peritoneal cell count including cytokines (IL-6,IL-8 TNFα), CA 125, Alanine, Glutamine, Alanyl-Glutamine-Dipeptide, total peritoneal cellular HSP expression, BUN, Total protein, Sodium, Cellfunction, Morphology

e Any adverse events occurring after signing of informed consent, but before first treatment will be considered as part of the medical history

f at the end of wash out phase

g taking of a blood sample for PET

	Treatment Period 1					
	Day of Treatment					
**Hour**	-1h	0	1h	2h	4 h	5 h
Vital signs[a]	**X**	**X**		**X**	**X**	**X**
Physical Examination	**X**					
Body height, body weight	**X**					
PD +/- Study medication		←→	←→	←→	←→	
Clinical laboratory[c,d]	**X**					**X**
Taking of dialysate samples[b]		**X**	**X**	**X**	**X***	
Adverse event monitoring	←→	←→	←→	←→	←→	←→
Concomitant medication	←→	←→	←→	←→	←→	←→

[a] Vital signs include supine blood pressure, ear temperature and pulse rate

[b] Ultrafiltration*, Creatinine, Glucose, Albumine, Alanine, Glutamine, Alanyl-Glutamine-Dipeptide , total peritoneal cellular HSP expression*, peritoneal cell count including cytokines (IL-6,IL-8, TNFα) *,CA 125*, BUN, Sodium, total Protein, Cellfunction*, Morphology*

[c] Clinical laboratory tests include haematology, clinical chemistry, blood gas analysis, serum amino acid levels Alanine, Glutamine, Alanyl-Glutamine-Dipeptide

[d] Parameters of PET blood sample are identical to Parameters of Clinical Lab Test

	Treatment Period 2/EOS: Schedule of Events					
	Day of Treatment					
**Hour**	-1h	0	1h	2h	4 h	In 5th hour → EOS
Vital signs[a]	**X**	**X**		**X**	**X**	**X**
Physical Examination	**X**					
Body height, body weight	**X**					
PD +/- Study medication		←→	←→	←→	←→	
Clinical laboratory[c,d]	**X**					**X**
Taking of dialysate samples[b]		**X**	**X**	**X**	**X***	
Adverse event monitoring	←→	←→	←→	←→	←→	←→
Concomitant medication	←→	←→	←→	←→	←→	←→

[a] Vital signs include supine blood pressure, ear temperature and pulse rate

[b] Ultrafiltration*, Creatinine, Glucose, Albumine, Alanine, Glutamine, Alanyl-Glutamine-Dipeptide , total peritoneal cellular HSP expression*, peritoneal cell count including cytokines (IL-6,IL-8, TNFα) *,CA 125*, BUN, Sodium, total Protein, Cellfunction*, Morphology*

[c] Clinical laboratory tests include haematology, clinical chemistry, blood gas analysis, serum amino acid levels Alanine, Glutamine, Alanyl-Glutamine-Dipeptide

[d] Parameters of PET blood sample are identical to Parameters of Clinical Lab Test

4 INTRODUCTION

4.1 Background information

One out of ten patients on dialysis is treated with PD in Europe as alternative to hemodialysis (1, 2). In this form of treatment a glucose-based hyper-osmolaric PD fluid (PDF) is repeatedly filled into the peritoneum with a special implanted dialysis catheter, kept there for several hours and afterwards drained from the abdominal cavity. Depending on its physico-chemical properties, PDF removes solutes and water from the uremic patient and allows survival despite chronic renal failure.

Due to their position as barrier between the cytotoxic PDF and the peritoneal wall, mesothelial cells are exposed to repeated metabolic and biomechanical insults which can result in severe functional and/or structural injuries (3-5). Thus, PDF are cytotoxic and up to a third of PD-patients will suffer from technical failure (6).

The heat shock response (HSR) is a highly conserved adaptive cell response to diverse environmental and physiological stressors, classically such as increased temperature, chemical toxicants, energy depletion and mechanical stress (7). The HSR results in the immediate induction of effector proteins called heat shock proteins (HSP), categorized by molecular size and functional class. In previous studies, we have demonstrated that the cytotoxic properties of PDF not only result in disruption of the cell homoeostasis but also induce the HSR: the expression of HSP was found up-regulated in mesothelial cells following recovery from acute short term exposure to stressors such as acidosis, high lactate and high glucose concentration (8-12). As expected, experimental over-expression of HSP induced by pretreatment with heat or by transient Hsp72 transfection conferred increased cellular resistance and resulted in HSP-mediated cytoprotection in experimental PD (11, 12). Thus, HSR and its effectors enable cellular survival and induce an increased resistance against repeated injuries. Chronic peritoneal damage in PD represents an imbalance between cellular injury and repair processes.

Unexpectedly, however, HSP expression was found to be low in mesothelial cell in peritoneal biopsies from PD patients but also from rats on experimental PD (12, 13). Whereas we found HSP positive mesothelial cells in the peritoneal effluent, no HSP up-regulation was discernable in peritoneal adherent cells (12). At first view, these findings appear in contradiction to our previous results in the cell culture system and could not be explained sufficiently.

However, newer studies revealed that the acute exposure models selectively reflected the intraperitoneal conditions during the early dwell phase during PD, limiting insights into the first cellular responses induced by PDF cytotoxicity. As cytotoxic properties of PDF are known to be rapidly adapted and diluted in the in-vivo situation, a more continuous exposure system to diluted PDF was needed to reflect the intraperitoneal conditions during the later phases of the PD dwell. In this setting, that should be particularly relevant for cell recovery mechanisms, exposure to diluted PDF indeed resulted in down-regulation of HSR in cultured human mesothelial cells (14).

Such an inadequate cellular stress response has previously been postulated in other glucose toxicity related diseases such as diabetes. In diabetic patients, peripheral blood lymphocytes had a significantly lower up regulation of HSP expression after ex-vivo treatment with heat-shock than those of healthy controls (15, 16). The data of our study are the first that clearly demonstrate suppressed HSR in a reproducible well controlled experimental system.

The adequacy of the HSR is known to be affected by glutamine, a conditionally-essential amino acid, in several clinical and experimental settings (17, 18). Under conditions with low plasma glutamine, such as during critical illness and sepsis, recent analysis revealed that glutamine-starving cells are unable to express adequate amounts of the Hsp72 family. Following experimental glutamine depletion, the half-life of Hsp72 mRNA was reduced, resulting in depressed Hsp72 expression in primary monocytes following *ex-vivo* heat treatment (19). Molecular weights and charges of glutamine and creatinine are similar, and comparable rates of transperitoneal transport have been reported (20, 27). Thus, peritoneal levels of glutamine can be expected to be markedly lower than corresponding plasma levels, likely resulting in glutamine starvation of peritoneal cells. As shown in the figure below, glutamine depletion also aggravated HSP depression and increased mesothelial cell vulnerability upon PDF exposure.

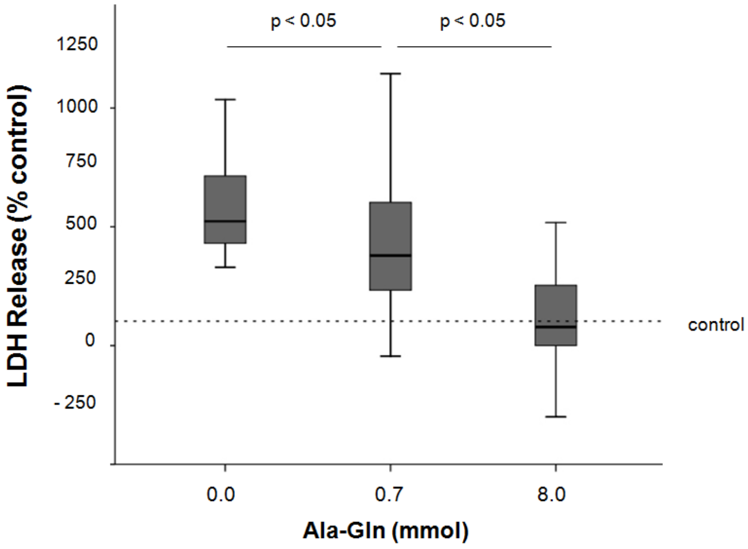

Figure 1: Influence of glutamine levels on cell survival and stress response in human cultured mesothelial cells in the *in-vitro* PDF exposure model. Depletion of glutamine results in exacerbated cellular damage. Pharmacologic addition of supraphysiologic levels of alanyl-glutamine results in normalized cellular survival, indicating an adequate heat shock response (HSR).

In a recent study, addition of glutamine at 8 mmol/l (= 0.174%) to PDF enhanced mesothelial HSP expression and improved cellular survival upon *in-vitro* PDF exposure, and protected peritoneal membrane integrity in the acute *in-vivo* model of PD (21). However, glutamine is a relatively unstable amino acid, only poorly soluble in aqueous solution, and is therefore preferably used in the clinical setting as a dipeptide (22). Many experimental and clinical studies (17, 18, 23-26) have proven that supraphysiological intravenous alanyl-glutamine supplementations are save, enhance HSP expression, and improve outcome at cellular and systemic level. Effects of addition of alanyl-glutamine dipeptide on HSP expression were consistent, ranging from about 30% in ex-vivo studies in primary cultures of mesothelial cells grown from peritoneal effluates of PD patients, to more than threefold in cells harvested from PD effluates in the acute rat *in-vivo* model of PD (unpublished data).

PD represents a unique opportunity for the implementation of interventions with HSR. First, PD is, as a therapeutic concept, strictly based on predictable, perfectly timed and dosed “repetitive insults” to the peritoneal membrane. Moreover, PD represents an interesting setting, as recent data demonstrate clear evidence for a suppressed HSR during injurious exposure to “cytotoxic” PDF – whereas addition of alanyl-glutamine to PDF restored HSP expression in the *in-vitro* PD model, associated with stabilization of the membrane integrity of human peritoneal mesothelial cells derived from the dialyzed peritoneal cavity. Finally, data from the 4 week chronic *in-vivo* PD rat model also suggest therapeutic potential of the addition of alanyl-glutamine to PDF, as this intervention extended cytoprotective effects of glutamine to reduced peritoneal scarring, as shown in the figure below.

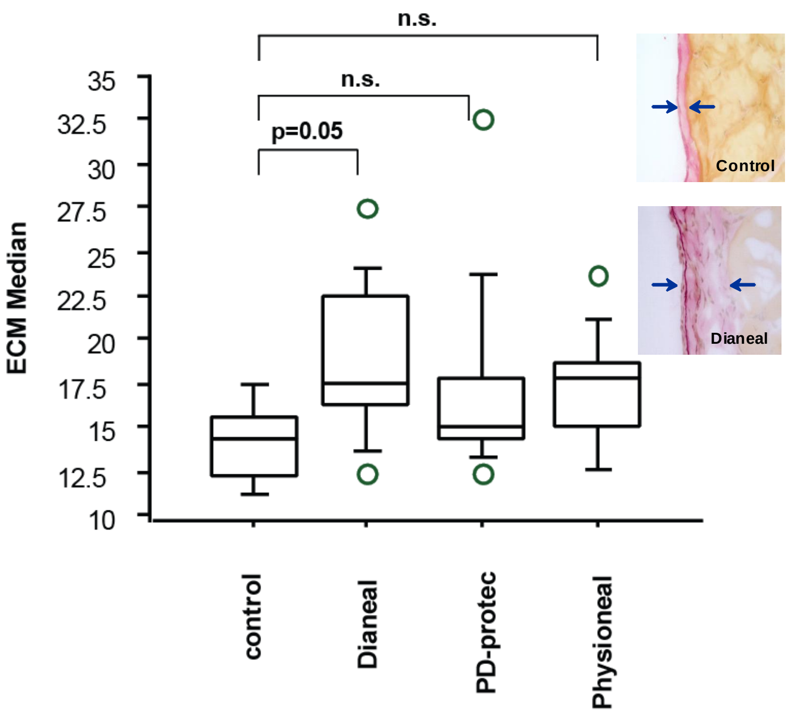

Figure 2: Effects of addition of alanyl-glutamine to PDF in the chronic Amsterdam rat model of PD. Extracellular matrix (ECM) is measured in peritoneal biopsies from rats, treated with standard PDF, PDF + alanyl-glutamine (8 mmol) or a more physiological PDF for 4 weeks. Whereas standard PDF significantly induces peritoneal scarring, the addition of alanyl-glutamine reduced peritoneal damage to control.

Taken together, the preclinical results introduce the inadequate cellular stress response of mesothelial cells following PDF exposure as new and potential relevant pathomechanism in PD. Supplementation of PDF with pharmacological doses of alanyl-glutamine restored HSP expression and increased the resistance of mesothelial cells in *in-vitro* models of PD and preserved peritoneal integrity in *in-vivo* models of PD.

4.2 Study rationale

Peritoneal dialysis (PD) is a cost effective and safe form of renal replacement therapy in patients suffering from end stage renal disease.

However currently available PDF are not biocompatible for the peritoneal cavity and its cells. Acute cytotoxic effects of the majority of the current glucose-based PDF are caused by low pH, lactate, high glucose and its degradation products (GDP).

Toxic effects of PDF can thus be extended to suppression of mesothelial HSR following PDF exposure resulting in increased susceptibility of mesothelial cells against PDF exposure: PDF inherent stress factors fail to adequately induce HSP as effectors of the cellular stress response – the adequate HRS rather seems to be blocked.

Hence, therapeutic approaches to activate and enhance the HSR will reduce peritoneal damage and organ failure and improve the survival of organisms.

Preclinical results demonstrated that supplementation of PDF with pharmacological doses of alanyl-glutamine restored HSP expression and increased the resistance of mesothelial cells in *in-vitro* models of PD and preserved peritoneal integrity in *in-vivo* models of PD.

After these positive preclinical results, this study shall now clarify, whether the addition of alanyl-glutamine to the most commonly used glucose-based PDF is safe and tolerable. Therefore PDFs will be drained in a randomized cross-over study. Main outcomes measures will be total HSP expression in peritoneal cells and changes of the peritoneal transport kinetics and the presence/absence/severity of side effects.

5 INVESTIGATIONAL PLAN

5.1 Objective

The objective of this study is to evaluate the safety and efficacy of the addition of alanyl-glutamine-dipeptide to dialysis solutions in "Peritoneal Dialysis" (PD) patients.

5.2 Study population

28 patients with chronic renal failure will be included in this study

5.2.1 Inclusion criteria

- Signed informed consent prior to any study-mandated procedure.
- Male and female patients aged ≥ 19
- Chronic renal failure; stable for 2 months
- no peritonitis within the previous 2 months
- Without severe concomitant disease.
- Negative pregnancy test and adequate contraception in female patients of childbearing potential

5.2.2 Exclusion criteria

- Known hypersensitivity to study medication.
- Treatment with another investigational drug within 1 month prior to start of study medication.
- Malignancy requiring chemotherapy or radiation
- Pregnancy or nursing.
- Presumed non-compliance.
- Limited efficacy of peritoneal dialysis due to anatomical anomalies or severe intraabdominal ad-hesions.
- Clinical significant inflammatory parameters
- Less than 50 kg body weight
- Immunosuppressive therapy

5.2.3 Females of childbearing age

Females can be included if they are unlikely to conceive, as indicated by at least one of the following points:

- Surgical sterilization
- Postmenopausal female > 45 years of age with > 2 years since last menses
- Non-sterilized, premenopausal, non-amenorrhoe female who agrees to abstain from heterosexual activity or to use an adequate method of contraception. Acceptable methods of birth control are intrauterine device (IUD), diaphragma with spermicide, vasectomy, condom or hormonal contraceptives.

A pregnancy test will be performed in the screening phase and at the end of wash out phase.

5.3 Overall study design and plan

The study is a randomized, open-label, two-period crossover study in patients with chronic renal failure who are dependent on renal replacement therapy. 28 subjects who meet the inclusion/exclusion criteria will be randomized 1:1 into one of two treatment sequences in a single exchange.
The overall duration per patient will be 30 – 44 days. This variability arises due to the fact, that the wash out period has a maximum spread of ± 7 days.

- ***Screening***
- ***Run-in period (4 weeks)***

Treatment period I consists of one single peritoneal dialysis exchange with standard PD solution with alanyl-glutamine-dipeptide followed, after a ***wash out period*** (28 days + max 7 days), by ***Treatment period 2*** consisting of a single peritoneal dialysis exchange with standard PD solution without alanyl-glutamine-dipeptide **OR** treatment period I consisting of a single peritoneal dialysis exchange with standard PD solution without alanyl-glutamine-dipeptide followed, after a wash out period (28 days + max. 7 days), by treatment period 2 consisting of a single peritoneal dialysis exchange with standard PD solution with alanyl-glutamine-dipeptide

PD patients of the Department of Internal Medicine III, Clinical Division of Nephrology and Dialysis, Medical University of Vienna are assessed for eligibility and may be contacted. It is not planned to include further study centers.

In the treatment period I patients receive either PDF with or without alanyl-glutamine (8 mmol / l = 0,174 %) for PD. In the second study phase PDFs will be crossed over. This study is designed to assess the efficacy and safety of alanyl-glutamine in patients with PD. Efficacy is evaluated primarily by changes in the peritoneal transport kinetics.

For the study schedule see Table 1. Patients will undergo a screening investigation prior to study start. After 4 weeks of run-in there will be the first phase of treatment, 28 days (+ max. 7 days) of wash-out will follow, prior to second study phase.

Immediately following Treatment period 2 (day after end of wash-out), an End of Study visit (EOS) including vital signs, clinical laboratory, AE monitoring, and recording concomitant medications is planned.

5.4 Study treatment

Study drug is alanyl-glutamine-dipeptide. Subjects will be randomized (1:1) to receive either the study drug in the first treatment period or in the second treatment period as add-on to PDF (Dianeal® PD4 Glucose3,86 %).

Substance: Alanyl-glutamine-dipeptide will be made available by Zytoprotec GmbH, which is a Spin-off of the Medical University of Vienna, and holds a patent 'Carbohydrate-based peritoneal dialysis fluid comprising glutamine residue' (International Publication Number: WO 2008/106702 A1)..

IMP will be stored at the pharmacy of the Medical University of Vienna and prepared for administration by a qualified and authorized person at the site.

Administration: 17,4 ml Dipeptiven will be added to 2 liters of PD solution (Dianeal® PD4 Glucose 3,86 %, PH: 5.2-5.5).

The mixture of Dipeptiven and PD solution (final concentration 8 mmol / l = 0,174 % alanyl-glutamine dipeptide, resulting in a total osmolality of the modified PDF of 491 mosm/l and a pH of 5.2- 5.5) should be used immediately after preparation.

For further information due to preparation of the IMP and verification of compartibilty and stability, please see Appendices 12.1.2. "IMP Handling Instruction", 12.1.3 Summary of Product Characteristics "Dianeal PD 4 Glucose", 12.1.4. "Application Information Dipeptiven" and 12.1.5. "Verification of Stability".

5.4.1 Dose selection and treatment duration

Alanyl-glutamine-dipeptide (Dipeptiven) is a concentrate to produce a solution of infusion and currently approved for i.v. use in patients with e.g. hypercatabolic and/or hypermetabolic conditions.

Dipeptiven should not be stored above 25°C and is filled in glass bottles (colourless, rubber plug).

Interactions and reverse reactions could not be observed under normal applications.

For more information, please read the "Summary of Characteristics" in the appendix.(12.1.1)

Prior in-vitro studies with human immortalized mesothelial cells and alanyl-glutamine dipeptide concentrations of 4, 8, 10, 15 and 20 mmol/l showed no cytotoxicity at any time. The cytoprotective effect and increased HSP expression upon PDF exposure were markedly induced starting at the concentration of 8 mmol / l = 0,174 %.

So the concentration of 8 mmol / l = 0,174 % will be used in this study. Based on this concentration, a 2 l bag of modified PDF contains 3,48 g alanyl-glutamine dipeptide, resulting in a maximum intraperitoneal dosage of 0,07 g per kg BW in a patient with the minimal body weight of 50kg. There are no human data of the peritoneal resorption kinetics of the dipeptide available, the peritoneal kinetics of glutamine have been shown to be comparable to that of creatinine (reference 27). Thus, the planed exposure to the study drug is markedly lower than the maximal daily dosage (0,3-0,4 g/kg BW) and the maximal "hourly infusion rate" (0,1 g/kg/hr), both given in the literature for direct intravenous administration.

5.5 Randomization and numbering

Subjects will be randomized immediately before starting of treatment period 1. After positive screening visit a random allocation with a randomization ratio of 1:1 will be performed.

Randomization will be performed through central allocation of a randomization schedule prepared through a computer-generated random listing of the two treatment assignments blocked in groups. Randomization will be done by randomizer.org.

At randomization, the patient will be randomly assigned to either treatment group 1 or 2. In first study period, group 1 will receive alanyl-glutamine-dipeptide as add-on to standard PDF; group 2 will only receive standard PDF. In the second study phase groups will be crossed-over.

Thus, numbering of subjects will be performed as follows:

Three categories of identification codes are applicable:

- At screening subjects are numbered beginning with 01 and identified as S_ _ (screening number).
- At randomization subjects receive unique identification numbers beginning with 01. They are numbered on an ongoing base ID _ _.
- The computer randomization will provide a list with the numbers of the patients in group 1 and 2. In the second study period treatments will be crossed-over.

Subject identification list: The assignment of a screening number for the subject identification is based on the obligation for anonymity. Only their screening and/or identification number, initials (first name and last name) and date of birth will identify the subjects' CRFs in the data analysis.

24 subjects should complete the study; a maximum of 28 patients will be enrolled, subjects who discontinued from the study before start of the study drug therapy, will be replaced.

5.6 Benefit and risk assessment

Worldwide, 200,000 patients suffer from kidney failure or end-stage renal disease (ESRD) and depend on peritoneal dialysis. Peritoneal dialysis is a process of removing metabolic waste products and excess water from the patient's body, replacing the function of the diseased kidneys.

Treatment with PD provides benefits both in terms of quality of life of the patient and in terms of lower costs incurred to the health care system, but current standard PD fluids damage the patients' peritoneum over time, limiting the patient's survival time on this treatment.

Currently, despite the clear benefits of PD, long-term treatment is limited due to an inherent lack of biocompatibility of current PD fluids. The combination of standard PDFs and alanyl-glutamine-dipeptide is designed as a cytoprotective PDF that allows a significantly longer duration of the treatment.

6 STUDY PROCEDURES

6.1 Screening

Screening procedures for the study will be performed from day -35 till day -29 before starting treatment period 1. Screening assessments can be performed only after the patient has signed the informed consent form. Inclusion and exclusion criteria as listed above will be checked.

6.2 Screening investigation

Patients treated with PD which are potentially eligible for this study and have signed the informed consent will undergo the screening investigation (see Table 1). If they comply with all inclusion and exclusion criteria they may be randomized for study therapy. If there is temporary presence of an exclusion criterion (e.g. a current episode of fever), the inclusion and exclusion criteria may be re-evaluated at a later time point.

The following examinations and tests will be carried out in each subject at the screening examination

- Anamnesis of relevant medical history and current medication
- Informed consent
- Demography
- Inclusion/exclusion criteria
- Medical and medication history
- Body weight
- Body height
- Physical exam
- Vital signs
- Serology for HIV, hepatitis B and hepatitis C

- Clinical laboratory
- AE monitoring
- Concomitant medication
- Pregnancy screening (measurement of serum Beta-HCG) in female patients of childbearing potential
- Efficacy of Dialysis (Creatinine Clearance, kT/V)

6.3 Run-in period

Run-in phase is from day -28 till -1. During this period AE will be monitored and concomitant medication will be checked.

6.4 Treatment period 1 and treatment period 2

At baseline (before treatment) following parameters will be checked again:

- Inclusion/exclusion criteria
- Medical and medication history
- Body weight
- Body height
- Physical exam
- Vital signs
- Clinical Laboratory
- AE monitoring
- Concomitant medications

One group will receive standard PDF; the other group will receive standard PDF with alanyl-glutamine-dipeptide as add-on. For treatment period 2, groups will be crossed-over. The same parameters as in treatment period 1 will be evaluated.

The following parameters will be checked during the treatment during the PDF exchange:

- Vital signs
- Clinical laboratory including analysis of dialysate
- AE-monitoring
- Concomitant medication
- Efficacy of dialysis
- Amino acid levels (Alanine, Glutamine, Alanyle-Glutamine-Dipeptide) in serum & dialysate
- PET-Test dialysate (min 0, 60, 120 and 240; measures specific transport kinetics in peritoneal cells : creatinine, urea-nitrogene (BUN), sodium, potassium, phosphor, glucose, protein, albumine)
- Cell number and cytokines (IL-6, TNFα) in peritoneal effluent
- Cell function (phagocytosis and cytokine production)
- Morphology of peritoneal cells from effluent (cell culture)
- Ultrafiltration
- Biomarker CA125, IL-8

- Total expression of heat shock proteins in peritoneal cells from dialysate

6.5 End-of-study

The end of study visit will be performed following treatment period 2, in 5th hour.

Following parameters will be checked:

- Vital signs
- AE-monitoring
- Concomitant medication
- Clinical laboratory

6.6 Study drug discontinuation

"The investigator must temporarily interrupt or permanently discontinue the study drug if continued administration of the study drug is believed to be contrary to the best interests of the patient."

Due to the fact, that the IMP will be administered just for a single dose, the study drug may not be interrupted but discontinued.

The premature discontinuation of study drug might be triggered by an AE, a diagnostic or therapeutic procedure, an abnormal assessment (e.g., laboratory abnormalities), or for administrative reasons, in particular withdrawal of the patient's consent.

The reason for study drug premature permanent discontinuation must be documented in the CRF.

In case of need of a premature discontinuation, PDF with IMP will be exchanged by standard PDF, relevant clinical measures will be done and the monitoring of the patient will be continued (also see 6.6.1).

6.6.1 Study drug premature permanent discontinuation

Study drug premature permanent discontinuation due to an adverse event

If the reason for premature permanent discontinuation of study treatment is an AE, the patient should have a premature EOS visit with all the assessments foreseen for the EOS, whenever possible, and as soon as possible. If the reason for EOS is disease worsening, the "event" of disease progression should be adequately documented, and be communicated and discussed with the sponsor prior to EOS.

Study drug premature permanent discontinuation due to another reason than adverse event

If the reason for premature permanent discontinuation of study treatment is not an AE, the patient should be withdrawn from the study (withdrawal of consent) and have the EOS visit with all the assessments performed before or at the day of the study drug discontinuation, whenever possible.

6.7 Concomitant medication

The well-being of the patient has the first priority, and modifications of concomitant treatment during the trial are allowed as necessary. They should be documented in the patient's log.

In this study, all concomitant medications are allowed.

6.8 Treatment after end of study

After the end of study standard PD will be performed.

6.9 General rules for trial procedures

- All study measures like blood sampling and measurements (vital parameters, etc.) have to be documented with date (dd:mm:yyyy). In addition, the time (hh:mm) should be taken for PET.
- In case several study procedures are scheduled at the same time point, there is no specific sequence that should be followed.
- The dates of all procedures should be according to the protocol. The time margins mentioned in the study flow chart (Table 1) are admissible. If for any reason, a study procedure is not performed within scheduled margins a protocol deviation should be noted, and the procedure should be performed as soon as possible or as adequate.
- If it is necessary for organizational reasons, it is admissible to perform procedures which are scheduled for one visit at two different time points (e.g. blood drawing and PET on different days). Allowed time margins should thereby not be exceeded.

6.10 Withdrawal and replacement of subjects

6.10.1 Criteria for withdrawal

Subjects may prematurely discontinue from the study at any time. Premature discontinuation from the study is to be understood when the subject did not undergo EOS examination and / or all pivotal assessments during the study.

Subjects must be withdrawn under the following circumstances:

- at their own request
- if the investigator feels it would not be in the best interest of the subject to continue
- if the subject violates conditions laid out in the consent form / information sheet or disregards instructions by the study personal

In all cases, the reason why subjects are withdrawn must be recorded in detail in the CRF and in the subject's medical records. Should the study be discontinued prematurely, all study materials (complete, partially completed and empty CRFs) will be retained. All protocol deviations should be documented in the source documents.

6.10.2 Follow-up of patients withdrawn from the study

In case of premature discontinuation after study drug intake, the investigations scheduled for the EOS visit should be performed as soon as possible. The subjects will be advised that participation in these investigations is voluntary, but recommended for their own safety. Furthermore, they may request that from the time point of withdrawal no more data will be recorded and that all biological samples collected in the course of the study will be destroyed.

6.10.3 Replacement policy

Subjects who discontinued from the study for any reason before start of study drug therapy will be replaced. Subjects who discontinue after first exposure to study drug will be included in the intention-to-treat analysis and will not be replaced.

6.11 Premature termination of the study

The sponsor has the right to close this study at any time. The IEC and the competent regulatory authority must be informed.

The trial will be terminated prematurely in the following cases:

- If adverse events occur which are so serious that the risk-benefit ratio is not acceptable.
- If the number of drop-outs is so high that proper completion of the trial cannot realistically be expected.

7 METHODS OF EVALUATION

7.1 Physical examination

Physical examination is performed during the course of the study and results will be recorded in the CRF. Clinically relevant findings which were present prior to study drug initiation must be recorded on the relevant Medical History CRF page. Clinically relevant findings found after study drug initiation and meeting the definition of an AE (new AE or worsening of previously existing condition) must be recorded on an AE page in the CRF.

7.2 Weight and height

Body weight and height are measured at screening and EOS (body weight only) and the results will be recorded in the CRF. Body weight is assessed as dry weight (=PDF is substracted). The body-mass index will be calculated.

7.3 Vital signs

Blood pressure (systolic and diastolic) and pulse rate are measured using an automatic device. Measurements shall be recorded from the subject after having rested for at least 5-10 minutes.

Significant findings made after randomization, which meet the definition of an AE, must be recorded on an AE page in the CRF.

7.4 Electrocardiography

Abnormal findings have to be described by the investigator.

Significant new findings made after randomization, which meet the definition of an AE, must be recorded on an AE page in the CRF.

7.5 Peritoneal equilibration test (PET)

The peritoneal equilibration test (PET) is a semiquantitative assessment of peritoneal membrane transport function in patients on peritoneal dialysis (PD). The solute transport rates are assessed by the rates of their equilibration between the peritoneal capillary blood and dialysate. The ratio of solute concentrations in dialysate and plasma (D/P ratio) at specific times (t) during the dwell signifies the extent of solute equilibration. This ratio can be determined for any solute that is transported from the capillary blood to the dialysate and vice versa. Creatinine, glucose, urea, electrolytes, phosphate, and proteins are the commonly tested solutes for clinical use, in this study alanine, glutamine and alanyl-glutamine-dipeptide also will be measured to allow assessment of the respective resorption kinetics.,

Significant findings made after randomization, which meet the definition of an AE, must be recorded on an AE page in the CRF.

- PDF is introduced (0 minutes)
- Dialysate withdrawal after 0, 60 and 120 minutes
- Last dialysate withdrawal and complete drainage after 240 minutes
- Blood withdrawal in -1 hour and hour 5th hour

7.6 Hematology and clinical chemistry

The following parameters will be determined in the central laboratory of the clinical trial center:

- Clinical chemistry (Na^+, K^+, Ca, AST, ALT, GGT, LDH, alkaline phosphatase, total bilirubin, creatine kinase, glucose, creatinine, blood urea nitrogen, albumin, total protein, CRP, total cholesterol, triglycerides)
- Hematology (hemoglobin, hematocrit, RBC, MCH, HCV, RDW, WBC, platelet count)
- Blood gas analysis and Acid-base metabolism will be recorded at screening and the treatment period
- Serum amino acid levels

Significant findings made after randomization, which meet the definition of an AE, must be recorded on an AE page in the CRF.

7.7 Total expression of heat shock proteins

Heat shock proteins (HSP) are a class of functionally related proteins whose expression is increased when cells are exposed to elevated in stress situations as elevated temperature or infection. Supplementation of PDF with pharmacological doses of alanyl-glutamine restored HSP expression and increased the resistance of mesothelial cells in *in-vitro* models of PD and preserved peritoneal integrity in *in-vivo* models of PD. In this study, an increase of total heat shock protein expression will be detected in cells from the peritoneal effluent at 240 min by staining of the cytospin and by Western blot analysis of the cellular pellet of the effluent (detailed methodology adopted from 12, 21).

8 SAFETY DEFINITIONS AND REPORTING REQUIREMENTS

8.1 Averse events (AEs)

8.1.1 Definition of adverse events

An AE is any adverse change from the subject's baseline condition, i.e., any unfavorable and unintended sign including an abnormal laboratory finding, symptom or disease which is considered to be clinically relevant by the physician that occurs during the course of the study, whether or not considered related to the study drug.

Adverse events include:

- Exacerbation of a pre-existing disease.
- Increase in frequency or intensity of a pre-existing episodic disease or medical condition.
- Disease or medical condition detected or diagnosed after study drug administration even though it may have been present prior to the start of the study.
- Continuous persistent disease or symptoms present at baseline that worsen following the start of the study.
- Lack of efficacy in the acute treatment of a life-threatening disease.
- Events considered by the investigator to be related to study-mandated procedures.
- Abnormal assessments, e.g., physical examination findings, must be reported as AEs if they represent a clinically significant finding that was not present at baseline or worsened during the course of the study.
- Laboratory test abnormalities must be reported as AEs if they represent a clinically significant finding, symptomatic or not, which was not present at baseline or worsened during the course of the study or led to dose reduction, interruption or permanent discontinuation of study drug.

Adverse events do not include:

- Medical or surgical procedure, e.g., surgery, endoscopy, tooth extraction, transfusion. However, the event leading to the procedure is an AE. If this event is serious, the procedure must be described in the SAE narrative.
- Pre-existing disease or medical condition that does not worsen.
- Situations in which an adverse change did not occur, e.g., hospitalizations for cosmetic elective surgery or for social and/or convenience reasons.
- Overdose of either study drug or concomitant medication without any signs or symptoms. However, overdose must be mentioned in the Study Drug Log.

8.2 Serious adverse events (SAEs)

A Serious Adverse Event (SAE) is defined by the International Conference on Harmonization (ICH) guidelines as any AE fulfilling at least one of the following criteria:

- Fatal (including fetal death).

- Life-threatening – defined as an event in which the subject was, in the judgment of the investigator, at risk of death at the time of the event; it does not refer to an event that hypothetically might have caused death had it been more severe.
- Requiring subject's hospitalization or prolongation of existing hospitalization – inpatient hospitalization refers to any inpatient admission, regardless of length of stay.
- Resulting in persistent or significant disability or incapacity (i.e., a substantial disruption of a person's ability to conduct normal life functions).
- Congenital anomaly or birth defect.
- Is medically significant or requires intervention to prevent at least one of the outcomes listed above.

Life-threatening refers to an event in which the subject was at risk of death at the time of the event. It does not refer to an event that hypothetically might have caused death if it were more severe.

Important medical events that may not immediately result in death, be life-threatening, or require hospitalization may be considered as SAEs when, based upon appropriate medical judgment, they may jeopardize the subject and may require medical or surgical intervention to prevent one of the outcomes listed in the definitions above.

8.2.1 Hospitalization – Prolongation of existing hospitalization

Hospitalization is defined as an overnight stay in a hospital unit and/or emergency room.

An additional overnight stay defines a prolongation of existing hospitalization.

The following is not considered an SAE and should be reported as an AE only:

- Treatment on an emergency or out subject basis for an event not fulfilling the definition of seriousness given above and not resulting in hospitalization.

The following reasons for hospitalizations are not considered AEs, and therefore not SAEs:

- Hospitalizations for cosmetic elective surgery, social and/or convenience reasons.
- Standard monitoring of a pre-existing disease or medical condition that did not worsen, e.g., hospitalization for coronary angiography in a subject with stable angina pectoris.
- Elective treatment of a pre-existing disease or medical condition that did not worsen, e.g., hospitalization for chemotherapy for cancer, elective hip replacement for arthritis.

8.2.2 SAEs related to study-mandated procedures

Such SAEs are defined as SAEs that appear to have a reasonable possibility of causal relationship (i.e., a relationship cannot be ruled out) to study-mandated procedures (excluding administration of study drug) such as discontinuation of subject's previous treatment during a washout period, or complication of a mandated invasive procedure (e.g., blood sampling, heart catheterization), or car accident on the way to the hospital for a study visit, etc.

8.2.3 Suspected unexpected serious adverse reactions (SUSARs)

SUSARs are all suspected adverse reactions related to the study drug that are both unexpected (not previously described in the SPC or Investigator's brochure) and serious.

8.3 Severity of adverse events

The severity of clinical AEs is graded on a three-point scale: mild, moderate, severe, and reported on specific AE pages of the CRF.

If the severity of an AE worsens during study drug administration, only the worst intensity should be reported on the AE page. If the AE lessens in intensity, no change in the severity is required.

If an AE occurs during a washout or placebo run-in phase and afterwards worsens during the treatment phase, a new AE page must be filled in with the intensity observed during study drug administration.

- **Mild**

  Event may be noticeable to subject; does not influence daily activities; the AE resolves spontaneously or may require minimal therapeutic intervention;

- **Moderate**

  Event may make subject uncomfortable; performance of daily activities may be influenced; intervention may be needed; the AE produces no sequelae.

- **Severe**

  Event may cause noticeable discomfort; usually interferes with daily activities; subject may not be able to continue in the study; the AE produces sequelae, which require prolonged therapeutic intervention.

A mild, moderate or severe AE may or may not be serious. These terms are used to describe the intensity of a specific event (as in mild, moderate, or severe myocardial infarction). However, a severe event may be of relatively minor medical significance (such as severe headache) and is not necessarily serious. For example, nausea lasting several hours may be rated as severe, but may not be clinically serious. Fever of 39°C that is not considered severe may become serious if it prolongs hospital discharge by a day. Seriousness rather than severity serves as a guide for defining regulatory reporting obligations.

8.4 Relationship to study drug

For all AEs, the investigator will assess the causal relationship between the study drug and the AE using his/her clinical expertise and judgment according to the following algorithm that best fits the circumstances of the AE:

- Unrelated
  - May or may not follow a reasonable temporal sequence from administration of the study product

  - Is biologically implausible and does not follow known response pattern to the suspect study drug (if response pattern is previously known)
  - Can be explained by the known characteristics of the subject's clinical state or other modes of therapy administered to the subject.

- Possible related
  - Follows a reasonable temporal sequence form administration of the study drug.
  - May follow a known response pattern to the study drug (if response pattern is previously known).
  - Could not be reasonable not be reasonably explained by the known characteristics of the subject's clinical state or other modes of therapy administered to the subject, if applicable.

- Definitely related
  - Follows a reasonable temporal sequence form administration of the study drug.
  - Follows a known response pattern to the study drug (if response pattern is previously known).
  - No other reasonable cause is present.

8.5 Reporting procedures

A special section is designated to adverse events in the case report form. The following details must thereby be entered:

- Type of adverse event
- Start (date and time)
- End (date and time)
- Severity (mild, moderate, severe)
- Serious (no / yes)
- Unexpected (no / yes)
- Outcome (resolved, ongoing, ongoing – improved, ongoing – worsening)
- Relation to study drug (unrelated, possibly related, definitely related)

Adverse events are to be documented in the case report form in accordance with the above mentioned criteria.

8.5.1 Reporting procedures for SAEs

In the event of serious AEs, the investigator care for or help organizing adequate patient treatment. A written report is also to be prepared and made available to the clinical investigator within five days. The following details should at least be available:

- Patient initials and number
- Patient: date of birth, sex, ethical origin

- The suspected investigational medical product (IMP)
- The adverse event assessed as serious
- Short description of the event and outcome

Reporting to ethics committee /competent authorities has to be done according to the Austrians laws, i.e. summarized at regular intervals of at least one year.

8.5.2 Reporting procedures for SUSARs

It must be remembered that the regulatory authorities, and in case of SUSARs which could possibly concern the safety of the study participants, also the Institutional Review Board / Independent Ethics Committee (IRB / IEC) are to be informed. Such reports shall be made by the study management and the following details should be at least available:

- Patient initials and number
- Patient: date of birth, sex, ethical origin
- Name of investigator and investigating site
- Period of administration
- The suspected investigational medical product (IMP)
- The adverse event assessed as serious and unexpected, and for which there is a reasonable suspected causal relationship to the IMP
- Concomitant disease and medication
- Short description of the event:
  - Description
  - Onset and if applicable, end
  - Therapeutic intervention
  - Causal relationship
  - Hospitalization of prolongation of hospitalization
  - Death, life-threatening, persistent or significant disability or incapacity

Electronic reporting should be the expected method for reporting of SUSARs to the competent authority within legally required time frames.

9 STATISTICAL METHODOLOGY AND ANALYSIS

9.1 Sample size considerations

Sample size calculation is based on the percent change in the main outcome variable "heat shock protein expression" in mesothelial cells after treatment with alanyl-glutamine-dipeptide as add-on to PDF compared to a control group. A sample size of 28 patients in a cross-over study design will have 80% power to detect a difference in means of 30 percentage points, using a 0.05 two-sided significance level (nQuery Advisor version 6). This calculation includes an expected 10% rate of drop-outs and is based on an assumed standard deviation of within-subject period differences of 50 percentage points. Since this standard deviation, however, is unknown a half-sampling will be performed: after the pri-

mary outcome results of a total of 20 patients are available, the standard deviation will be estimated and based on this the sample size and power calculation will be revised.

9.2 Statistical Analyses

Outcome variables will be described by their mean ± standard deviation at the respective time points in the treatment groups. Variables with skew distributions will be transformed appropriately. To compare the percent change in the main outcome variable between the treatment groups, analysis of variance (ANOVA) models for cross-over designs will be used. Additionally, ANOVA models will be used to describe the differences in the secondary outcome variables between the treatment groups. Two-sided p-values smaller than 0.05 will be considered as indicating statistical significance.

The effect of potential drop-outs on the results will be investigated by a sensitivity analysis. For this purpose best- and worst-case scenarios (e.g. first value carried forward) will be analysed and the deviation of their results from a complete-case analysis assessed and discussed."

10 ETHICAL AND LEGAL ASPECTS

10.1 Informed consent of subjects

Following comprehensive instruction regarding the nature, significance, impact and risks of this clinical trial, the patient must give written consent to participation in the study.

During the instruction the patients are to be made aware of the fact that they can withdraw their consent – without giving reasons – at any time without their further medical care being influenced in any way.

In addition to the comprehensive instructions given to the patients by the investigator, the patients also receive a written patient information sheet in comprehensible language, explaining the nature and purpose of the study and its progress.

The patients must agree to the possibility of study-related data being passed on to relevant authorities.

The patients must be informed in detail of their obligations in relation to the participant insurance in order not to jeopardize insurance cover.

10.2 Acknowledgement / approval of the study

The investigator or a designated CRO (Coordinating Centre for Clinical Trials) will submit this protocol and any related documents provided to the subject (such as subject information used to obtain informed consent) to an Ethics Committee (EC) or Institutional Review Board (IRB). Approval from the committee must be obtained before starting the study, and should be documented in a dated letter to the investigator.

Adverse events - whether serious and/or SUSARs and possibly endangering the safety of the study participants - are likewise to be reported to the ethics committee.

The clinical trial shall be performed in full compliance with the valid legal regulations according to the Drug Law (AMG - Arzneimittelgesetz) of the Republic of Austria.

The study must be notified to the Austrian Agency for Health and Food Safety (AGES) and to the European Agency for the Evaluation of Medicinal Products (EMA) and registered to the European Clinical Trial Database (EudraCT) using the required forms.

10.2.1 Protocol amendments

Modifications made to the protocol after receipt of the EC/IRB approval must also be submitted as amendments by the investigator or a designated CRO (Coordinating Centre for Clinical Trials) to the EC/IRB in accordance with local procedures and regulations.

10.3 Finance and insurance

During their participation in the clinical trial the patients will be insured as defined by legal requirements. The principal investigator of the clinical trial will receive a copy of the insurance conditions of the 'patients insurance'. The sponsor is providing insurance in order to indemnify (legal and financial coverage) the investigator/center against claims arising from the study, except for claims that arise from malpractice and/or negligence. The compensation of the subject in the event of study-related injuries will comply with the applicable regulations.

Details on the existing patients insurance are given in the patient information sheet.

This trial is a so-called “academic study”, which means that the costs of the study, the insurance and support of the "Coordination Centre for Clinical Studies (KKS) is funded by the Medical University of Vienna (Department of Child and Adolescent Medicine).

The commercial exploitation of the data is by the Medical University of Vienna.

10.4 Confidentiality

The information contained in this document, especially unpublished data, is the property of the Department of Pediatrics and Adolescent Medicine, Clinical Division of Nephrology and Gastroenterology, Medical University of Vienna. It is therefore provided to you in confidence as an investigator, potential investigator, or consultant, for review by you, your staff, and an Ethics Committee or Institutional Review Board. It is understood that this information will not be disclosed to others without written authorization from the Department of Pediatrics and Adolescent Medicine, Clinical Division of Nephrology and Gastroenterology, Medical University of Vienna, except to the extent necessary to obtain informed consent from those persons to whom the study drug may be administered.

10.5 Ethics and Good Clinical Practice (GCP)

The investigator will ensure that this study is conducted in full conformance with the principles of the "Declaration of Helsinki" (as amended at the WMA General Assembly, Seoul, 2008) and with the laws and regulations of the country in which the clinical research is conducted.

The principal investigator of the clinical trial shall guarantee that only appropriately trained personnel will be involved in the study. This study follows the ICH GCP Guidelines embedded in the Austrian drug act.

11 DOCUMENTATION AND DATA MANAGEMENT

11.1 Documentation of study results

A subject screening and enrollment Log will be completed for all eligible or non-eligible subjects with the reasons for exclusion.

11.1.1 Case report form (CRF)

Data collection will be performed with a central study database. CRFs will be provided for each study participant by the Coordinating Centre for Clinical Trials.

For each study participant enrolled, regardless of study drug initiation, a CRF must be completed and signed by the principal investigator or co-investigator. This also applies to those subjects who fail to complete the study. If a subject withdraws from the study, the reason must be noted on the CRF. Case report forms are to be completed on an ongoing basis.

All forms should be completed using a black pen and must be legible. The entries will be checked by trained personnel (Monitor) and any errors or inconsistencies will be checked immediately. Errors should be crossed out but not obliterated, the correction inserted, and the change initialed and dated by the investigator, co-investigator or study nurse.

The monitor will collect original completed and signed CRFs at the end of the study. A copy of the completed and signed CRFs will remain on site.

11.2 Safekeeping

The investigator will maintain adequate and accurate records to enable the conduct of the study to be fully documented and the study data to be subsequently verified. These documents will be classified into two different categories: investigator's file, and subject clinical source documents.

The investigator's file will contain the protocol/amendments, EudraCT forms, CRFs (eCRF printout), standard operation procedures (SOPs), data clarification and query forms, EC/IRB and Health Authority approval with correspondence, informed consent, drug records, staff curriculum vitae and authorization forms, screening and enrollment logs, and other appropriate documents/correspondence as per ICH/Good Clinical Practice (GCP) and local regulations.

Subject clinical source documents include, but are not limited to subject hospital/clinic records, physician’s and nurse’s notes, appointment book, original laboratory reports, ECG, X-ray, pathology and special assessment reports, consultant letters, etc.

These two categories of documents must be kept on file by the investigator for as long as needed to comply with national and international regulations (in Austria 15 years after discontinuing clinical development or after the last marketing approval). If source documents are not durable as long as needed (e.g. ECG printouts) they must be preserved as a copy. No study document should be destroyed without prior written approval from the Department of Pediatrics and Adolescent Medicine, Clinical Division of Nephrology and Gastroenterology, Medical University of Vienna.

When source documents are required for the continued care of the subject, appropriate copies should be made for storing outside of the site.

11.3 Monitoring

The monitor will contact and visit the investigator regularly and will be allowed, on request, to have access to all source documents needed to verify the entries in the CRFs and other protocol-related

documents provided that subject confidentiality is maintained in agreement with local regulations. It will be the monitor's responsibility to inspect the CRFs at regular intervals throughout the study, to verify the adherence to the protocol and the completeness, consistency and accuracy of the data being entered on them. The monitoring standards require full verification for the presence of informed consent, adherence to the inclusion/exclusion criteria, documentation of SAEs and the recording of the main efficacy endpoints. To be GCP compliant at least 3 monitoring visits are scheduled. An initiation visit, one visit and a final visit after the last patient has finished the study. The monitor will be working according to SOPs and will provide a GCP-compliant monitoring report after each visit for the sponsor and the investigator. Depending on the quality of the data, additional monitoring visits will be necessary according to the sponsor's discretion.

11.4 Relevant Protocol Deviations

All protocol deviations will be listed in the study report and assessed as to their influence on the quality of the study analysis. No deviations from the protocol and of any type will be made without complying with all IRB/EC established procedures in accordance with applicable regulations.

11.5 Publication of study results

The findings of this study will be published by the investigators in a scientific journal and presented at scientific meetings. The manuscript will be circulated to all co-investigators before submission.

11.6 Quality Control and Quality Assurance

11.6.1 Periodic Monitoring

A designed CRA will inspect the paper CRF at regular intervals throughout the study to verify completeness, accuracy and consistency of the data and adherence to the protocol and GCP guidelines. The CRA should have to access to all source data needed to verify the entries in the paper CRFs. The investigator will cooperate with the CRA to ensure that any identified discrepancies are resolved.

11.6.2 Audit and Inspections

Upon request, the investigator will make all study-related source data and records available to a qualified quality assurance auditor mandated by the sponsor or to CA inspectors. The main purpose of an audit or inspection are to confirm that the rights and welfare of the subjects have been adequately protected, and that all data relevant for assessment of safety and efficacy of the investigational product have appropriately been reported to the sponsor

11 REFERENCES

1. FreseniusMedicalCare. Esrd patients in 2009. A global perspective. 2010.
2. Lameire N, Van Biesen W. Epidemiology of peritoneal dialysis: A story of believers and non-believers. Nat Rev Nephrol. 2009; 6:75-82.
3. Devuyst O, Topley N, Williams JD. Morphological and functional changes in the dialysed peritoneal cavity: Impact of more biocompatible solutions. Nephrol Dial Transplant. 2002; 17 Suppl 3:12-5.
4. Jorres A, Topley N, Gahl GM. Biocompatibility of peritoneal dialysis fluids. Int J Artif Organs. 1992; 15:79-83.
5. Schilte MN, Celie JW, Wee PM, Beelen RH, van den Born J. Factors contributing to peritoneal tissue remodeling in peritoneal dialysis. Perit Dial Int. 2009; 29:605-17.
6. Davies SJ, Phillips L, Griffiths AM, Russell LH, Naish PF, Russell GI. What really happens to people on long-term peritoneal dialysis? Kidney Int. 1998; 54:2207-17.
7. Aufricht C. Heat-shock protein 70: Molecular supertool? Pediatr Nephrol. 2005; 20:707-13.
8. Arbeiter K, Bidmon B, Endemann M, Ruffingshofer D, Mueller T, Regele H, Eickelberg O, Aufricht C. Induction of mesothelial hsp-72 upon in vivo exposure to peritoneal dialysis fluid. Perit Dial Int. 2003; 23:499-501.
9. Aufricht C, Endemann M, Bidmon B, Arbeiter K, Mueller T, Regele H, Herkner K, Eickelberg O. Peritoneal dialysis fluids induce the stress response in human mesothelial cells. Perit Dial Int. 2001; 21:85-8.
10. Ruffingshofer D, Endemann M, Arbeiter K, Bidmon B, Mueller T, Herkner K, Aufricht C. Induction of heat shock protein 72 in mesothelial cells exposed to peritoneal dialysate effluent. Perit Dial Int. 2003; 23:74-7.
11. Bidmon B, Endemann M, Arbeiter K, Ruffingshofer D, Regele H, Herkner K, Eickelberg O, Aufricht C. Overexpression of hsp-72 confers cytoprotection in experimental peritoneal dialysis. Kidney Int. 2004; 66:2300-7.
12. Endemann M, Bergmeister H, Bidmon B, Boehm M, Csaicsich D, Malaga-Dieguez L, Arbeiter K, Regele H, Herkner K, Aufricht C. Evidence for hsp-mediated cytoskeletal stabilization in mesothelial cells during acute experimental peritoneal dialysis. Am J Physiol Renal Physiol. 2007; 292:F47-56.
13. Shioshita K, Miyazaki M, Ozono Y, Abe K, Taura K, Harada T, Koji T, Taguchi T, Kohno S. Expression of heat shock proteins 47 and 70 in the peritoneum of patients on continuous ambulatory peritoneal dialysis. Kidney Int. 2000; 57:619-31.
14. Bender TO, Boehm M, Kratochwill K, Vargha R, Riesenhuber A, Witowski J, Joerres A, Wieslander A, Aufricht C. Peritoneal dialysis fluids can alter hsp expression in human peritoneal mesothelial cells. Nephrol Dial Transplant. [Epub ahead of print]
15. Hooper PL. Insulin signaling, gsk-3, heat shock proteins and the natural history of type 2 diabetes mellitus: A hypothesis. Metab Syndr Relat Disord. 2007; 5:220-30.
16. Burkart V, Germaschewski L, Schloot NC, Bellmann K, Kolb H. Deficient heat shock protein 70 response to stress in leukocytes at onset of type 1 diabetes. Biochem Biophys Res Commun. 2008; 369:421-5.
17. Roth E. Nonnutritive effects of glutamine. J Nutr. 2008; 138:2025S-2031S.
18. Wischmeyer PE. Glutamine and heat shock protein expression. Nutrition. 2002; 18:225-8.
19. Eliasen MM, Brabec M, Gerner C, Pollheimer J, Auer H, Zellner M, Weingartmann G, Garo F, Roth E, Oehler R. Reduced stress tolerance of glutamine-deprived human monocytic cells is associated with selective down-regulation of hsp70 by decreased mrna stability. J Mol Med. 2006; 84:147-58.
20. Naka S, Saito H, Hashiguchi Y, Lin MT, Furukawa S, Inaba T, Fukushima R, Wada N, Muto T. Alanyl-glutamine-supplemented total parenteral nutrition improves survival and protein

metabolism in rat protracted bacterial peritonitis model. JPEN J Parenter Enteral Nutr. 1996; 20:417-23.
21. Bender TO, Bohm M, Kratochwill K, Lederhuber H, Endemann M, Bidmon B, Aufricht C. Hsp-mediated cytoprotection of mesothelial cells in experimental acute peritoneal dialysis. Perit Dial Int; 30:294-9.
22. Furst P. New developments in glutamine delivery. J Nutr. 2001; 131:2562S-8S.
23. Wernerman J. Clinical use of glutamine supplementation. J Nutr. 2008; 138:2040S-2044S.
24. Ziegler TR, Ogden LG, Singleton KD, Luo M, Fernandez-Estivariz C, Griffith DP, Galloway JR, Wischmeyer PE. Parenteral glutamine increases serum heat shock protein 70 in critically ill patients. Intensive Care Med. 2005; 31:1079-86.
25. Schroeder S, Lindemann C, Hoeft A, Putensen C, Decker D, von Ruecker AA, Stuber F. Impaired inducibility of heat shock protein 70 in peripheral blood lymphocytes of patients with severe sepsis. Crit Care Med. 1999; 27:1080-4.
26. Weitzel LR, Wischmeyer PE. Glutamine in critical illness: The time has come, the time is now. Crit Care Clin; 26:515-25, ix-x
27. Nakao T, Ogura M, Takahashi H, Okada T. Charge-affected transperitoneal movement of amino acids in CAPD. Perit Dial Int. 1996;16 Suppl 1:S88-90

.

12 APPENDICES

12.1 Appendix IMP

12.1.1 Dipeptiven

2. Qualitative und quantitative Zusammensetzung
1 ml enthält:
Wirkstoff Menge
N(2)-L-Alanyl-
L-Glutamin 200 mg entspr. 82,0 mg L-Alanin und
134,6 mg L-Glutamin
Osmolarität: 921 mosmol/l
Titrationsacidität: 90 – 105 mmol NaOH/l
pH-Wert: 5,4 – 6,0
Die vollständige Auflistung der sonstigen Bestandteile siehe Abschnitt 6.1.
3. Darreichungsform
Konzentrat zur Herstellung einer Infusionslösung.
Klare, farblose Lösung.
4. KLINISCHE ANGABEN
4.1 Anwendungsgebiete
Dipeptiven wird als Teil eines intravenösen Ernährungsregimes angewendet als Zusatz zu Aminosäurenlösungen oder aminosäurenhaltigen Infusionsregimen (z.B. bei Patienten mit hyperkatabolischem und/oder hypermetabolischem Zustand).
4.2 Dosierung, Art und Dauer der Anwendung
Zur zentralvenösen Anwendung nach Zusatz zu einer kompatiblen Infusionslösung. Mischlösungen mit einer Osmolarität über 800 mosmol/l sollten zentralvenös verabreicht werden.
Erwachsene:

Die Dosierung richtet sich nach der Schwere des katabolen Zustandes und nach dem Aminosäurenbedarf. Eine maximale Dosierung von 2 g Aminosäuren/kg Körpergewicht und Tag sollte im Rahmen einer parenteralen Ernährung nicht überschritten werden. Die Zufuhr von Alanin und Glutamin über Dipeptiven ist bei der Berechnung zu berücksichtigen; der Anteil der durch Dipeptiven zugeführten Aminosäuren sollte nicht mehr als ca. 30% der Gesamtaminosäurenzufuhr betragen.

Tagesdosis:

1,5 – 2,0 ml Dipeptiven pro kg Körpergewicht (entsprechend 0,3 – 0,4 g N(2)-L-Alanyl-L-Glutamin pro kg Körpergewicht). Das sind ca. 100 bis 140 ml Dipeptiven für einen Patienten mit 70 kg Körpergewicht.

Maximale Tagesdosis:

2,5 ml Dipeptiven (entsprechend 0,5 g N(2)-L-Alanyl-L-Glutamin) pro kg Körpergewicht.
Die maximale Tagesdosis von 0,5 g N(2)-L-Alanyl-L-Glutamin pro kg Körpergewicht sollte in Kombination mit einer kompatiblen Aminosäurenlösung, die zumindest 1,0 g Aminosäuren pro kg Körpergewicht und Tag zur Verfügung stellt, verabreicht werden. Daraus resultiert eine Tagesdosis von zumindest 1,5 g Aminosäuren pro kg Körpergewicht.
Die folgenden Kombinationen sind Beispiele für die Zufuhr von Dipeptiven und anderen Aminosäuren über die Trägerlösung:
Aminosäurenbedarf 1,2 g/kg Körpergewicht und Tag:
0,8 g Aminosäuren + 0,4 g N(2)-L-Alanyl-L-Glutamin pro kg Körpergewicht und Tag.
Aminosäurenbedarf 1,5 g/kg Körpergewicht und Tag:
1,0 g Aminosäuren + 0,5 g N(2)-L-Alanyl-L-Glutamin pro kg Körpergewicht und Tag.
Aminosäurenbedarf 2 g/kg Körpergewicht und Tag:
1,5 g Aminosäuren + 0,5 g N(2)-L-Alanyl-L-Glutamin pro kg Körpergewicht und Tag.
Die Infusionsgeschwindigkeit richtet sich nach derjenigen der Trägerlösung und beträgt maximal 0,1 g Aminosäuren/kg Körpergewicht und Stunde.
Dipeptiven ist ein Infusionslösungskonzentrat, das nicht direkt verabreicht werden darf, sondern vor der Applikation mit einer kompatiblen Aminosäuren-Trägerlösung oder einem aminosäurenhaltigen Infusionsregime gemischt wird.
1 Volumenanteil Dipeptiven soll mit mindestens 5 Volumenanteilen Trägerlösung gemischt werden (z.B. 100 ml Dipeptiven + mindestens 500 ml Aminosäurenlösung).
Die maximale Konzentration des Wirkstoffes während der Therapie soll 3,5% betragen.
Die Dauer der Anwendung soll 3 Wochen nicht übersteigen.

Kinder:

Die Sicherheit und Wirksamkeit bei Kindern ist nicht gesichert.

4.3 Gegenanzeigen

Dipeptiven darf nicht verabreicht werden bei Patienten mit schwerer Niereninsuffizienz (Creatinin-Clearance <25 ml/ min), schwerer Leberinsuffizienz, schwerer metabolischer Acidose sowie bei bekannter Überempfindlichkeit gegen den Wirkstoff oder einen der sonstigen Bestandteile.

4.4 Besondere Warnhinweise und Vorsichtsmaßnahmen für die Anwendung

Bei Vorliegen einer kompensierten Leberinsuffizienz sind die Leberwerte regelmäßig zu kontrollieren.
Da zurzeit ungenügende Erfahrungen mit der Anwendung von Dipeptiven bei schwangeren und stillenden Frauen sowie bei Kindern vorliegen, wird die Anwendung bei diesen Patienten nicht empfohlen.
Serumelektrolyte, Serumosmolarität, Wasserbilanz, Säuren-Basen-Status sowie die Leberfunktion (alkalische Phosphatase, ALT, AST) sind regelmäßig zu kontrollieren. Es ist auf das mögliche Auftreten einer Hyperammonämie zu achten.
Die Enzyme alkalische Phosphatase, GPT, GOT sowie Bilirubinspiegel und Säuren-Basen-Status sind zu überwachen.
Die Wahl der periphervenösen bzw. der zentralvenösen Applikation hängt von der Osmolarität der zu infundierenden Mischlösung ab. Die allgemein anerkannte Obergrenze für

die periphervenöse Infusion beträgt ca. 800 mosmol/l, hängt aber vom Alter und dem Allgemeinzustand des Patienten sowie von der Beschaffenheit der peripheren Venen ab. Erfahrungen über die Anwendung von Dipeptiven über einen Zeitraum von mehr als 9 Tagen sind begrenzt.

4.5 Wechselwirkungen mit anderen Arzneimitteln und sonstige Wechselwirkungen

Sind bisher nicht beobachtet worden.

4.6 Schwangerschaft und Stillzeit

Dipeptiven sollte aufgrund fehlender Erfahrungen nicht in der Schwangerschaft und Stillzeit angewendet werden.

4.7 Auswirkungen auf die Verkehrstüchtigkeit und die Fähigkeit zum Bedienen von Maschinen

Nicht zutreffend.

4.8 Nebenwirkungen

Bei bestimmungsgemäßer Anwendung nicht bekannt.

4.9 Überdosierung

Wie bei anderen Infusionslösungen, kann es bei zu schneller Infusion von Dipeptiven zu Schüttelfrost, Übelkeit und Erbrechen kommen.
Die Infusion ist in diesem Fall sofort abzusetzen.

5. PHARMAKOLOGISCHE EIGENSCHAFTEN

5.1 Pharmakodynamische Eigenschaften

B05X B02 – Aminosäuren – Infusionslösungskonzentrat
Das Dipeptid N(2)-L-Alanyl-L-Glutamin wird endogen in die Aminosäuren Glutamin und Alanin gespalten und ermöglicht die Zufuhr von Glutamin mit Infusionslösungen zur parenteralen Ernährung. Die freigesetzten Aminosäuren fließen als Nährstoffe in ihre jeweiligen Körperpools ein und werden entsprechend dem Bedarf des Organismus verstoffwechselt. Viele Krankheitszustände, bei denen eine Indikation zur parenteralen Ernährung besteht, gehen mit einer Verarmung des Organismus an Glutamin einher, der Glutamin-hältige Infusionsregime entgegenwirken.

5.2 Pharmakokinetische Eigenschaften

N(2)-L-Alanyl-L-Glutamin wird nach Infusion rasch in Alanin und Glutamin gespalten. Beim Menschen wurden Halbwertszeiten zwischen 2,4 und 3,8 min (bei terminaler Niereninsuffizienz 4,2 min) und eine Plasma-Clearance zwischen 1,6 und 2,7 l/min ermittelt. Das Verschwinden des Dipeptids ist von einem äquimolaren Anstieg der entsprechenden freien Aminosäuren begleitet. Die Hydrolyse erfolgt wahrscheinlich ausschließlich im Extrazellularraum. Die renale Ausscheidung von N(2)-L-Alanyl-L-Glutamin liegt bei Dauerinfusion unter 5% und damit in der gleichen Größenordnung wie die infundierter Aminosäuren.

5.3 Präklinische Daten zur Sicherheit

Akute und subchronische Toxizität:

Untersuchungen im Rahmen der Dosisfindung wurden an Ratten und Hunden über 1 bis 7 Tage durchgeführt. Bei Ratten führte die Infusion von 50 ml/kg KG einer 10-,15-, 20- und 30%-igen Lösung von N(2)-L-Alanyl-L-Glutamin über 4 h/Tag zu tonischen Krämpfen, erhöhter Atemfrequenz und Exitus. Die Infusion von 50 ml/kg KG einer 10%-igen Lösung (5 g N(2)-L-Alanyl-L-Glutamin/kg KG) ergab bei Ratten (6 h/Tag) nekrotische Bezirke an der Infusionsstelle, verringertes Körpergewicht und gelbliche Verfärbungen der Nieren und beim Hund (8 h/Tag) eine vorübergehende Erhöhung der Herzfrequenz. Untersuchungen wurden an Hunden (8 h/Tag) und Ratten (6 h/Tag) mit 0,5 und 1,5 g N(2)-L-Alanyl-L-Glutamin/kg KG und Tag i.v. über 13 Wochen und mit 4,5 g N(2)-L-Alanyl-L-Glutamin/kg KG und Tag i.v. über 6 Wochen durchgeführt. Bei den Hunden kam es zu Erbrechen. Mit der hohen Dosis wurden tonische oder tonisch-klonische Krämpfe, gesteigerte Salivation, Ataxie, Sedierung und Seitenlage beobachtet.

Mutagenes und tumorerzeugendes Potential:

In vitro- und in vivo-Tests ergaben keine Hinweise auf ein mutagenes Potential.

Studien zum tumorerzeugenden Potential wurden nicht durchgeführt. Kanzerogene Wirkungen sind nicht zu erwarten.
Reproduktionstoxizität:
Im Tierversuch ergaben sich bis zu einer Dosis von 1,6 g N(2)-L-Alanyl-L-Glutamin/kg KG und Tag keine Hinweise auf teratogene oder andere embryotoxische Befunde oder peri-postnatale Schädigungen.
Lokale Verträglichkeit:
Nach wiederholter intravenöser Infusion von N(2)-L-Alanyl-L-Glutamin (5 und 10%-ige Lösung) über 13 Wochen kam es bei Ratten und Hunden ab 0,5 g/kg KG zu Unverträglichkeitsreaktionen an den Infusionsstellen (Schwellungen, Verfärbungen, Nekrosen). Histopathologisch wurden bei den Ratten substanzbedingt entzündliche Reaktionen mit leichter bis ausgeprägter Dermatitis purulenta necroticans und Osteomalazie der Schwanzwirbel sowie Thrombophlebitis und Periphlebitis, beim Hund perivaskuläre entzündliche Reaktionen und gelegentlich Gefäßverschluss beobachtet.
Die am Hund durchgeführten Untersuchungen zur lokalen Verträglichkeit nach einmaliger intraarterieller, paravenöser und intramuskulärer Verabreichung ergaben keine Hinweise auf außergewöhnliche Unverträglichkeitsreaktionen bei einer Fehlapplikation.

6. PHARMAZEUTISCHE ANGABEN

6.1 Liste der sonstigen Bestandteile

Wasser für Injektionszwecke.

6.2 Inkompatibilitäten

Das Arzneimittel darf, außer mit den unter Abschnitt 6.6 aufgeführten, nicht mit anderen Arzneimitteln gemischt werden.

6.3 Dauer der Haltbarkeit

2 Jahre.
Dipeptiven sollte nach Anbruch des Behältnisses sofort verbraucht werden.
Dipeptiven darf nach dem Zusatz zu anderen Komponenten nicht gelagert werden.

6.4 Besondere Vorsichtsmaßnahmen für die Aufbewahrung

Nicht über 25° C lagern.
In der Originalverpackung aufbewahren.

6.5 Art und Inhalt des Behältnisses

Glasflasche (Glas Typ II, farblos mit Gummistopfen).
1 x 50 ml, 10 x 50 ml
1 x 100 ml, 10 x 100 ml
Es werden möglicherweise nicht alle Packungsgrößen in den Verkehr gebracht.

6.6 Besondere Vorsichtsmaßnahmen für die Beseitigung und sonstige Hinweise zur Handhabung

Dipeptiven ist ein Infusionslösungskonzentrat, das nicht direkt verabreicht werden darf.
Das Behältnis und die Lösung sind vor der Applikation visuell zu überprüfen. Nur klare, partikelfreie Lösung aus unversehrten Behältnissen verwenden.
Nur zur einmaligen Entnahme.
Bei der Zugabe zu einer Aminosäuren-Trägerlösung vor der Applikation ist auf aseptische Bedingungen und gute Verteilung zu achten.
Gründliche Durchmischung und Kompatibilität müssen gesichert sein.
Nicht verbrauchte Mischlösung ist zu verwerfen.
Dipeptiven wird mit der Trägerlösung infundiert. 1 Volumenanteil Dipeptiven soll mit mindestens 5 Volumenanteilen Trägerlösung gemischt werden (z.B. 100 ml Dipeptiven + mindestens 500 ml Aminosäurenlösung).
Die maximale Konzentration des Wirkstoffes soll während der Therapie 3,5% betragen.

7. Inhaber der Zulassung: Fresenius Kabi Austria, Graz.

8. Zulassungsnummer: 1–21428

9. Datum der Erteilung der Zulassung/Verlängerung der Zulassung: 26. April 1996 / 29. März 2005.

10. Stand der Information: Dezember 2008.
Verschreibungspflicht/Apothekenpflicht: Rezept- und apothekenpflichtig.

12.1.2 Dianeal PD4 Glucose

Fachinformation

Baxter **Dianeal® PD4 Glucose**

1. Bezeichnung der Arzneimittel

Dianeal® PD4 Glucose 1,36 % w/v
Dianeal® PD4 Glucose 2,27 % w/v
Dianeal® PD4 Glucose 3,86 % w/v

2. Verschreibungsstatus/ Apothekenpflicht

Apothekenpflichtig

3. Zusammensetzung der Arzneimittel

3.1 Stoff- oder Indikationsgruppe

Elektrolytlösung mit Glucose

3.2 Arzneilich wirksame Bestandteile nach Art und Menge

1000 ml Lösung enthalten

g/l Lösung	Dianeal® PD4 1,36 % w/v	Dianeal® PD4 2,27 % w/v	Dianeal® PD4 3,86 % w/v
Glucose-Monohydrat (entspr. wasserfreie Glucose)	15,0<br>13,6	25,0<br>22,7	42,5<br>38,6
Natriumchlorid	5,38	5,38	5,38
Natrium-(R,S)-lactat	4,48	4,48	4,48
Calciumchlorid × 2 H_2O	0,184	0,184	0,184
Magnesiumchlorid × 6 H_2O	0,051	0,051	0,051
Elektrolyte in mmol/l			
Natrium	132	132	132
Calcium	1,25	1,25	1,25
Magnesium	0,25	0,25	0,25
Chlorid	95	95	95
Lactat	40	40	40
mosm/l	344	395	483
pH	5,5	5,5	5,5

3.3 Sonstige Bestandteile

Wasser für Injektionszwecke

4. Anwendungsgebiete

Die Anwendung der Lösung ist in Fällen angezeigt, in denen eine Peritonealdialysebehandlung durchgeführt wird, wie z. B. bei akutem und chronischem Nierenversagen, schwerer Wasserretention, Störungen des Elektrolythaushaltes und Arzneimittelvergiftung, sofern eine angemessenere therapeutische Alternative nicht verfügbar ist. Da der Calciumgehalt niedriger als in herkömmlichen Peritonealdialyselösungen ist, eignet sich Dianeal® PD4 besonders zur Anwendung bei Patienten, die mit calciumhaltigen Phosphatbindern behandelt werden und hohe Plasmacalciumwerte haben.

5. Gegenanzeigen

Gegenanzeigen für die Peritonealdialyse sind folgende:
Krankheiten oder Krankheitszustände, die eine ausreichende Proteinernährung unmöglich machen.
Entzündliche Darmerkrankungen, Darmverschluss, Tumore im Bauchraum, frische Bauchverletzungen, Perforation des Zwerchfells, schwere Fettstoffwechselstörungen, beträchtliche intraabdominelle Adhäsionen, chirurgisch bedingte Peritonitis, Verbrennungen im Bereich des Bauches, interne und/oder externe abdominelle Fisteln, Mehrfachoperationen im abdominellen Bereich, generalisierte Sepsis jeglicher Ursache, frische abdominelle Operationen mit offenen Wundflächen, fortgeschrittene Schwangerschaft, bei Patienten mit physischen oder psychischen Bedingungen, die eine den Anweisungen des Arztes gemäße Durchführung der Therapie nicht gewährleisten.
Dianeal® PD4 darf nicht angewendet werden bei Hypokaliämie.

Falls bei Peritonealdialyse-Patienten urämische Symptome auftreten, sollte die Therapie optimiert und ein eventueller katabolischer Zustand identifiziert und behandelt werden. Bei einer Optimierung der Therapie kann man beispielsweise das Austauschvolumen vergrößern oder die tägliche Anzahl an Austauschverfahren erhöhen. Falls dann noch urämische Symptome auftreten, sollte man eine Hämodialyse in Betracht ziehen.

6. Nebenwirkungen

Im Zusammenhang mit der Peritonealdialyse wurde über folgende Nebenwirkungen berichtet:
Nebenwirkungen, die auf die Technik und Durchführung der Dialyse zurückzuführen sind, wie Bauchschmerzen, Blutungen, Bauchfellentzündungen (Peritonitis), Infektionen durch den Katheter, Katheterblockade und Darmverschluss (Ileus), Atemnot, Verstopfung, Diarrhoe, Völlegefühl im Bauchraum.

Nebenwirkungen, die auf die Dialyselösung zurückzuführen sind, wie Elektrolyt- und Wasserimbalancen, verringertes und erhöhtes Blutvolumen (Hypo- und Hypervolämie), erniedrigter und erhöhter Blutdruck (Hypo- und Hypertension) und Muskelkrämpfe. Ferner kann es zu einem Konzentrationsungleichgewicht harnpflichtiger Substanzen zwischen Gewebe und Blut kommen, was zu Übelkeit, Erbrechen, Blutdruckanstieg und unter Umständen Bewußtseinsstörungen führen kann (Dysäquilibrium-Syndrom).

Bei der Peritonealdialyse muss mit einem Verlust an Eiweiß, Aminosäuren und wasserlöslichen Vitaminen gerechnet werden. Bei nicht ausreichender Ernährung oder adäquater Substitutionstherapie kann dies zu Komplikationen führen.

Eine Langzeitanwendung kann Fettstoffwechselstörungen verursachen, vor allem wenn Lösungen mit einem hohen Glucosegehalt verwendet werden. Eine unkontrollierte Anwendung von Dianeal® PD4 3,86 kann zum Wasserverlust (Dehydratation) führen.

Die Überwachung des Plasmaelektrolyt- und Wasserhaushaltes, der Bluteiweiße, Aminosäuren, der wasserlöslichen Vitamine und des Gewichts des Patienten sind erforderlich, damit keine Komplikationen auftreten.
Es empfiehlt sich, bei Patienten, die mit der Peritonealdialyse behandelt werden, die Calcium- und Phosphatwerte im Serum zu überwachen.

Aseptische Bedingungen müssen während des Dialysatwechsels eingehalten werden, um das Infektionsrisiko zu vermindern.
Falls eine Bauchfellentzündung (Peritonitis) auftritt — erkennbar an Schmerzen, Fieber und Trübung des Dialysats — sollte das Dialysat auf Leukozyten untersucht und der Erreger identifiziert werden, um das für die Therapie geeignete Therapeutikum auswählen zu können. Mit einem Breitbandantibiotikum sollte die Therapie sofort begonnen werden.

7. Wechselwirkungen mit anderen Mitteln

Bei der Peritonealdialyse muss damit gerechnet werden, dass Arzneimittel dialysierbar sein können. Eine Korrektur der Dosierung kann erforderlich werden. Bei Patienten unter Digitalistherapie sollten insbesondere die Blutkaliumwerte überwacht und bei der Auswahl der Peritonealdialyselösung berücksichtigt werden. Wegen der bekannten Glucoseresorption sollten die Butzuckerwerte routinemäßig bestimmt werden. Bei Diabetikern, die Insulin oder andere blutzuckersenkende Arzneimittel erhalten, muss die Dosis eventuell korrigiert werden.

8. Warnhinweise

Entfällt.

9. Wichtigste Inkompatibilitäten

Bei Arzneimittelzusätzen ist auf die Kompatibilität der Lösungen zu achten. Arzneimittelzusätze können unverträglich sein und werden nicht empfohlen, da durch Zusätze die sterile Lösung kontaminiert werden kann. Hat sich ein Arzt zu einem medikamentösen Zusatz entschieden, so muss dieser auf aseptischem Wege erfolgen.
In jedem Fall ist darauf zu achten, dass eine vollständige Mischung des Zusatzes mit der Lösung gewährleistet ist. Eine Lagerung solcher Lösungen muss vermieden werden.

10. Dosierung mit Einzel- und Tagesgaben

Der Therapiemodus, die Häufigkeit der Behandlung sowie die Verweilzeit und die Dauer der Dialyse sollten vom behandelnden Arzt festgelegt werden.

Soweit nicht anders verordnet, werden 3 bis 5 Wechsel pro Tag mit je 1500 bis 2000 ml Lösung empfohlen.

Bei Kindern mit einem Körpergewicht (KG) von 10–40 kg sollte die initiale Dosis 10 ml/kg KG mit einer 1,36%igen Glukose-Dialyselösung ohne Verweilzeit betragen. Anschließend sollte das Austauschvolumen allmählich im Verlauf einiger Tage oder Wochen gesteigert werden. Für ein Kind mit einem Körpergewicht von 10, 20, 30 oder

Juni 1998

Fachinformation

Dianeal® PD4 Glucose

Baxter

40 kg sollte eine Dialysatmenge von 400 ml für 10 kg, 600–800 ml für 20 kg, 900–1200 ml für 30 kg bzw. 1200–1500 ml für 40 kg verwendet werden.

Um einen Wasserverlust (Dehydratation) und eine Verringerung des Blutvolumens (Hypovolämie) zu vermeiden und hohen Proteinverlust zu verhindern, sollte man sich bei der Wahl der Lösung an der Wasserbilanz orientieren und Lösungen mit möglichst geringer Hyperosmolarität einsetzen.

11. Art und Dauer der Anwendung

Nur zur intraperitonealen Verabreichung bestimmt. Die Dauer der Anwendung ist vom klinischen Zustand des Patienten abhängig. Sie kann von einigen Tagen bis zu mehreren Jahren betragen.

12. Notfallmaßnahmen, Symptome und Gegenmittel

Entfällt.

13. Pharmakologische und toxikologische Eigenschaften, Pharmakokinetik, Bioverfügbarkeit, soweit die Angaben für die therapeutische Verwendung notwendig sind

13.1 Pharmakologische Eigenschaften

Bei Patienten mit Nierenversagen dient die Peritonealdialyse als Verfahren zum Entfernen von toxischen Substanzen aus dem Stickstoff-Stoffwechsel, die normalerweise über die Nieren ausgeschieden werden und zur Unterstützung bei der Regulierung des Flüssigkeits- und Elektrolythaushaltes sowie des Säure-Base-Gleichgewichtes. Die Peritonealdialyselösung wird über einen Katheter in den Peritonealraum verabreicht. Der Austausch der Substanzen zwischen Dialyselösung und den Peritonealkapillaren des Patienten erfolgt nach dem Prinzip von Osmose und Diffusion über die Peritonealmembran.
Nach einigen Stunden Verweilzeit ist die Lösung mit toxischen Substanzen gesättigt und muss ausgewechselt werden. Mit Ausnahme des Lactats, das als Bicarbonatvorstufe vorliegt, wurden die Elektrolytkonzentrationen in der Lösung so gewählt, dass sie die im Plasma ausgleichen. Stickstoff-Abfallprodukte, die in hohen Konzentrationen im Blut vorliegen, passieren die Peritonealmembran und wandern in die Dialyselösung.
Durch die Glucose wird die Lösung hyperosmolar. Das dadurch entstehende osmotische Gefälle erleichtert den Übertritt von Plasmaflüssigkeit in die Dialyselösung. Somit wird die bei chronischen Nierenpatienten beobachtete Hyperhydratation kompensiert.

13.2 Toxikologische Eigenschaften

Nicht zutreffend.

13.3 Pharmakokinetik

Intraperitoneal verabreichte Glucose wird ins Blut absorbiert und über die üblichen Regelmechanismen metabolisiert.

14. Sonstige Hinweise

Bei schweren, generalisierten infektiösen Hauterkrankungen muss auf die Positionierung des Katheters geachtet werden.
Siehe „Nebenwirkungen".

15. Dauer der Haltbarkeit

Die Haltbarkeit der Dianeal® PD4 1,36 / 2,27 / 3,86 % beträgt 24 Monate.

16. Besondere Lager- und Aufbewahrungshinweise

Nur anwenden, wenn die Lösung klar und der Behälter unversehrt ist. Zur Prüfung nach Entfernen der Schutzhülle den Beutel kräftig drücken. Die Schutzpackung erst vor Gebrauch entfernen.
Nach Entnahme aus dem Umbeutel sollte die Lösung sofort verbraucht werden.
Nach Ablauf des Verfalldatums soll die Lösung nicht mehr verwendet werden.
Restmengen verwerfen.
Die Lagertemperatur sollte 25 °C nicht überschreiten.
Arzneimittel für Kinder unzugänglich aufbewahren.

17. Darreichungsformen und Packungsgrößen

Peritonealdialyselösung in Einfach- oder Doppelbeuteln in den folgenden Packungsgrößen:

Standard (Cycler) /UV

10 und 12 × 250 ml
10 und 12 × 375 ml
10 und 12 × 500 ml
6, 10 und 12 × 750 ml
6 und 8 × 1000 ml
5 und 6 × 1500 ml
4 und 5 × 2000 ml
3 und 4 × 2500 ml
3 × 3000 ml
2 × 5000 ml

IDS/UV

4 und 6 × 1500 ml
4 und 5 × 2000 ml
3 und 4 × 2500 ml
3 × 3000 ml

18. Stand der Information

Juni 1998

19. Name und Anschrift des pharmazeutischen Unternehmers

Baxter Deutschland GmbH
Edisonstr. 3–4
85716 Unterschleißheim

Zentrale Anforderung an:

BPI Service GmbH
FachInfo-Service
Postfach 12 55
88322 Aulendorf

12.1.3 IMP Handling Instruction Ala-Gln in PD

IMP Handling Instruction Ala-Gln in PD

Zuspritzen von Alanyl-Glutamin Dipeptid (Dipeptiven®) in den Einlaufbeutel bei Treatment 1 und 2

Materialvorbereitung:

Desinfektionsmittel (Isozid)

Sterile Nadeln

Sterile Einmalspritzen (20ml)

Händedesinfektionsmittel

Gesichtsmaske

Unsterile Handschuhe

Peritonealdialysat-Beutel (2 Liter) → Dianeal PD4 Glucose 3,86 %

Dipeptivenflasche (50 oder 100 ml einer 20% Lösung (200mg/ml))

Zimmervorbereitung:

Warnschild („Stop“) an die Türe hängen

Zugluft vermeiden

Reinigungsarbeit unterlassen

Arbeitsfläche reinigen, desinfizieren und vorbereiten

Durchführung:

Maske aufsetzen

Hygienische Händedesinfektion lt. Standard

Nadeln und Spritze öffnen (nicht durch Verpackung drücken)

Chemische Händedesinfektion- 30 Sekunden Isozid Einwirkzeit

Stopfen der gebrauchsfertigen Dipeptivenflasche desinfizieren

17,4 ml Dipeptiven-Lösung (= 3,48 g Dipeptid mit Spritze aus Flasche entnehmen

Gummistopfen des PD Einlaufbeutels mit Desinfektionsmittel besprühen

Dipeptivenlösung mit frischer Nadel in 2l Einlaufbeutel spritzen (= 8mmol/l)

Beutel schwenken und auf Klarheit untersuchen (Kompartibilitätsprüfung)

Das gebrauchsfertige Gemisch aus Dipeptiven und PD-Lösung (Endkonzentration beträgt 0,174% = 8mmol/l Alanyl-Glutamin Dipeptid) ist nach der Zubereitung unmittelbar zu verwenden.

Nachbereitung:

Gebrauchte Materialien fachgerecht entsorgen und versorgen

Hygienische Händedesinfektion lt. Standard

Überwachung: Dokumentation in Krankengeschichte

Qualifikation: DGKS/DGKP

Mitarbeiterzahl: 1

Dauer der Maßnahme: 15 Minuten

Quellen: Theoriegeleitete praktische, stationäre und ambulante Erfahrungen

Aktuelle hygienische Richtlinien

Auflage September 2004 Pflegeteam der Kinderdialyse

Anwendungsinformation Dipeptiven Infusionskonzentrat des Universitätsspitals Basel vom 08.05.2008

12.1.4 Application Information Dipeptiven

Universitätsspital Basel

Spital-Pharmazie

Datum:	08.05.08
Seite:	1 von 3
Datei:	AI0007-V01-B01.doc

Dipeptiven Infusionskonzentrat — **Anwendungsinformation**

DIPEPTIVEN Inf Konz 20 g 10 Glasflaschen 100 ml

Zusammensetzung

Infusionskonzentrat 100 ml

Wirkstoff: 1 Flasche zu 100 ml enthält 20 g N(2)-L-Alanyl-L-glutamin entspr. 8,20 g L-Alanin und 13,46 g L-Glutamin

Hilfsstoffe: Aqua ad iniectabilia

Applikationsart

Dipeptiven ist ein **Infusionskonzentrat** und darf nur verdünnt appliziert werden. Dazu wird es einer kompatiblen Infusionslösung zugegeben und anschließend mit dieser infundiert (siehe *„Hinweise zur Zubereitung des Arzneimittels“*, *„Hinweise zur Verabreichung“*).

Hinweise zur Zubereitung des Arzneimittels

Für die sterile Überführung von Dipeptiven in Nutriflex-Beuteln empfehlen wir die Verwendung des Nutriflex Transfersets.

Falls dieses nicht verfügbar ist, kann folgender Aufbau als Alternative verwendet werden (benötigte Bestandteile siehe unter „Zubehör“):

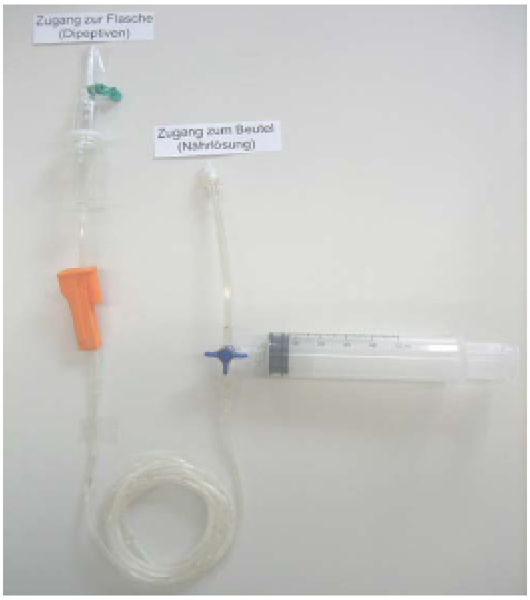

Abb.: Aufbau der Zumischungsapparatur

Für weitergehende Fragen oder bei Unklarheiten konsultieren Sie bitte das aktuelle Arzneimittelkompendium der Schweiz bzw. wenden sich an den Dienstpharmazeuten der Klinikbetreuung unter Tel. 061 / 265 29 13.

MEDIZINISCHE UNIVERSITÄT WIEN

Universitätsspital Basel

Spital-Pharmazie

Datum:	08.05.08
Seite:	2 von 3
Datei:	AI0007-V01-B01.doc

Dipeptiven Infusionskonzentrat — **Anwendungsinformation**

Ablauf des Aufbaus der Zumischungsapparatur:

- Dreiwegehahn mit Schlauch:
  - am Schlauchende Injektionsnadel anschrauben (Schutzkappe bleibt auf Injektionsnadel)
  - am seitlichen Anschluss die Perfusorspritze (oder andere Spritze mit Luer-Lock-Anschluss) anschrauben
  - den unteren Anschluss mit dem Schlauchende des Infusionsbestecks verbinden
- die Spitze des Infusionsbestecks durch den desinfizierten Stopfen der gebrauchsfertigen Dipeptiven-Flasche stechen
- die Dipeptiven-Flasche mit der Öffnung nach unten hoch halten, am Infusionsbesteck die Belüftung öffnen, das Dipeptiven durch das Infusionsbesteck bis zum Dreiwegehahn laufen lassen und dann den Dreiwegehahn auf Durchlass Perfusorspritze-Infusionsbesteck stellen
- das Septum des Infusionsbeutels desinfizieren und die Injektionsnadel (an der Spitze vom Schlauchende des Dreiwegehahns) durch das Septum stechen
- mit der Perfusorspritze das Dipeptiven aufziehen
- Dreiwegehahn auf Durchgang Perfusorspritze-Infusionsbeutel stellen und das Dipeptiven zum Beutelinhalt zumischen
- Aufziehen und Entleeren der Perfusorspritze so oft wiederholen (Stellung des Hahnes stets beachten), bis das entsprechende Volumen zugespritzt wurde
- nach der Beendigung der Zumischung den Dreiwegehahn so einstellen, dass kein Durchlauf aus irgendeiner Richtung stattfinden kann (Hahnflügel zeigen in Zwischenräume)
- Perfusorspritze und Infusionsbesteck können abgeschraubt werden, der Dreiwegehahn mit Schlauch verbleibt im Infusionsbeutel und kann weiterhin als Zugang zum Beutel genutzte werden.

→ *Hinweise zum Arbeiten mit dem Dreiwegehahn finden sie auch im Intranet unter „Dokumentation", „Pflegerichtlinien", „Inhaltsverzeichnis", „Kapitel 13.3.4 Dreiwegehahn".*

Hinweise zur Verabreichung

Dipeptiven ist ein Infusionskonzentrat, welches nur verdünnt in einer kompatiblen Aminosäurenlösung oder einem aminosäurehaltigen Infusionsregime appliziert werden darf. 1 Volumenanteil Dipeptiven soll mit mindestens 5 Volumenanteilen Trägerlösung gemischt werden. Nutriflex-Produkte (Lipid plus, Lipid spezial, Lipid spezial ohne Elektrolyte, spezial) müssen stets zentralvenös verabreicht werden und haben vom Hersteller vorgegebene maximale Zumischungsvolumina.

Folgende Volumina Dipeptiven können den einzelnen Nutriflex-Produkten zugesetzt werden:

- 1250 ml Nutriflex Lipid (plus, spezial, spezial ohne Elektrolyte): max. 100 ml Dipeptiven
- 1875 ml Nutriflex Lipid (plus, spezial, spezial ohne Elektrolyte): max. 200 ml Dipeptiven
- 2500 ml Nutriflex Lipid (plus, spezial, spezial ohne Elektrolyte): max. 300 ml Dipeptiven
- 1500 ml Nutriflex spezial: max. 100 ml Dipeptiven

Bei folgenden Produkten kann eine Zumischung von Dipeptiven erfolgen:

- alle Nutriflexbeutel (Lipid plus, Lipid spezial, Lipid spezial ohne Elektrolyte, spezial)
- Lipovenös 20 %
- Glucose-Lösungen (bis einschließlich 40 % Glucose-Konzentration)

Welche Zusätze (z.B. Vitamine, Spurenelemente, Insulin, u.a.) in welchen Mengen zu der Mischung aus Dipeptiven und einem Nutriflex-Produkt zugemischt werden können, entnehmen Sie bitte der Nutriflex-Übersicht unter: http://www.spitalpharmazie-basel.ch/dienstleistungen/pdf/Nutriflex-uebersicht.pdf

Für weitergehende Fragen oder bei Unklarheiten konsultieren Sie bitte das aktuelle Arzneimittelkompendium der Schweiz bzw. wenden sich an den Dienstpharmazeuten der Klinikbetreuung unter Tel. 061 / 265 29 13.

Vorsichtsmassnahmen / Monitoring

Schüttelfrost, Übelkeit und Erbrechen sind Anzeichen für eine zu schnelle Infusion von Aminosäure-Lösungen. Es ist auf das Auftreten einer Hyperammonämie zu achten. Um diese zu vermeiden, darf die maximale Zufuhrrate von 2 g Aminosäuren/kg Körpergewicht/Tag nicht überschritten werden. Eine Hyperammonämie kann u.a. durch einen Proteinüberschuss, Magen-Darm-Blutungen, Leber- und/oder Nierenfunktionsstörungen entstehen. Das Auftreten einer Hyperammonämie ist am süsslichen Atemgeruch zu erkennen und wird auf Grund weiterer Symptome in 3 Stadien eingeteilt:

1. Muskelschwäche, sinkender Muskeltonus, leichte Verwirrtheit
2. schwere Verwirrtheit, Bewegungsunfähigkeit
3. Koma, Reflexlosigkeit

Inkompatibilitäten

Beim Zumischen von Dipeptiven zu Trägerlösungen im Rahmen der parenteralen Ernährung ist auf physikalisch-chemische Veränderungen (Ausfällungen, Verfärbungen) und generelle Verträglichkeit (Kompatibilität) zu achten. Eine Zugabe von Dipeptiven zu weiteren unter „Hinweise zur Verabreichung“ nicht genannten Lösungen wurde vom Hersteller nicht getestet und kann daher nicht empfohlen werden.

Haltbarkeit nach Anbruch

Dipeptiven ist bei Raumtemperatur (15 - 25 °C) zu lagern. Nur klare Lösungen aus unversehrten Behältnissen verwenden. Auf keinem Fall darf Dipeptiven nach dem Zusatz zu anderen Komponenten gelagert werden. Die gebrauchsfertige Infusionsmischung aus Dipeptiven und Nutriflexbeuteln (Lipid plus, Lipid spezial, Lipid spezial ohne Elektrolyte, spezial) ist nach der Zubereitung unmittelbar zu verwenden und max. 24 Stunden bei Raumtemperatur haltbar.

Zubehör

Zubehör zum Zumischen von Dipeptiven zu Nutriflexbeuteln:

- Nutriflex Transferset (SAP-Nr.: 9067246
- Perfusorspritze 50 ml (SAP-Nr.: 1134955)
- Dreiwegehahn mit Schlauch für Infusionstherapie (Discofix C-3, B.Braun, SAP-Nr.: 9059366)
- Infusionsbesteck (Intrafix Primeline Comfort, B.Braun, SAP-Nr.: 9025518)

Quellen

- Kompendiumstexte zu Dipeptiven und Nutriflex-Produkten
- Fachinformationen von Fresenius-Kabi (CH) AG zu Dipeptiven-Infusionslösungskonzentrat
- Informationen von B.Braun Medical AG zur Mischung von Nutriflex Lipid mit Dipeptiven
- Homepage der Spital-Pharmazie (Link unter: Dienstleistungen → Arzneimittelinformationen → Kompatibilität von Zusätzen zu Nutriflexbeuteln)
- Intranet des USB (Link unter: Dokumentation → Pflegerichtlinien → Inhaltsverzeichnis → Kapitel 13.3.4 Dreiwegehahn)

12.1.5 Verification of Stability

Stabilitätstestung (17,4 ml Dipetiven® in 2l PD4 Peritonealdialyselösung PD4)

Die modifizierte Peritonealdialyselösung wurde in Magistraliter-Formulierungen hergestellt. Zwei Chargen wurden von Prof. Gabor vom Department für Pharmazeutische Technologie und Biopharmazie der Universität Wien einer ausführlichen Stabilitätstestung unter verschiedenen Bedingungen unterzogen.

Methodik: High-performance liquid chromatography (HPLC)- ESI MS/MS in MRM Mode mit einer 250 x 3 mm Säule zur Auftrennung und Analyse von Ala-Gln plus Aminogramm der proteinogenen Aminosäuren bei vorheriger Derivatisierung der Proben. Quantifizierung über eine Standardverdünnungsreihe von Ala-Gln plus Isotopengemisch für freie Aminosäuren.

Resultat: Alle Messungen erfolgten in Triplikaten.

Charge (vom)	Lager-bedingungen	Analyse- datum	Lagerzeit	Ala-Gln (mMol/L)	CV%
05.10.2009	-80°	30.12.2009	[-80°]	7.96 ± 0.45	5.69
04.09.2009	RT	11.10.2009	4 Wochen	8.07 ± 0.14	1.73
05.10.2009	-80°/RT	20.01.2010	4 Wochen	7.99 ± 0.48	5.99
05.10.2009	RT	30.12.2009	12 Wochen	8.03 ± 0.23	2.82
05.10.2009	RT	20.01.2010	15 Wochen	8.06 ± 0.13	1.57
05.10.2009	-80°/37°	20.01.2010	4 Wochen	7.69 ± 0.41	5.29
05.10.2009	37°	30.12.2009	12 Wochen	7.89 ± 0.13	1.63
05.10.2009	37°	20.01.2010	15 Wochen	8.17 ± 0.32	3.97

Interpretation: Unter Raumtemperatur konnte während der Beobachtungszeit (bis 15 Wochen) eine vollständige Stabilität bestätigt werden, unter 37°C waren die Schwankungsbereiche höher, dennoch war kein Hinweis auf Instabilität zu sehen. Neben dem Nachweis von Alanyl-Glutamin in der ursprünglich für die Mischung vorgesehenen Konzentration von 8 mmol / l = 0,174 % ± Messschwankung konnte als Bestätigung für die hohe Stabilität dieses Produktes auch der Beweis erbracht werden, dass im Aminogramm der Proben auch keine Spaltprodukte, insbesondere weder Alanin noch Glutamin als Säure nachweisbar waren.
