# Supplementary material for: Addition of Alanyl-Glutamine to Dialysis Fluid Restores Peritoneal Cellular Stress Responses – A First-In-Man Trial
Source: PLoS One. 2016 Oct 21;11(10):e0165045. doi: 10.1371/journal.pone.0165045 (PMC5074513; doi:10.1371/journal.pone.0165045)
Supplement: S1 Table — Monoisotopic masses, calculated and observed, their Chemspider IDs, HMDB IDs and KEGG IDs as queried from the databases and the (putative) chemical entities. a Up-and down-regulation was defined either as significant difference at time point 4 h, or in the correlation with time, or as qualitative difference with vs. without AlaGln (see Methods for details). (PDF) [file pone.0165045.s005.pdf]

S1 Table. Putative chemical entities changed significantly in abundance following treatment with standard PDF *vs.* PDF with 8 mM AlaGln

Monoisotopic Mass (Da)	Observed Mass (Da)		Δppm		RetentionTime (min)		Absolut Frequency		Chemspider ID	KEGG Compound ID	HMDB ID	Chemical Entity	Regulation[a]
	Mean [MH+]	± SD	Mean	± SD	Mean	±SD	A	B					
131.09464	132.10076	1.77E-03	1.22	4.59E-01	1.17647	7.47E-03	32	32	5880	C00123	HMDB00687	L-(+)-Leucine	up
	132.10076	1.77E-03	1.22	4.59E-01	1.17647	7.47E-03	32	32	6067	C00407	HMDB00172	L-(+)-Isoleucine	up
	132.10077	1.77E-03	1.22	4.62E-01	1.17647	7.47E-03	32	32	19964	C02378	HMDB01645	Norleucine	up
	132.10076	1.77E-03	1.22	4.59E-01	1.17647	7.47E-03	32	32	388796	C02486	HMDB03640	beta-Leucine	up
131.09500	132.10075	1.77E-03	4.01	4.42E-01	1.19632	7.47E-03	32	32	548	C02378	HMDB01901	Aminocaproic acid	up
146.06914	147.07606	7.15E-05	2.40	4.89E-01	0.11454	7.09E-03	28	29	5746	C00064	HMDB00641	L-Glutamine	up
	147.07606	7.15E-05	2.40	4.89E-01	0.11454	7.09E-03	28	29	128633	C00819	HMDB03423	D-Glutamine	up
	147.07606	7.15E-05	2.40	4.89E-01	0.11454	7.09E-03	28	29	141172	C05100	HMDB02031	Ureidoisobutyric acid	up
164.04735	165.05428	1.38E-04	0.70	4.47E-01	0.05406	2.64E-03	21	24	972	C00166	HMDB00205	Phenylpyruvic acid	down
	165.05428	1.38E-04	0.70	4.47E-01	0.05406	2.64E-03	21	24	671	C02763	HMDB01237	2-Hydroxy-3-phenylacrylic acid	down
	165.05428	1.38E-04	2.11	8.41E-01	0.05406	2.64E-03	21	24	556910	C02763	HMDB12225	Enolphenylpyruvic acid	down
	165.05428	1.38E-04	2.11	8.41E-01	0.05406	2.64E-03	21	24	553146	C01772	HMDB02641	o-Coumaric acid	down
	165.05428	1.38E-04	2.11	8.41E-01	0.05406	2.64E-03	21	24	553147	C12621	HMDB01713	m-Coumaric acid	down
	165.05428	1.38E-04	2.11	8.41E-01	0.05406	2.64E-03	21	24	553148	C00811	HMDB02035	p-Coumaric acid	down
164.08373	165.09075	6.21E-05	1.52	3.79E-01	1.80953	1.10E-02	32	32	6745	C20327	HMDB00329	2-Phenylbutanoic acid	up
180.04225	181.04918	8.69E-05	1.91	4.83E-01	2.62288	7.31E-01	32	32	19207	n/a	HMDB02130	Monomethyl Phthalate	up
181.07390	182.08091	5.58E-05	1.45	3.08E-01	0.01699	7.27E-03	31	31	5833	C00082	HMDB00158	Tyrosine	down
	182.08091	5.58E-05	1.45	3.08E-01	0.01699	7.27E-03	31	31	389285	C04368	HMDB03831	3-Amino-3-(4-hydroxyphenyl)propanoic acid	down
	182.08091	5.58E-05	1.45	3.08E-01	0.01699	7.27E-03	31	31	13628311	C03290	HMDB02184	L-threo-3-phenylserine	down
181.07401	182.08091	5.58E-05	2.04	3.08E-01	0.01699	7.27E-03	31	31	425	C19579	HMDB01119	4-Hydroxy-4-(3-pyridinyl)butansäure	down
183.02014	184.02788	7.68E-05	2.51	4.20E-01	2.90961	1.40E-02	21	26	154529	C16511	HMDB02205	(2S)-2-amino-4-sulfobutanoic acid	down

217.10600	218.11320	7.90E-05	0.41	2.78E-01	0.01316	7.09E-03	32	0	571213	C15532	HMDB00856	N-Acetyl-L-Citrulline	up
244.06953	245.07793	8.50E-05	4.58	3.48E-01	2.39326	1.56E+00	13	19	5807	C00299	HMDB00296	Uridine	down
	245.07793	8.50E-05	4.58	3.48E-01	2.39326	1.56E+00	13	19	14319	C02067	HMDB00767	pseudouridine	down
251.10184	252.10865	9.19E-05	1.86	3.66E-01	0.99543	1.03E-02	31	31	13135	C00559	HMDB00101	2'-Deoxyadenosine	down
	252.10865	9.19E-05	1.86	3.66E-01	0.99543	1.03E-02	31	31	388325	C05198	HMDB01983	5'-deoxyadenosine	down
252.20892	253.21486	1.11E-03	1.41	3.71E-01	2.25196	5.54E-02	19	20	13628094	n/a	HMDB00477	7Z,10Z-Hexadecadienoic acid	up
267.09674	268.10366	3.70E-04	1.84	5.35E-01	1.00211	5.29E-02	23	26	163230	C00330	HMDB00085	Deoxyguanosine	down
330.25589	331.26259	1.17E-04	1.74	3.55E-01	2.66936	6.43E-03	31	31	4942831	C16513	HMDB01976, HMDB60113	Docosapentaenoic acid	up
358.30832	360.16748	4.91E+00	1.75	3.70E-01	2.60498	8.30E-02	32	31	71407	n/a	HMDB11535	Glycerin monostearate	up
	360.16748	4.91E+00	1.75	3.70E-01	2.60498	8.30E-02	32	31	10381543	C01885	HMDB11131	(2S)-2,3-Dihydroxypropyl stearate	up
783.48389	392.74793	1.61E-04	3.29	4.10E-01	1.28891	4.22E-02	20	14	24768672	n/a	n/a	(3Z,6Z,9Z,12Z,21R)-27-Amino-24-hydroxy-24-oxido-18-oxo-19,23,25-trioxa-24lambda~5~-phosphaheptacosa-3,6,9,12-tetraen-21-yl (4Z,7Z,10Z,13Z,16Z,19Z)-4,7,10,13,16,19-docosahexaenoate	up
	392.74793	1.61E-04	3.29	4.10E-01	1.28891	4.22E-02	20	14	24768918	n/a	n/a	(2R)-3-{[(2-Aminoethoxy)(hydroxy)phosphoryl]oxy}-2-[(5Z,8Z,11Z,14Z,17Z)-5,8,11,14,17-icosapentaenoyloxy]propyl (5Z,8Z,11Z,14Z,17Z)-5,8,11,14,17-icosapentaenoate	up

392.74793	1.61E-04	3.29	4.10E-01	1.28891	4.22E-02	20	14	24769134	n/a	n/a	(2R)-3-{[(2-Aminoethoxy)(hydroxy)phosphoryl]oxy}-2-[(6Z,9Z,12Z,15Z)-6,9,12,15-octadecatetraenoyloxy]propyl (4Z,7Z,10Z,13Z,16Z,19Z)-4,7,10,13,16,19-docosahexaenoate	up

Legend to Supplementary S1 Table: Monoisotopic masses, calculated and observed, their Chemspider IDs, HMDB IDs and KEGG IDs as queried from the databases and the (putative) chemical entities. [a] Up-and down-regulation was defined either as significant difference at time point 4 h, or in the correlation with time, or as qualitative difference with *vs.* without AlaGln (see Supplemental Methods for details).
