# Supplementary material for: Addition of Alanyl-Glutamine to Dialysis Fluid Restores Peritoneal Cellular Stress Responses – A First-In-Man Trial
Source: PLoS One. 2016 Oct 21;11(10):e0165045. doi: 10.1371/journal.pone.0165045 (PMC5074513; doi:10.1371/journal.pone.0165045)
Supplement: S2 Table — AE = Adverse Event; Calculations are based on the total number of AEs (N = 42). One mild AE (mild diarrhea) could not be assigned to treatment A or B since it persisted during both study phases. (PDF) [file pone.0165045.s006.pdf]

S2 Table. Summary of adverse events.

System organ class	Lower level term	Mild AE		Moderate AE		Severe AE	
		pure PDF	+ AlaGln	pure PDF	+ AlaGln	pure PDF	+ AlaGln
Blood and lymphatic system disorders	Anemia aggravated	-	-	-	1 (2.4%)	-	-
Gastrointestinal disorders	Gingivitis	-	1 (2.4%)	-	-	-	-
	Left upper quadrant pain	-	1 (2.4%)	-	-	-	-
	Vomiting	-	1 (2.4%)	-	-	-	-
	Diarrhea	1 (2.4%)	-	-	-	-	-
	Heartburn	-	1 (2.4%)	-	-	-	-
General disorders and administration site conditions	Product contamination	-	-	-	1 (2.4%)	-	-
	Weakness	-	-	-	1 (2.4%)	-	-
Infections and infestations	Common cold	-	1 (2.4%)	-	-	-	-
	Influenza	1 (2.4%)	-	-	-	-	-
Injury, poisoning and procedural complications	Procedural pain	-	2 (4.8%)	-	-	-	-
Investigations	Anticoagulation level above therapeutic	-	-	-	-	-	1 (2.4%)
	Blood pressure increased	3 (7.1%)	2 (4.8%)	3 (7.1%)	3 (7.1%)	-	-
	Peritoneal effluent leukocyte count increased	-	-	1 (2.4%)	-	-	-
Metabolism and nutrition disorders	Hyperkalemia	-	1 (2.4%)	-	1 (2.4%)	-	-
	Water retention	-	1 (2.4%)	-	-	-	-
Musculoskeletal and connective tissue disorders	Cramps leg	1 (2.4%)	1 (2.4%)	-	-	-	-
	Pain neck/shoulder	1 (2.4%)	1 (2.4%)	-	-	-	-
	Painful hips	1 (2.4%)	-	-	-	-	-
Nervous system disorders	Dizziness	1 (2.4%)	3 (7.1%)	-	1 (2.4%)	-	-
Renal and urinary disorders	Renal function aggravated	-	-	1 (2.4%)	-	-	-
Reproductive system and breast disorders	Pain menstrual	1 (2.4%)	-	-	-	-	-
Vascular disorders	Hypotension	-	1 (2.4%)	-	-	-	-

	Hypertension	-	-	1 (2.4%)	-	-	-
**Total**		**10(23.8%)**	**17 (40.5%)**	**6 (14.3%)**	**8 (19%)**	**-**	**1 (2.4%)**

Legend to Supplementary S2 Table: AE = Adverse Event; Calculations are based on the total number of AEs (N=42).

One mild AE (mild diarrhea) could not be assigned to treatment A or B since it persisted during both study phases.
